# The potential of piR-823 as a diagnostic biomarker in oncology: A systematic review

**DOI:** 10.1371/journal.pone.0294685

**Published:** 2023-12-07

**Authors:** Eun Jung Sohn, Myoung-Eun Han, Young Mok Park, Yun Hak Kim, Sae-Ock Oh

**Affiliations:** 1 Research Center for Molecular Control of Cancer Cell Diversity, Pusan National University, Yangsan, Republic of Korea; 2 Department of Anatomy, School of Medicine, Pusan National University, Yangsan, Republic of Korea; 3 Department of Surgery, School of Medicine, Pusan National University, Yangsan, Republic of Korea; 4 Department of Biomedical Informatics, School of Medicine, Pusan National University, Yangsan, Republic of Korea; Qatar Biomedical Research Institute, QATAR

## Abstract

**Background:**

Emerging evidence has demonstrated that PIWI-interacting RNAs (piRNAs) play important roles in various physiological processes and contribute to cancer progression. Moreover, piRNAs and PIWI protein levels are associated with the prognosis and chemoresistance of various cancers. The limitations of biomarkers challenge early detection and monitoring of chemoresistance and cancer relapse.

**Methods:**

To evaluate the potential of piRNA as a diagnostic biomarker in oncology, we systematically reviewed previous studies on the subject. PubMed, Embase, and Cochrane databases were searched to evaluate the diagnostic relevance of piRNAs in cancer. Eighteen studies (2,352 patients) were included. The quality of each study was evaluated with AMSTAR and QUADAS-2 tool.

**Results & conclusions:**

The area under the curve (AUC) values of 26 piRNAs in patients with cancer ranged from 0.624 to 0.978, with piR-9491 showing the highest value (0.978). The sensitivity of the total of 21 piRNAs in cancer patients was between 42.86 and 100, with piR-9491 showing the highest sensitivity (100). The specificity of these 21 piRNAs ranged from 60.10 to 96.67 (with piR-018569 showing the highest specificity (96.67)). Their odds ratios were between 1.61 and 44.67, and piR-12488 showed the highest odds ratio (44.67). Generally, the piRNAs in this review showed better sensitivity and AUC values than current clinical diagnostic biomarkers, although current biomarkers appear to be more specific. Reviewed piRNAs showed better diagnostic performance than currently used clinical biomarkers. Notably, piR-823 showed a significant diagnostic performance in four types of cancer (colorectal, esophageal, gastric, and renal cell cancer). However, all 18 studies included in this review were a case-control study. So, further prospective studies are required for their validation.

## Introduction

PIWI-interacting RNAs (piRNAs) have been shown to play critical roles in many physiological and pathological processes since their discovery in germ cells in 2006 [1]. piRNAs are small non-coding RNA molecules consisting of 23–31 nucleotides and are mainly involved in the regulation of gene expression and maintenance of genomic integrity. Many studies have shown that piRNAs differ from miRNAs or siRNAs in terms of their chemical structure, size, processing, precursors, associated proteins, and functional mechanisms [2]. piRNAs have 2’-O-methylation at their 3’-end and bind to a PIWI protein, an Argonaute/PIWI family protein, to form the piRNA-induced silencing complex (piRISC). The main functional mechanisms by which piRNAs regulate gene expression involve regulation of RNA degradation and modification of chromatin structure and histone proteins. They can suppress transposable elements (TEs), which are DNA sequences that move around the genome and disrupt gene function [3] through cleavage following base pairing with their target genes. Notably, they can also target non-transposon (protein-coding) genes. For example, mouse pachytene piRNAs can cleave target mRNA together with the mouse PIWI homolog (MIWI) protein through an siRNA-like mechanism [4]. Interestingly, they regulate mRNA stability by promoting deadenylation and decay. Mouse pachytene piRNAs can recruit deadenylase CAF1 to late spermatids [5]. In addition, they can modify the chromatin structure and histone proteins by interacting with DNA [6] and histone methylation machinery [7, 8].

Critical roles of piRNAs have been suggested in a variety of physiological processes, including germline cell biology, development, neuronal function, and immune responses [9–15]. They have also been associated with pathological conditions, including cancer [16], reproductive diseases, retinal diseases, cardiovascular diseases, and neurodegenerative diseases [17, 18]. Abnormal piRNA expression has been observed in many cancer types, including breast cancer, colorectal cancer, glioma, lymphoma, and multiple myeloma. Some oncogenic piRNAs, including piR-36026, piR-54265, piR-33221, and piR823, promote tumor progression by increasing cell proliferation, migration, and invasion, while inhibiting apoptosis. However, some tumor-suppressor piRNAs, including piR-36712, inhibit tumor development. piR-36712 overexpression in breast cancer cells has been shown to decrease cell proliferation and tame malignant phenotypes, while its knockdown increases cancer cell proliferation.

The potential use of piRNAs as biomarkers for various diseases, including cancer and cardiovascular, neurodegenerative, and infectious diseases, has been suggested. Notably, owing to their high stability, the potential of piRNAs as non-invasive biomarkers in body fluids has been evaluated. It is possible that the 2’-O-methyl modification in piRNAs may be stabilizing by protecting against 3’ uridylation and truncation [19, 20]. Notably, piRNAs have been observed in human body fluids, including serum and plasma samples, and remain stable after long-term incubation at room temperature [21–26]. Early detection and monitoring of chemoresistance and cancer relapse are complicated by the limitations of current biomarkers. Therefore, in this review, we evaluated the potential of piRNAs as diagnostic biomarkers for cancer.

## Methods

This systematic review was conducted according to the Preferred Reporting Items for Systematic Reviews and Meta-Analyses (S1 Table, PRISMA) statement [27].

### Search strategy

Relevant literature was identified through searches in the PubMed, Embase, and Cochrane libraries. All literature we have identified from three libraries is presented in the supporting information. The keywords used for retrieval included: (1) PIWI-interacting RNA or piRNA; and (2) neoplasm, cancer, carcinoma, malignancy, or tumor; and (3) sensitivity, specificity, or biomarker. The retrieved literature was published from 2006 to 2023 (Apr).

### Eligibility criteria

We included both cohort (prospective and retrospective) studies as well as case-control studies. Specifically, we focused on studies that investigated the diagnostic value of piRNAs to extract and calculate key indicators directly or indirectly, including sensitivity, specificity, true positive (TP), false positive (FP), false negative (FN), and true negative (TN). The diagnosis of cancer was based on general guidelines for each cancer, such as histopathology or other appropriate diagnostic criteria. However, we excluded studies that provided incomplete diagnostic information or analyzed previously published papers, such as meta-analyses, systematic reviews, case reviews, case reports and letters. Articles published in languages other than English were excluded from the analysis. Additionally, the lists of authors, centers of study, and recruitment intervals of the included studies were compared to identify duplicate reports of the same research or studies with a substantial overlap in their study populations. In such cases, less informative studies were excluded from the analysis.

### Study selection

To eliminate personal bias, two reviewers independently conducted the search process. Any disagreements were resolved through discussions with a third author. At the first stage we screened the titles and/or abstracts of the search results and selected possibly relevant studies for the second stage of the study selection process. In the second stage, after excluding conference abstracts, we retrieved and evaluated the full texts in detail. Subsequently, the studies that met the eligibility criteria were included in the analysis.

### Data extraction and quality assessment

Two authors independently screened each retrieved record and performed data extraction. Any differences were resolved through discussions with a third author until a consensus was reached. The following information was extracted from the literature: author; year of publication; patient country; comprehensive sensitivity and specificity of the biomarkers; TP, FP, FN, and TN; type; and number of cases. Finally, the area under the curve (AUC), sensitivity, specificity, and diagnostic odds ratios were evaluated. All included literature was summarized in Table 1. AMSTAR checklist(S2 Table, [28]) was used to assess the quality of this systemic review. In addition, QUADAS-2 checklist (S3 Table, [29]) was used to assess the quality of included studies.

**Table 1:** Main characteristics of the articles included in this study.

Cancer Type	Study ID (Ref)	piRNA	Region	Case	Control	TP	FP	FN	TN
**Colorectal Ca**	Wang, Z 2020 [24]	**piR-020450**	China	180	180	139	30	41	150
		**piR-020619**	China	180	180	149	25	31	155
		**CEA**	China	180	180	74	12	106	168
		**CA19-9**	China	180	180	50	8	130	172
	Iyer, DN 2020 [49]	**piR-24000**	China	87	87	81	27	6	60
	Vychytilova-Faltejskova, P 2018 [23]	**piR-5937**	Czech	179	100	132	35	47	65
		**piR-28876**	Czech	179	100	118	35	61	65
	Sabbah, NA 2021 [50]	**piR-823**	Egypt	84	75	70	8	14	67
	Mai, D 2020 [51]	**piR-54265**	China	725	209	621	73	104	136
	Qu, A 2019 [52]	**piR-017724**	China	220	220	120	34	100	186
		**CEA**	China	220	220	92	12	128	208
		**CA19-9**	China	220	220	42	8	178	212
	Li, J 2022 [53]	**piR-31143**	China	13	8	10	0	3	8
**Esophageal Ca**	Su, JF 2020 [37]	**piR-823**	China	54	54	34	12	20	42
**Gastric Ca**	Ge, L 2020 [54]	**piR-019308**	China	70	60	40	5	30	55
		**piR-018569**	China	70	60	31	2	39	58
		**piR-004918**	China	70	60	30	3	40	57
		**CEA**	China	70	60	37	6	33	54
		**CA199**	China	70	60	28	1	42	59
		**AFP**	China	70	60	40	20	30	40
	Cui, L 2011 [21]	**piR-651**	China	93	32	66	6	27	26
		**piR-823**	China	93	32	75	6	18	26
	Zhou, X 2020 [55]	**piR-1245**	China	66	66	60	17	6	49
**Glioblastoma**	Bartos, M 2021 [56]	**piR-9491**	Czech	77	23	77	4	0	19
		**piR-12488**	Czech	77	23	67	3	10	20
		**piR-1849**	Czech	77	23	64	6	13	17
		**piR-12487**	Czech	77	23	70	5	7	18
**Neuroblastoma**	Wang, H 2023 [57]	**hsa-piR-1089**	China	38	3	NA	NA	NA	NA
**Lung Ca**	Li, J 2021 [58]	**piR-26925**	China	70	57	58	20	12	37
		**piR-5444**	China	70	57	49	19	21	38
	Li, Y 2022 [59]	**piR164586**	China	115	47	60	19	55	28
**Prostate Ca**	Markert, L 2021 [60]	**piRNA-018849**	Germany	28	25	NA	NA	NA	NA
		**piRNA-019324**	Germany	28	25	NA	NA	NA	NA
**Renal Cell Ca**	Iliev, R 2016 [25]	**piR-823**	Czech	178	101	NA	NA	NA	NA
**Thyroid Ca**	Chang, Z 2020 [61]	**piR-13643**	China	75	31	NA	NA	NA	NA
		**piR-21238**	China	75	31	NA	NA	NA	NA

Ca: cancer; TP: true positive; FP: false positive; FN: false negative; TN: true negative

## Results

**Fig 1:**
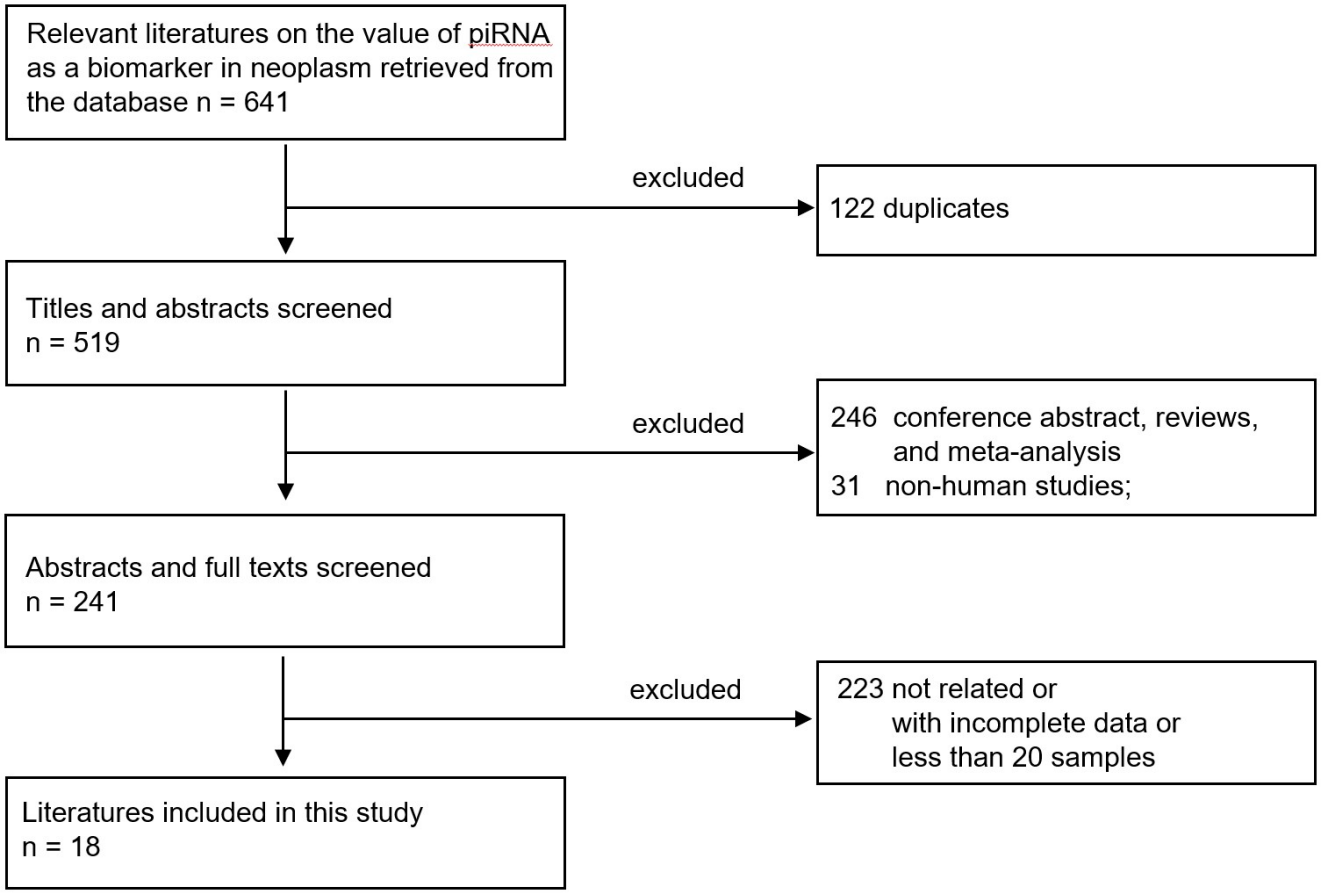
Flow chart of the research process.

We searched three databases (PubMed, Embase, and Cochrane) for relevant literature and identified 641 papers. After removing duplicates (n = 122), we screened titles and abstracts and removed conference abstracts, reviews, meta-analyses, and non-human studies (n = 277). Full texts and abstracts were examined, and manuscripts unrelated to the subject and with incomplete data or fewer than 20 samples were removed (n = 233). Finally, 18 studies with 2,352 patients were included in this systematic review (Fig 1, Table 1, S4 Table). The most frequently studied type of cancer was colorectal cancer (n = 7, number of patients = 1,488).

### Comparison of diagnostic performance of piRNAs in colorectal cancer

**Fig 2:**
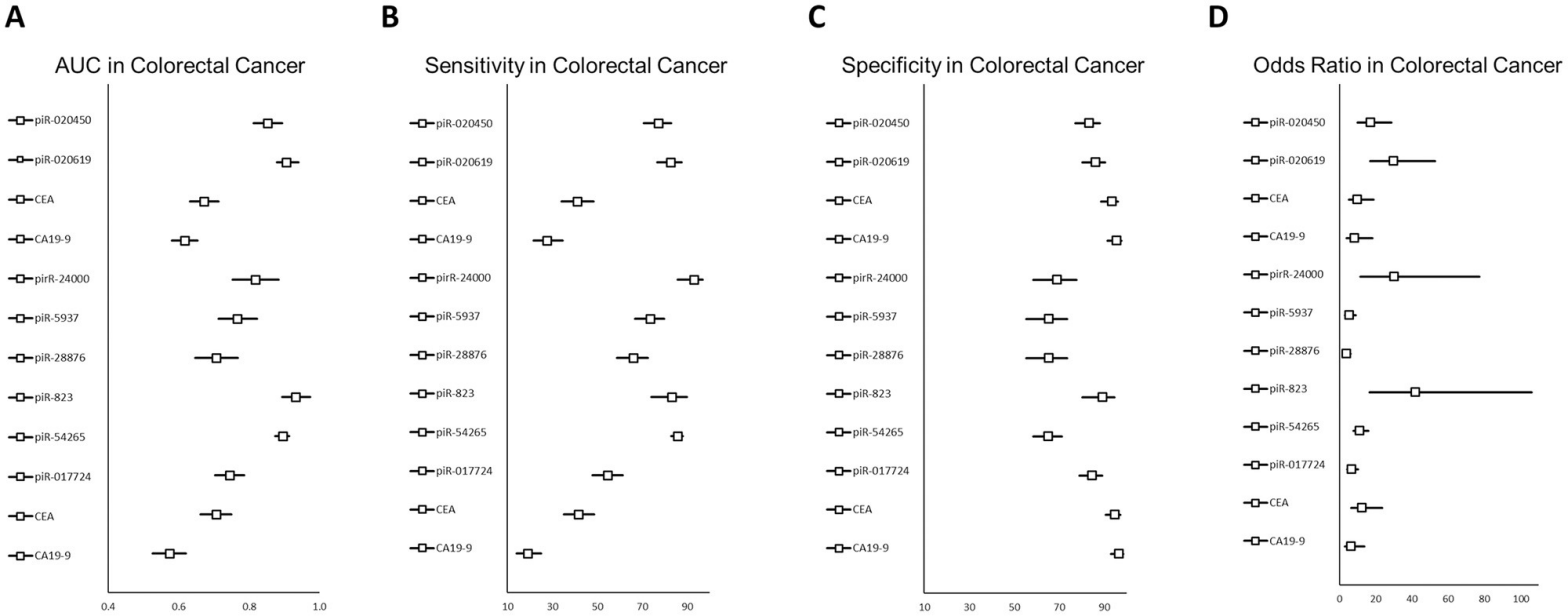
Comparison of the diagnostic performance of piRNAs in colorectal cancer. Representative values of diagnostic performance including AUC value (A), sensitivity (B), specificity (C) and odds ratio (D) for colorectal cancer were compared.

The AUC values of 9 piRNAs were compared in patients with colorectal cancer (n = 1,488, Fig 2A). Their AUC values ranged from 0.707 to 0.942; however, the current clinical diagnostic biomarkers (carcinoembryonic antigen (CEA) and cancer antigen 19–9 (CA 19–9)) for colorectal cancer ranged from 0.672 to 0.707 and 0.574 to 0.617, respectively. piR-31143 had the highest value (0.942), whereas piR-28876 had the lowest value (0.707). Most piRNAs in these studies showed better AUC values than current biomarkers.

The sensitivities of the 9 piRNAs in colorectal cancer patients had values from 54.60 to 93.10%; however, current clinical diagnostic biomarkers (CEA and CA19-9) for colorectal cancer had respective values from 41.11 to 41.80 and from 19.10 to 27.78 (Fig 2B). piR-24000 showed the highest sensitivity (93.10) and piR-017724 showed the lowest sensitivity (54.6). Most piRNAs in these studies showed better sensitivity than the current biomarkers.

Specificities of the 9 piRNA in colorectal cancer were from 65.10 to 100, while current clinical diagnostic biomarkers (CEA and CA19-9) for this cancer range from 93.33 to 94.6 and from 95.56 to 96.4, respectively (Fig 2C). piR-31143 showed the highest specificity (100) and piR-54265 showed the lowest sensitivity (65.10). Overall, most piRNAs in these studies showed poorer specificity than the current biomarkers.

Odds ratios of 8 piRNAs in patients with colorectal cancer were from 3.59 to 41.88, and current clinical diagnostic biomarkers (CEA and CA19-9) are characterized by values from 9.77 to 12.46 and from 6.25 to 8.27, respectively (Fig 2D). piR-823 showed the highest odds ratio (41.88) and piR-28876 showed the lowest odds ratio (3.59).

Generally, the piRNAs in this review showed better sensitivity and AUC values than the current clinical diagnostic biomarkers for CRC, although the current biomarkers showed better specificity.

### Comparison of diagnostic performance of piRNAs in other cancers

**Fig 3:**
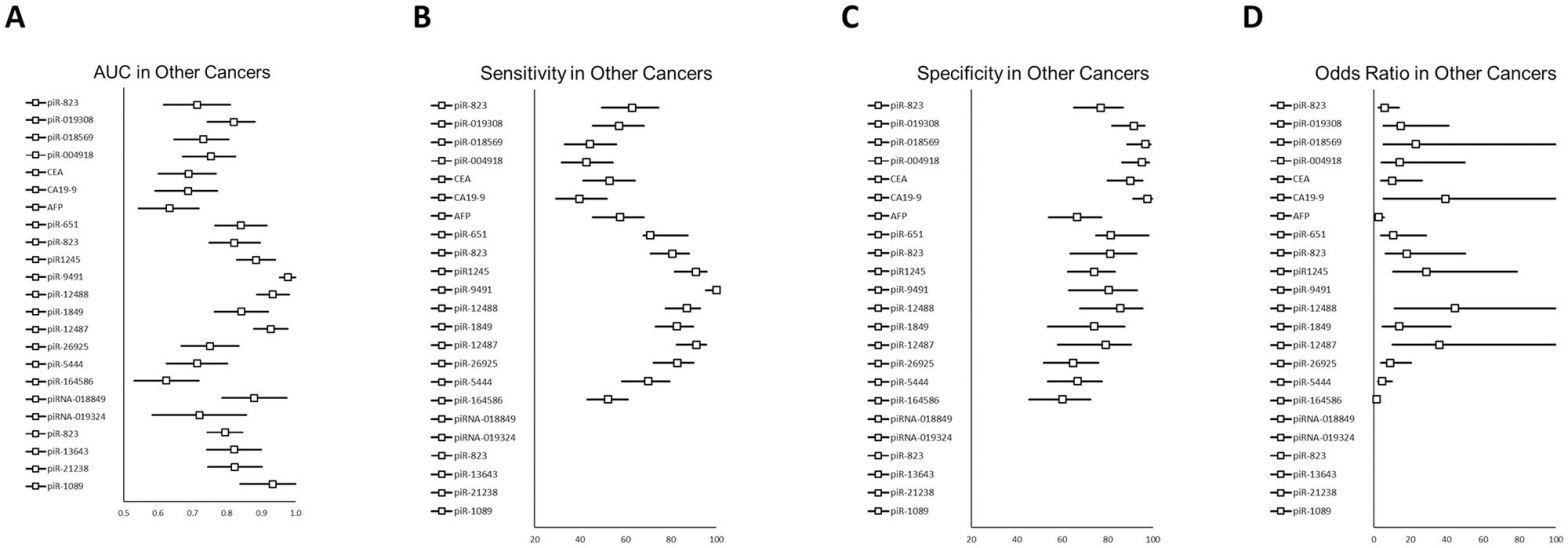
Comparison of the diagnostic performance of piRNAs in a variety of cancer types, excluding colorectal cancer. Representative values of diagnostic performance including AUC value (A), sensitivity (B), specificity (C) and odds ratio (D) for colorectal cancer were compared.

The AUC values of 20 piRNAs were compared in a variety of cancer patients (n = 864, Fig 3A), except for those with colorectal cancer. The AUC ranged from 0.624 to 0.978. piR-9491 had the highest value (0.978), whereas piR-164568 had the lowest value (0.624).

The sensitivities of 14 piRNAs were compared in a variety of cancer patients (n = 583, Fig 3B), except for those with colorectal cancer. The sensitivities were between 42.86 to 100. piR-9491 showed the highest sensitivity (100), whereas piR-004918 showed the lowest sensitivity (42.86).

The specificities of 14 piRNAs were compared in a variety of cancer patients (n = 583, Fig 3C), except for those with colorectal cancer. Their specificities ranged from 60.10 to 96.67. piR-018569 showed the highest specificity (96.67), whereas piR-164568 showed the lowest specificity (60.10).

Odds ratios (ORs) of 13 piRNAs were compared in a variety of cancer patients (n = 583, Fig 3D), except for those with colorectal cancer. Their odds ratios were between 1.61 and 44.67. piR-12488 showed the highest odds ratio (44.67), and piR-164568 showed the lowest value (1.61).

## Discussion

In this review, we examined the diagnostic value of piRNAs in a variety of cancers. Interestingly, their usefulness as serum biomarkers has been examined most frequently in patients with CRC. We found that piRNAs performed better than current serum biomarkers, including CEA, CA19-9 and guanylyl cyclase C (GCC). Moreover, piR-823 showed a good diagnostic performance in several cancer types.

piR-823 showed better performance among reviewed piRNAs in colorectal cancer (Fig 2). The average odds ratio of reviewed biomarkers in colorectal cancer was 15.16. The best piRNA in odds ratio was piR-823(41.88, 95% confidence interval (CI): 16.51~106.24), followed by piR-24000 (30, 95% CI: 11.66~77.22) and piR-020619 (29.8, 95% CI:16.81~52.84). The average AUC value was 0.78. The best piRNA was piR-31143 (0.94, 95% CI: 0.75~1.00), followed by piR-823 (0.93, 95% CI: 0.89~0.97) and piR-020619 (0.91, 95% CI: 0.88~0.94). The average sensitivity value was 63.31. The best piRNA was piR-24000 (93.10, 95% CI: 85.76~96.80), followed by piR-54265 (85.70, 95% CI: 82.92~88.02) and piR-823 (83.30, 95% CI: 73.95~89.80). The average specificity value was 83.68. The best piRNA was piR-31143 (100), followed by piR-823 (89.3, 95% CI 80.34~94.50) and piR-020619 (86.11, 95% CI: 80.30~90.41). In other types of cancer including gastric cancer, piR-823 showed better diagnostic performance (Fig 3).

Several serum biomarkers are available for the detection and monitoring of colorectal cancer. CEA is a glycoprotein associated with cellular adhesion [30]. Although it is normally expressed during fetal development, it is overexpressed in many cancers, including colorectal cancer. Elevated levels of CEA in serum have been found to be associated with advanced colorectal cancer and poor prognosis [31]. However, due to its poor sensitivity and prevalence in non-malignant conditions, the use of this marker is not recommended in colorectal cancer. CA 19–9 is a carbohydrate antigen normally synthesized in the gastrointestinal tract [30]. However, its expression is elevated in several gastrointestinal cancers including colorectal cancer. Nonetheless, its use as a biomarker for colorectal cancer is limited because of its low sensitivity and specificity. GCC is a transmembrane receptor expressed in normal colorectal tissues and overexpressed in colorectal cancer [32]. Serum GCC levels are elevated in patients with colorectal cancer and may be useful as a biomarker for early detection. miRNAs are small non-coding RNA molecules involved in the regulation of gene expression [33]. Several studies have identified specific miRNAs that are dysregulated in colorectal cancer and may serve as potential serum biomarkers. As current serum biomarkers show better specificity and poorer sensitivity (Fig 2), their combination with piRNAs could improve the overall diagnostic performance.

piR-823 which consists of 28 nucleotides was first reported in gastric cancer by Cui et al. [21]. Its expression was noted in white blood cells [21], cancer cell lines and blood plasma [26, 34]. Its association with malignancy has been reported in many cancer types including breast [35], colorectal, gastric [21], prostate cancer [36], esophageal cancer [37], liver cancer [38] and multiple myeloma [39]. Although the expressional regulation of its target genes such as DNMT1, DNMT3A and DNMT3B has been reported in breast cancer cells [35], one of its notable underlying mechanisms for the progression of malignancy is its binding with its target proteins and regulation of their activity. In colorectal cancer cells, several interaction partners had been studied. It interacts with HSF1 and regulates the phosphorylation at Ser326 and then the transcriptional activity in colorectal cancer cells [40]. Its interaction with HSF1 finally led to the increased expression of HSP27, HSP60 and HSP70. Another interaction partner is PINK1 and its interaction with PINK1 promoted the proteasome-mediated degradation of PINK1, which leads to the inhibition of mitophagy and apoptosis of colorectal cancer cells [41]. In multiple myeloma, it interacts with DNMT3B and leads to the aberrant DNA methylation [39]. In liver fibrogenesis, it interacts with EIF3B and increased the translation of TGF-β1 mRNA [42].

In addition to its oncogenic roles, some studies have suggested that piR-823 may act as a tumor suppressor. The expression level of piR-823 was decreased in the tumor tissue of renal cell carcinoma but positively correlated with worse outcomes [25]. In addition, its expression level is significantly lower in gastric cancer tissues than in non-cancerous tissues [43]. Moreover, piR-823 blood levels are lower in patients with gastric cancer than in controls, even though its presence is positively associated with tumor-node-metastasis and distant metastasis [21].

The role of piR-823 as a prognostic factor has been reported in multiple myeloma, where it re-educates endothelial cells in the tumor microenvironment [44]. PIWI proteins have been associated with a poor prognosis in glioblastoma [45], colorectal cancer [46] and liver cancer [47].

The study design of all 18 articles included in this review were a case-control study (S4 Table). To establish those piRNA as a clinical biomarker, further prospective studies are required [48]. Samples need to be collected before cancer development or diagnosis and patients who develop cancer during subsequent follow-up need to be compared with control patients matched for confounding variables.

### Conclusion & limitation

This study showed that the piRNAs can be novel clinical biomarkers for the cancer diagnosis because they showed better sensitivity and AUC values than current clinical diagnostic biomarkers, although current biomarkers appear to be more specific. In addition, piR-823 showed a significant diagnostic performance in four types of cancer (colorectal, esophageal, gastric, and renal cell cancer). Further prospective studies are required for their validation because all 18 studies in this review were a case-control study. Cautious interpretation is required because there is heterogeneity in aspects of cancer type, population, materials and experimental methods (Table 1 and S4 Table) in this review. In addition, pre-prints or grey literature were not included in this review.

## Supporting information

S1 Table PRISMA checklist. (PDF)

S2 Table AMSTAR checklist. (PDF)

S3 Table QUADAS-2 checklist. (PDF)

S4 Table The study characteristics of included articles. (PDF)

S1 File Total references. (PDF)
