# Supplementary material for: The potential of piR-823 as a diagnostic biomarker in oncology: A systematic review
Source: PLoS One. 2023 Dec 7;18(12):e0294685. doi: 10.1371/journal.pone.0294685 (PMC10703285; doi:10.1371/journal.pone.0294685)
Supplement: S3 Table — (PDF) [file pone.0294685.s003.pdf]

Supporting Information 3. QUADAS-2 Checklist

	Risk of Bias				Applicability Concerns		
Included Articles	Patient Selection	Index Test	Reference Standard	Flow and Timing	Patient Selection	Index Test	Reference Standard
Bartos, M 2021	U	L	L	L	U	L	L
Chang, Z 2020	L	H	L	L	L	H	L
Cui, L 2011	U	L	L	L	U	L	L
Ge, L 2020	H	L	L	L	H	L	L
Iliev, R 2016	L	H	L	L	L	H	L
Iyer, DN 2020	H	L	L	L	H	L	L
Li, J 2021	L	L	L	L	L	L	L
Li, J 2022	H	U	U	U	H	U	U
Li, Y 2022	L	L	L	L	L	L	L
Mai, D 2020	L	L	L	L	L	L	L
Markert, L 2021	L	H	L	L	L	H	L
Qu, A 2019	L	L	L	L	L	L	L
Sabbah, NA 2021	L	L	L	L	L	L	L
Su, JF 2020	L	L	L	L	L	L	L
Vychytilova-Faltejskova, P 2018	L	L	L	L	L	L	L
Wang, H 2023	L	H	L	L	L	H	L
Wang, Z 2020	L	L	L	L	L	L	L
Zhou, X 2020	U	L	L	L	U	L	L

*H : High Risk, L : Low Risk, U : Unclear Risk
