# Supplementary material for: The potential of piR-823 as a diagnostic biomarker in oncology: A systematic review
Source: PLoS One. 2023 Dec 7;18(12):e0294685. doi: 10.1371/journal.pone.0294685 (PMC10703285; doi:10.1371/journal.pone.0294685)
Supplement: S4 Table — (PDF) [file pone.0294685.s004.pdf]

Supporting Information 4. The study characteristics of included articles

Cancer Type	Study ID (Ref)	Type of Samples	Quantification	Settings	Timing of Evaluation	Age	Gender	Other Types of Cancers	Sample Size per Cancer Type
**Colorectal Ca**	Wang, Z 2020	serum	RNA-Seq/qPCR	cancer vs healthy	Preop	61.5±11	M:98, F:82	other cancer-free patients only	212.6
	Iyer, DN 2020	tissue	RNA-Seq	cancer vs adjacent normal	Preop	70(29~97)	M:51, F36	NA	
	Vychytilova-Faltejskova, P 2018	serum	RNA-Seq	cancer vs healthy	NA(Periop)*	66±12	M:94, F:85	NA	
	Sabbah, NA 2021	serum	qPCR	cancer vs healthy	NA(Periop)	59.12±2.61	M:47, F:37	other disease-free patients only	
	Mai, D 2020	serum	digital PCR	cancer vs healthy	NA(Periop)	58(19~83)	M:456, F:269	other cancer-free patients only	
	Qu, A 2019	serum	qPCR	cancer vs healthy	Preop	61(39~73)	M:127, F:93	other cancer-free patients only	
	Li, J 2022	serum	qPCR	cancer vs healthy	NA(Periop)	NA	NA	NA	
**Esophageal Ca**	Su, JF 2020	tissue	qPCR	cancer vs adjacent normal	Preop	≤60:10, ≥61:44	M: 30, F 24	NA	54.0
**Gastric Ca**	Ge, L 2020	serum	RNA-Seq	cancer vs heathly	Preop	59.4±11.2	M:40, F:30	other-disease-free patients only	76.3
	Cui, L 2011	blood	qPCR	cancer vs healthy	Pre: 42, Postop:51	61.5±14.8	M: 77, F: 48	NA	
	Zhou, X 2020	gastric juice	qPCR	cacner vs healthy	NA(Periop)	<60:29, ≥60:37	M:36, F:30	NA	
**Glioblastoma**	Bartos, M 2021	tissue	RNA-Seq	cacner vs epilepsy patients	Preop	64(30~80)	M:41, F:36	NA	77.0
**Neuroblastoma**	Wang, H 2023	plasma	qPCR	cancer vs healthy	NA(Periop)	≥18:25, <8:13	M:16, F:22	NA	38.0
**Lung Ca**	Li, J 2021	serum	qPCR	cancer vs healthy	NA(Periop)	≤60:42, >60:28	M:30, F:40	NA	92.5
	Li, Y 2022	serum	qPCR	cancer vs healthy	NA(Periop)	<55:35, 56-65:48, >66:32	M:28, F:87	other disease-free patients only	
**Prostate Ca**	Markert, L 2021	urine	qPCR	cancer vs healthy	NA(Periop)	69.5(44~84)	NA	infectious & related disease patients excluded	28.0
**Renal Cell Ca**	Iliev, R 2016	serum	qPCR	cancer vs healthy	NA(Periop)	21~84(med 64)	NA	NA	178.0
**Thyroid Ca**	Chang, Z 2020	tissue	RNA-Seq	cancer vs healthy	NA(Periop)	<45:39, ≥45:36	M:22, F53	other cancer-free patients only	75.0

* not clearly explained. However the tests were presumed to be done in the perioperative period based on the main text.
