# Supplementary material for: The potential of piR-823 as a diagnostic biomarker in oncology: A systematic review
Source: PLoS One. 2023 Dec 7;18(12):e0294685. doi: 10.1371/journal.pone.0294685 (PMC10703285; doi:10.1371/journal.pone.0294685)
Supplement: S1 File — (PDF) [file pone.0294685.s005.pdf]

Total References (n=641)

1. Abell NS, Mercado M, Cañeque T, Rodriguez R, Xhemalce B. Click Quantitative Mass Spectrometry Identifies PIWIL3 as a Mechanistic Target of RNA Interference Activator Enoxacin in Cancer Cells. J Am Chem Soc. 2017;139(4):1400-3. Epub 20170123. doi: 10.1021/jacs.6b11751. PubMed PMID: 28094937.

2. Abramowicz A, Story MD. The Long and Short of It: The Emerging Roles of Non-Coding RNA in Small Extracellular Vesicles. Cancers (Basel). 2020;12(6). Epub 20200602. doi: 10.3390/cancers12061445. PubMed PMID: 32498257; PubMed Central PMCID: PMCPMC7352322.

3. Adashev VE, Kotov AA, Bazylev SS, Shatskikh AS, Aravin AA, Olenina LV. Stellate Genes and the piRNA Pathway in Speciation and Reproductive Isolation of Drosophila melanogaster. Front Genet. 2020;11:610665. Epub 20210122. doi: 10.3389/fgene.2020.610665. PubMed PMID: 33584811; PubMed Central PMCID: PMCPMC7874207.

4. Alahari SV, Eastlack SC, Alahari SK. Role of Long Noncoding RNAs in Neoplasia: Special Emphasis on Prostate Cancer. Int Rev Cell Mol Biol. 2016;324:229-54. Epub 20160302. doi: 10.1016/bs.ircmb.2016.01.004. PubMed PMID: 27017010.

5. Amaar YG, Reeves ME. RASSF1C regulates miR-33a and EMT marker gene expression in lung cancer cells. Oncotarget. 2019;10(2):123-32. Epub 20190104. doi: 10.18632/oncotarget.26498. PubMed PMID: 30719208; PubMed Central PMCID: PMCPMC6349430.

6. Ameli Mojarad M, Ameli Mojarad M, Shojaee B, Nazemalhosseini-Mojarad E. piRNA: A promising biomarker in early detection of gastrointestinal cancer. Pathol Res Pract. 2022;230:153757. Epub 20211230. doi: 10.1016/j.prp.2021.153757. PubMed PMID: 34998210.

7. AmeliMojarad M, Amelimojarad M. piRNAs and PIWI proteins as potential biomarkers in Breast cancer. Mol Biol Rep. 2022;49(10):9855-62. Epub 20220525. doi: 10.1007/s11033-022-07506-x. PubMed PMID: 35612777.

8. Andreev VI, Yu C, Wang J, Schnabl J, Tirian L, Gehre M, et al. Panoramix SUMOylation on chromatin connects the piRNA pathway to the cellular heterochromatin machinery. Nat Struct Mol Biol. 2022;29(2):130-42. Epub 20220216. doi: 10.1038/s41594-022-00721-x. PubMed PMID: 35173350.

9. Armisen J, Gilchrist MJ, Wilczynska A, Standart N, Miska EA. Abundant and dynamically expressed miRNAs, piRNAs, and other small RNAs in the vertebrate Xenopus tropicalis. Genome Res. 2009;19(10):1766-75. Epub 20090723. doi: 10.1101/gr.093054.109. PubMed PMID: 19628731; PubMed Central PMCID: PMCPMC2765267.

10. Ashe A, Sapetschnig A, Weick EM, Mitchell J, Bagijn MP, Cording AC, et al. piRNAs can trigger a multigenerational epigenetic memory in the germline of C. elegans. Cell. 2012;150(1):88-99. Epub 20120625. doi: 10.1016/j.cell.2012.06.018. PubMed PMID: 22738725; PubMed Central PMCID: PMCPMC3464430.

11. Assumpção CB, Calcagno DQ, Araújo TM, Santos SE, Santos Â K, Riggins GJ, et al. The role of piRNA and its potential clinical implications in cancer. Epigenomics. 2015;7(6):975-84. Epub 20150501. doi: 10.2217/epi.15.37. PubMed PMID: 25929784; PubMed Central PMCID: PMCPMC4750480.

12. Bagijn MP, Goldstein LD, Sapetschnig A, Weick EM, Bouasker S, Lehrbach NJ, et al. Function, targets, and evolution of Caenorhabditis elegans piRNAs. Science. 2012;337(6094):574-8. Epub 20120614. doi: 10.1126/science.1220952. PubMed PMID: 22700655; PubMed Central PMCID: PMCPMC3951736.

13. Bahn JH, Zhang Q, Li F, Chan TM, Lin X, Kim Y, et al. The landscape of microRNA, Piwi-interacting RNA, and circular RNA in human saliva. Clin Chem. 2015;61(1):221-30. Epub 20141106. doi: 10.1373/clinchem.2014.230433. PubMed PMID: 25376581; PubMed Central PMCID: PMCPMC4332885.

14. Bajo-Santos C, Brokāne A, Zayakin P, Endzeliņš E, Soboļevska K, Belovs A, et al. Plasma and urinary extracellular vesicles as a source of RNA biomarkers for prostate cancer in liquid biopsies. Front Mol Biosci. 2023;10:980433. Epub 20230203. doi: 10.3389/fmolb.2023.980433. PubMed PMID: 36818049; PubMed Central PMCID: PMCPMC9935579.

15. Balaratnam S, West N, Basu S. A piRNA utilizes HILI and HIWI2 mediated pathway to down-regulate ferritin heavy chain 1 mRNA in human somatic cells. Nucleic Acids Res. 2018;46(20):10635-48. doi: 10.1093/nar/gky728. PubMed PMID: 30102404; PubMed Central PMCID: PMCPMC6237762.

16. Balatti V, Nigita G, Veneziano D, Drusco A, Stein GS, Messier TL, et al. tsRNA signatures in cancer. Proc Natl Acad Sci U S A. 2017;114(30):8071-6. Epub 20170710. doi: 10.1073/pnas.1706908114. PubMed PMID: 28696308; PubMed Central PMCID: PMCPMC5544330.

17. Balatti V, Pekarsky Y, Croce CM. Role of the tRNA-Derived Small RNAs in Cancer: New Potential Biomarkers and Target for Therapy. Adv Cancer Res. 2017;135:173-87. Epub 20170810. doi: 10.1016/bs.acr.2017.06.007. PubMed PMID: 28882222.

18. Barberán-Soler S, Fontrodona L, Ribó A, Lamm AT, Iannone C, Cerón J, et al. Co-option of the piRNA pathway for germline-specific alternative splicing of C. elegans TOR. Cell Rep. 2014;8(6):1609-16. Epub 20140915. doi: 10.1016/j.celrep.2014.08.016. PubMed PMID: 25220461.

19. Bartos M, Siegl F, Kopkova A, Radova L, Oppelt J, Vecera M, et al. Small RNA Sequencing Identifies PIWI-Interacting RNAs Deregulated in Glioblastoma-piR-9491 and piR-12488 Reduce Tumor Cell Colonies In Vitro. Front Oncol. 2021;11:707017. Epub 20210813. doi: 10.3389/fonc.2021.707017. PubMed PMID: 34485142; PubMed Central PMCID: PMCPMC8415021.

20. Batki J, Schnabl J, Wang J, Handler D, Andreev VI, Stieger CE, et al. The nascent RNA binding complex SFiNX licenses piRNA-guided heterochromatin formation. Nat Struct Mol Biol. 2019;26(8):720-31. Epub 20190805. doi: 10.1038/s41594-019-0270-6. PubMed PMID: 31384064; PubMed Central PMCID: PMCPMC6828549.

21. Beg A, Parveen R, Fouad H, Yahia ME, Hassanein AS. Role of different non-coding RNAs as ovarian cancer biomarkers. J Ovarian Res. 2022;15(1):72. Epub 20220617. doi: 10.1186/s13048-022-01002-3. PubMed PMID: 35715825; PubMed Central PMCID: PMCPMC9206245.

22. Bezler A, Braukmann F, West SM, Duplan A, Conconi R, Schütz F, et al. Tissue- and sex-specific small RNAomes reveal sex differences in response to the environment. PLoS Genet. 2019;15(2):e1007905. Epub 20190208. doi: 10.1371/journal.pgen.1007905. PubMed PMID:

30735500; PubMed Central PMCID: PMCPMC6383947.

23. Bian H, Zhou Y, Zhou D, Zhang Y, Shang D, Qi J. The latest progress on miR-374 and its functional implications in physiological and pathological processes. J Cell Mol Med. 2019;23(5):3063-76. Epub 20190217. doi: 10.1111/jcmm.14219. PubMed PMID: 30772950; PubMed Central PMCID: PMCPMC6484333.

24. Biscotti MA, Canapa A, Forkoni M, Gerdol M, Pallavicini A, Schartl M, et al. The small non-coding RNA processing machinery of two living fossil species, lungfish and coelacanth, gives new insights into the evolution of the Argonaute protein family. Genome Biol Evol. 2017;9(3):438-53. Epub 20170216. doi: 10.1093/gbe/evx017. PubMed PMID: 28206606; PubMed Central PMCID: PMCPMC5381642.

25. Bornelöv S, Czech B, Hannon GJ. An evolutionarily conserved stop codon enrichment at the 5' ends of mammalian piRNAs. Nat Commun. 2022;13(1):2118. Epub 20220419. doi: 10.1038/s41467-022-29787-3. PubMed PMID: 35440552; PubMed Central PMCID: PMCPMC9018710.

26. Brock M, Mei Y. Protein functional effector sncRNAs (pfeRNAs) in lung cancer. Cancer Lett. 2017;403:138-43. Epub 20170619. doi: 10.1016/j.canlet.2017.06.013. PubMed PMID: 28642173.

27. Busch J, Ralla B, Jung M, Wotschofsky Z, Trujillo-Arribas E, Schwabe P, et al. Piwi-interacting RNAs as novel prognostic markers in clear cell renal cell carcinomas. J Exp Clin Cancer Res. 2015;34(1):61. Epub 20150614. doi: 10.1186/s13046-015-0180-3. PubMed PMID: 26071182; PubMed Central PMCID: PMCPMC4467205.

28. Cai A, Hu Y, Zhou Z, Qi Q, Wu Y, Dong P, et al. PIWI-Interacting RNAs (piRNAs): Promising Applications as Emerging Biomarkers for Digestive System Cancer. Front Mol Biosci. 2022;9:848105. Epub 20220127. doi: 10.3389/fmolb.2022.848105. PubMed PMID: 35155584; PubMed Central PMCID: PMCPMC8829394.

29. Casarotto M, Fanetti G, Guerrieri R, Palazzari E, Lupato V, Steffan A, et al. Beyond MicroRNAs: Emerging Role of Other Non-Coding RNAs in HPV-Driven Cancers. Cancers (Basel). 2020;12(5). Epub 20200515. doi: 10.3390/cancers12051246. PubMed PMID: 32429207; PubMed Central PMCID: PMCPMC7281476.

30. Castro-Oropeza R, Piña-Sánchez P. Epigenetic and Transcriptomic Regulation Landscape in HPV+ Cancers: Biological and Clinical Implications. Front Genet. 2022;13:886613. Epub 20220614. doi: 10.3389/fgene.2022.886613. PubMed PMID: 35774512; PubMed Central PMCID: PMCPMC9237502.

31. Chalbatani GM, Dana H, Memari F, Gharagozlou E, Ashjaei S, Kheirandish P, et al. Biological function and molecular mechanism of piRNA in cancer. Pract Lab Med. 2019;13:e00113. Epub 20181207. doi: 10.1016/j.plabm.2018.e00113. PubMed PMID: 30705933; PubMed Central PMCID: PMCPMC6349561.

32. Chang Z, Ji G, Huang R, Chen H, Gao Y, Wang W, et al. PIWI-interacting RNAs piR-13643 and piR-21238 are promising diagnostic biomarkers of papillary thyroid carcinoma. Aging (Albany NY). 2020;12(10):9292-310. Epub 20200519. doi: 10.18632/aging.103206. PubMed PMID: 32428871; PubMed Central PMCID: PMCPMC7288952.

33. Chavda V, Madhwani K, Chaurasia B. PiWi RNA in Neurodevelopment and Neurodegenerative Disorders. Curr Mol Pharmacol. 2022;15(3):517-31. doi:

10.2174/1874467214666210629164535. PubMed PMID: 34212832.

34. Chen CC, Qian X, Yoon BJ. Effective computational detection of piRNAs using n-gram models and support vector machine. BMC Bioinformatics. 2017;18(Suppl 14):517. Epub 20171228. doi: 10.1186/s12859-017-1896-1. PubMed PMID: 29297285; PubMed Central PMCID: PMCPMC5751586.

35. Chen H, Zhuang Z, Chen Y, Qiu C, Qin Y, Tan C, et al. A universal platform for one-pot detection of circulating non-coding RNA combining CRISPR-Cas12a and branched rolling circle amplification. Anal Chim Acta. 2023;1246:340896. Epub 20230125. doi: 10.1016/j.aca.2023.340896. PubMed PMID: 36764778.

36. Chen L, Chen Z, Simões A, Wu X, Dai Y, DiPietro LA, et al. Site-Specific Expression Pattern of PIWI-Interacting RNA in Skin and Oral Mucosal Wound Healing. Int J Mol Sci. 2020;21(2). Epub 20200114. doi: 10.3390/ijms21020521. PubMed PMID: 31947648; PubMed Central PMCID: PMCPMC7013508.

37. Chen S, Ben S, Xin J, Li S, Zheng R, Wang H, et al. The biogenesis and biological function of PIWI-interacting RNA in cancer. J Hematol Oncol. 2021;14(1):93. Epub 20210612. doi: 10.1186/s13045-021-01104-3. PubMed PMID: 34118972; PubMed Central PMCID: PMCPMC8199808.

38. Chen W, Li L, Wang J, Li Q, Zhang R, Wang S, et al. Extracellular vesicle YRNA in atherosclerosis. Clin Chim Acta. 2021;517:15-22. Epub 20210218. doi: 10.1016/j.cca.2021.02.003. PubMed PMID: 33609558.

39. Chen YA, Stuwe E, Luo Y, Ninova M, Le Thomas A, Rozhavskaya E, et al. Cutoff Suppresses RNA Polymerase II Termination to Ensure Expression of piRNA Precursors. Mol Cell. 2016;63(1):97-109. Epub 20160609. doi: 10.1016/j.molcel.2016.05.010. PubMed PMID: 27292797; PubMed Central PMCID: PMCPMC4980073.

40. Cheng Y, Wang Q, Jiang W, Bian Y, Zhou Y, Gou A, et al. Emerging roles of piRNAs in cancer: challenges and prospects. Aging (Albany NY). 2019;11(21):9932-46. Epub 20191113. doi: 10.18632/aging.102417. PubMed PMID: 31727866; PubMed Central PMCID: PMCPMC6874451.

41. Chettimada S, Lorenz DR, Misra V, Wolinsky SM, Gabuzda D. Small RNA sequencing of extracellular vesicles identifies circulating miRNAs related to inflammation and oxidative stress in HIV patients. BMC Immunol. 2020;21(1):57. Epub 20201111. doi: 10.1186/s12865-020-00386-5. PubMed PMID: 33176710; PubMed Central PMCID: PMCPMC7656686.

42. Chu H, Hui G, Yuan L, Shi D, Wang Y, Du M, et al. Identification of novel piRNAs in bladder cancer. Cancer Lett. 2015;356(2 Pt B):561-7. Epub 20141008. doi: 10.1016/j.canlet.2014.10.004. PubMed PMID: 25305452.

43. Chu H, Xia L, Qiu X, Gu D, Zhu L, Jin J, et al. Genetic variants in noncoding PIWI-interacting RNA and colorectal cancer risk. Cancer. 2015;121(12):2044-52. Epub 20150304. doi: 10.1002/cncr.29314. PubMed PMID: 25740697.

44. Chung IF, Chang SJ, Chen CY, Liu SH, Li CY, Chan CH, et al. YM500v3: a database for small RNA sequencing in human cancer research. Nucleic Acids Res. 2017;45(D1):D925-d31. Epub 20161129. doi: 10.1093/nar/gkw1084. PubMed PMID: 27899625; PubMed Central PMCID: PMCPMC5210564.

45. Cook MS, Blelloch R. Small RNAs in germline development. Curr Top Dev Biol. 2013;102:159-

205. doi: 10.1016/b978-0-12-416024-8.00006-4. PubMed PMID: 23287033.

46. Cordeiro A, Monzó M, Navarro A. Non-Coding RNAs in Hodgkin Lymphoma. Int J Mol Sci. 2017;18(6). Epub 20170529. doi: 10.3390/ijms18061154. PubMed PMID: 28555062; PubMed Central PMCID: PMCPMC5485978.

47. Costa AL, Lobo J, Jerónimo C, Henrique R. The epigenetics of testicular germ cell tumors: looking for novel disease biomarkers. Epigenomics. 2017;9(2):155-69. Epub 20170118. doi: 10.2217/epi-2016-0081. PubMed PMID: 28097877.

48. Cui L, Lou Y, Zhang X, Zhou H, Deng H, Song H, et al. Detection of circulating tumor cells in peripheral blood from patients with gastric cancer using piRNAs as markers. Clin Biochem. 2011;44(13):1050-7. Epub 20110617. doi: 10.1016/j.clinbiochem.2011.06.004. PubMed PMID: 21704610.

49. Dabi Y, Bendifallah S, Suisse S, Haury J, Touboul C, Puchar A, et al. Overview of non-coding RNAs in breast cancers. Transl Oncol. 2022;25:101512. Epub 20220809. doi: 10.1016/j.tranon.2022.101512. PubMed PMID: 35961269; PubMed Central PMCID: PMCPMC9382556.

50. Danielson KM, Rubio R, Abderazzaq F, Das S, Wang YE. High Throughput Sequencing of Extracellular RNA from Human Plasma. PLoS One. 2017;12(1):e0164644. Epub 20170106. doi: 10.1371/journal.pone.0164644. PubMed PMID: 28060806; PubMed Central PMCID: PMCPMC5218574.

51. Das B, Jain N, Mallick B. piR-39980 mediates doxorubicin resistance in fibrosarcoma by regulating drug accumulation and DNA repair. Commun Biol. 2021;4(1):1312. Epub 20211119. doi: 10.1038/s42003-021-02844-1. PubMed PMID: 34799689; PubMed Central PMCID: PMCPMC8605029.

52. Das B, Roy J, Jain N, Mallick B. Tumor suppressive activity of PIWI-interacting RNA in human fibrosarcoma mediated through repression of RRM2. Mol Carcinog. 2019;58(3):344-57. Epub 20181125. doi: 10.1002/mc.22932. PubMed PMID: 30362638.

53. Das PP, Bagijn MP, Goldstein LD, Woolford JR, Lehrbach NJ, Sapetschnig A, et al. Piwi and piRNAs act upstream of an endogenous siRNA pathway to suppress Tc3 transposon mobility in the Caenorhabditis elegans germline. Mol Cell. 2008;31(1):79-90. Epub 20080619. doi: 10.1016/j.molcel.2008.06.003. PubMed PMID: 18571451; PubMed Central PMCID: PMCPMC3353317.

54. Daugaard I, Venø MT, Yan Y, Kjeldsen TE, Lamy P, Hager H, et al. Small RNA sequencing reveals metastasis-related microRNAs in lung adenocarcinoma. Oncotarget. 2017;8(16):27047-61. doi: 10.18632/oncotarget.15968. PubMed PMID: 28460486; PubMed Central PMCID: PMCPMC5432317.

55. de Mooij T, Peterson TE, Evans J, McCutcheon B, Parney IF. Short non-coding RNA sequencing of glioblastoma extracellular vesicles. J Neurooncol. 2020;146(2):253-63. Epub 20200107. doi: 10.1007/s11060-019-03384-9. PubMed PMID: 31912278.

56. Dhahbi JM, Chen JW, Bhupathy S, Atamna H, Cavalcante MB, Saccon TD, et al. Specific PIWI-Interacting RNAs and Related Small Noncoding RNAs Are Associated With Ovarian Aging in Ames Dwarf (df/df) Mice. J Gerontol A Biol Sci Med Sci. 2021;76(9):1561-70. doi: 10.1093/gerona/glab113. PubMed PMID: 34387333; PubMed Central PMCID: PMCPMC8361361.

57. Dietrich D, Meller S, Uhl B, Ralla B, Stephan C, Jung K, et al. Nucleic acid-based tissue biomarkers of urologic malignancies. Crit Rev Clin Lab Sci. 2014;51(4):173-99. Epub 20140530. doi: 10.3109/10408363.2014.906130. PubMed PMID: 24878394.

58. Dong P, Xiong Y, Konno Y, Ihira K, Xu D, Kobayashi N, et al. Critical Roles of PIWIL1 in Human Tumors: Expression, Functions, Mechanisms, and Potential Clinical Implications. Front Cell Dev Biol. 2021;9:656993. Epub 20210226. doi: 10.3389/fcell.2021.656993. PubMed PMID: 33718392; PubMed Central PMCID: PMCPMC7952444.

59. Du X, Li H, Xie X, Shi L, Wu F, Li G, et al. piRNA-31115 Promotes Cell Proliferation and Invasion via PI3K/AKT Pathway in Clear Cell Renal Carcinoma. Dis Markers. 2021;2021:6915329. Epub 20211108. doi: 10.1155/2021/6915329. PubMed PMID: 34790278; PubMed Central PMCID: PMCPMC8592738.

60. Dvorská D, Braný D, Ňachajová M, Halašová E, Danková Z. Breast Cancer and the Other Non-Coding RNAs. Int J Mol Sci. 2021;22(6). Epub 20210323. doi: 10.3390/ijms22063280. PubMed PMID: 33807045; PubMed Central PMCID: PMCPMC8005115.

61. Dwivedi SKD, Rao G, Dey A, Mukherjee P, Wren JD, Bhattacharya R. Small Non-Coding-RNA in Gynecological Malignancies. Cancers (Basel). 2021;13(5). Epub 20210303. doi: 10.3390/cancers13051085. PubMed PMID: 33802524; PubMed Central PMCID: PMCPMC7961667.

62. Eastwood EL, Jara KA, Bornelöv S, Munafò M, Frantzis V, Kneuss E, et al. Dimerisation of the PICTS complex via LC8/Cut-up drives co-transcriptional transposon silencing in Drosophila. Elife. 2021;10. Epub 20210204. doi: 10.7554/eLife.65557. PubMed PMID: 33538693; PubMed Central PMCID: PMCPMC7861614.

63. ElMaghraby MF, Tirian L, Senti KA, Meixner K, Brennecke J. A genetic toolkit for studying transposon control in the Drosophila melanogaster ovary. Genetics. 2022;220(1). doi: 10.1093/genetics/iyab179. PubMed PMID: 34718559; PubMed Central PMCID: PMCPMC8733420.

64. Enfield KS, Martinez VD, Marshall EA, Stewart GL, Kung SH, Enterina JR, et al. Deregulation of small non-coding RNAs at the DLK1-DIO3 imprinted locus predicts lung cancer patient outcome. Oncotarget. 2016;7(49):80957-66. doi: 10.18632/oncotarget.13133. PubMed PMID: 27829231; PubMed Central PMCID: PMCPMC5348368.

65. Ernst C, Odom DT, Kutter C. The emergence of piRNAs against transposon invasion to preserve mammalian genome integrity. Nat Commun. 2017;8(1):1411. Epub 20171110. doi: 10.1038/s41467-017-01049-7. PubMed PMID: 29127279; PubMed Central PMCID: PMCPMC5681665.

66. Eslava-Avilés E, Arenas-Huertero F. piRNAs: nature, biogenesis, regulation, and their potential clinical utility. Bol Med Hosp Infant Mex. 2021;78(5):432-42. doi: 10.24875/bmhim.20000185. PubMed PMID: 34571517.

67. Fabry MH, Falconio FA, Joud F, Lythgoe EK, Czech B, Hannon GJ. Maternally inherited piRNAs direct transient heterochromatin formation at active transposons during early Drosophila embryogenesis. Elife. 2021;10. Epub 20210708. doi: 10.7554/eLife.68573. PubMed PMID: 34236313; PubMed Central PMCID: PMCPMC8352587.

68. Fathizadeh H, Asemi Z. Epigenetic roles of PIWI proteins and piRNAs in lung cancer. Cell Biosci. 2019;9:102. Epub 20191221. doi: 10.1186/s13578-019-0368-x. PubMed PMID:

31890151; PubMed Central PMCID: PMCPMC6925842.

69. Feller SM, Lewitzky M. Hunting for the ultimate liquid cancer biopsy - let the TEP dance begin. Cell Commun Signal. 2016;14(1):24. Epub 20160927. doi: 10.1186/s12964-016-0147-9. PubMed PMID: 27677261; PubMed Central PMCID: PMCPMC5039897.

70. Feng J, Yang M, Wei Q, Song F, Zhang Y, Wang X, et al. Novel evidence for oncogenic piRNA-823 as a promising prognostic biomarker and a potential therapeutic target in colorectal cancer. J Cell Mol Med. 2020;24(16):9028-40. Epub 20200628. doi: 10.1111/jcmm.15537. PubMed PMID: 32596991; PubMed Central PMCID: PMCPMC7417729.

71. Fernandez-Calero T, Garcia-Silva R, Pena A, Robello C, Persson H, Rovira C, et al. Profiling of small RNA cargo of extracellular vesicles shed by Trypanosoma cruzi reveals a specific extracellular signature. Mol Biochem Parasitol. 2015;199(1-2):19-28. Epub 20150317. doi: 10.1016/j.molbiopara.2015.03.003. PubMed PMID: 25795082.

72. Ferrero G, Cordero F, Tarallo S, Arigoni M, Riccardo F, Gallo G, et al. Small non-coding RNA profiling in human biofluids and surrogate tissues from healthy individuals: description of the diverse and most represented species. Oncotarget. 2018;9(3):3097-111. Epub 20171214. doi: 10.18632/oncotarget.23203. PubMed PMID: 29423032; PubMed Central PMCID: PMCPMC5790449.

73. Firmino N, Martinez VD, Rowbotham DA, Enfield KSS, Bennewith KL, Lam WL. HPV status is associated with altered PIWI-interacting RNA expression pattern in head and neck cancer. Oral Oncol. 2016;55:43-8. Epub 20160204. doi: 10.1016/j.oraloncology.2016.01.012. PubMed PMID: 26852287; PubMed Central PMCID: PMCPMC4808439.

74. Fonseca Cabral G, Azevedo Dos Santos Pinheiro J, Vidal AF, Santos S, Ribeiro-Dos-Santos Â. piRNAs in Gastric Cancer: A New Approach Towards Translational Research. Int J Mol Sci. 2020;21(6). Epub 20200319. doi: 10.3390/ijms21062126. PubMed PMID: 32204558; PubMed Central PMCID: PMCPMC7139476.

75. Frau M, Feo CF, Feo F, Pascale RM. New insights on the role of epigenetic alterations in hepatocellular carcinoma. J Hepatocell Carcinoma. 2014;1:65-83. Epub 20140612. doi: 10.2147/jhc.S44506. PubMed PMID: 27508177; PubMed Central PMCID: PMCPMC4918272.

76. Fu A, Jacobs DI, Hoffman AE, Zheng T, Zhu Y. PIWI-interacting RNA 021285 is involved in breast tumorigenesis possibly by remodeling the cancer epigenome. Carcinogenesis. 2015;36(10):1094-102. Epub 20150725. doi: 10.1093/carcin/bgv105. PubMed PMID: 26210741; PubMed Central PMCID: PMCPMC5006152.

77. Fu A, Jacobs DI, Zhu Y. Epigenome-wide analysis of piRNAs in gene-specific DNA methylation. RNA Biol. 2014;11(10):1301-12. doi: 10.1080/15476286.2014.996091. PubMed PMID: 25590657; PubMed Central PMCID: PMCPMC4615395.

78. Gainetdinov IV, Skvortsova YV, Kondratieva SA, Klimov A, Tryakin AA, Azhikina TL. Assessment of piRNA biogenesis and function in testicular germ cell tumors and their precursor germ cell neoplasia in situ. BMC Cancer. 2018;18(1):20. Epub 20180104. doi: 10.1186/s12885-017-3945-6. PubMed PMID: 29301509; PubMed Central PMCID: PMCPMC5755174.

79. Gambichler T, Kohsik C, Höh AK, Lang K, Käfferlein HU, Brüning T, et al. Expression of PIWIL3 in primary and metastatic melanoma. J Cancer Res Clin Oncol. 2017;143(3):433-7. Epub 20161117. doi: 10.1007/s00432-016-2305-2. PubMed PMID: 27858163.

80. Ge L, Zhang N, Li D, Wu Y, Wang H, Wang J. Circulating exosomal small RNAs are promising non-invasive diagnostic biomarkers for gastric cancer. J Cell Mol Med. 2020;24(24):14502-13. Epub 20201109. doi: 10.1111/jcmm.16077. PubMed PMID: 33169519; PubMed Central PMCID: PMCPMC7753781.

81. Ghazimoradi MH, Karimpour-Fard N, Babashah S. The Promising Role of Non-Coding RNAs as Biomarkers and Therapeutic Targets for Leukemia. Genes (Basel). 2023;14(1). Epub 20230103. doi: 10.3390/genes14010131. PubMed PMID: 36672872; PubMed Central PMCID: PMCPMC9859176.

82. Ghosh B, Sarkar A, Mondal S, Bhattacharya N, Khatua S, Ghosh Z. piRNAQuest V.2: an updated resource for searching through the piRNAome of multiple species. RNA Biol. 2022;19(1):12-25. Epub 20211231. doi: 10.1080/15476286.2021.2010960. PubMed PMID: 34965192; PubMed Central PMCID: PMCPMC8786328.

83. Giraldez MD, Spengler RM, Etheridge A, Godoy PM, Barczak AJ, Srinivasan S, et al. Comprehensive multi-center assessment of small RNA-seq methods for quantitative miRNA profiling. Nat Biotechnol. 2018;36(8):746-57. Epub 20180716. doi: 10.1038/nbt.4183. PubMed PMID: 30010675; PubMed Central PMCID: PMCPMC6078798.

84. Giurato G, De Filippo MR, Rinaldi A, Hashim A, Nassa G, Ravo M, et al. iMir: an integrated pipeline for high-throughput analysis of small non-coding RNA data obtained by smallRNA-Seq. BMC Bioinformatics. 2013;14:362. Epub 20131213. doi: 10.1186/1471-2105-14-362. PubMed PMID: 24330401; PubMed Central PMCID: PMCPMC3878829.

85. Gomes AQ, Nolasco S, Soares H. Non-coding RNAs: multi-tasking molecules in the cell. Int J Mol Sci. 2013;14(8):16010-39. Epub 20130731. doi: 10.3390/ijms140816010. PubMed PMID: 23912238; PubMed Central PMCID: PMCPMC3759897.

86. Grixti JM, Ayers D. Long noncoding RNAs and their link to cancer. Noncoding RNA Res. 2020;5(2):77-82. Epub 20200513. doi: 10.1016/j.ncrna.2020.04.003. PubMed PMID: 32490292; PubMed Central PMCID: PMCPMC7256057.

87. Gu X, Wang C, Deng H, Qing C, Liu R, Liu S, et al. Exosomal piRNA profiling revealed unique circulating piRNA signatures of cholangiocarcinoma and gallbladder carcinoma. Acta Biochim Biophys Sin (Shanghai). 2020;52(5):475-84. doi: 10.1093/abbs/gmaa028. PubMed PMID: 32369104.

88. Guan Y, Keeney S, Jain D, Wang PJ. yama, a mutant allele of Mov10l1, disrupts retrotransposon silencing and piRNA biogenesis. PLoS Genet. 2021;17(2):e1009265. Epub 20210226. doi: 10.1371/journal.pgen.1009265. PubMed PMID: 33635934; PubMed Central PMCID: PMCPMC7946307.

89. Guo X, Qiu W, Garcia-Milian R, Lin X, Zhang Y, Cao Y, et al. Genome-wide significant, replicated and functional risk variants for Alzheimer's disease. J Neural Transm (Vienna). 2017;124(11):1455-71. Epub 20170802. doi: 10.1007/s00702-017-1773-0. PubMed PMID: 28770390; PubMed Central PMCID: PMCPMC5654670.

90. Halajzadeh J, Dana PM, Asemi Z, Mansournia MA, Yousefi B. An insight into the roles of piRNAs and PIWI proteins in the diagnosis and pathogenesis of oral, esophageal, and gastric cancer. Pathol Res Pract. 2020;216(10):153112. Epub 20200713. doi: 10.1016/j.prp.2020.153112. PubMed PMID: 32853949.

91. Hallal S, Ebrahim Khani S, Wei H, Lee MYT, Sim HW, Sy J, et al. Deep Sequencing of Small

RNAs from Neurosurgical Extracellular Vesicles Substantiates miR-486-3p as a Circulating Biomarker that Distinguishes Glioblastoma from Lower-Grade Astrocytoma Patients. Int J Mol Sci. 2020;21(14). Epub 20200713. doi: 10.3390/ijms21144954. PubMed PMID: 32668808; PubMed Central PMCID: PMCPMC7404297.

92. Han YN, Li Y, Xia SQ, Zhang YY, Zheng JH, Li W. PIWI Proteins and PIWI-Interacting RNA: Emerging Roles in Cancer. Cell Physiol Biochem. 2017;44(1):1-20. Epub 20171103. doi: 10.1159/000484541. PubMed PMID: 29130960.

93. Hanusek K, Poletajew S, Kryst P, Piekiełko-Witkowska A, Bogusławska J. piRNAs and PIWI Proteins as Diagnostic and Prognostic Markers of Genitourinary Cancers. Biomolecules. 2022;12(2). Epub 20220122. doi: 10.3390/biom12020186. PubMed PMID: 35204687; PubMed Central PMCID: PMCPMC8869487.

94. Hashim A, Rizzo F, Marchese G, Ravo M, Tarallo R, Nassa G, et al. RNA sequencing identifies specific PIWI-interacting small non-coding RNA expression patterns in breast cancer. Oncotarget. 2014;5(20):9901-10. doi: 10.18632/oncotarget.2476. PubMed PMID: 25313140; PubMed Central PMCID: PMCPMC4259446.

95. Hayashi K, Chuva de Sousa Lopes SM, Kaneda M, Tang F, Hajkova P, Lao K, et al. MicroRNA biogenesis is required for mouse primordial germ cell development and spermatogenesis. PLoS One. 2008;3(3):e1738. Epub 20080305. doi: 10.1371/journal.pone.0001738. PubMed PMID: 18320056; PubMed Central PMCID: PMCPMC2254191.

96. He X, Chen X, Zhang X, Duan X, Pan T, Hu Q, et al. An Lnc RNA (GAS5)/SnoRNA-derived piRNA induces activation of TRAIL gene by site-specifically recruiting MLL/COMPASS-like complexes. Nucleic Acids Res. 2015;43(7):3712-25. Epub 20150316. doi: 10.1093/nar/gkv214. PubMed PMID: 25779046; PubMed Central PMCID: PMCPMC4402533.

97. He Y, Meng XM, Huang C, Wu BM, Zhang L, Lv XW, et al. Long noncoding RNAs: Novel insights into hepatocelluar carcinoma. Cancer Lett. 2014;344(1):20-7. Epub 20131030. doi: 10.1016/j.canlet.2013.10.021. PubMed PMID: 24183851.

98. Hempfling AL, Lim SL, Adelson DL, Evans J, O'Connor AE, Qu ZP, et al. Expression patterns of HENMT1 and PIWIL1 in human testis: implications for transposon expression. Reproduction. 2017;154(4):363-74. Epub 20170704. doi: 10.1530/rep-16-0586. PubMed PMID: 28676534.

99. Heng B, Xie X, Zeng W, Li H, Shi L, Ye W, et al. PIWI-Interacting RNA Pathway Genes: Potential Biomarkers for Clear Cell Renal Cell Carcinoma. Dis Markers. 2022;2022:3480377. Epub 20220301. doi: 10.1155/2022/3480377. PubMed PMID: 35273654; PubMed Central PMCID: PMCPMC8904100.

100. Holt JE, Stanger SJ, Nixon B, McLaughlin EA. Non-coding RNA in Spermatogenesis and Epididymal Maturation. Adv Exp Med Biol. 2016;886:95-120. doi: 10.1007/978-94-017-7417-8_6. PubMed PMID: 26659489.

101. Hombach S, Kretz M. Non-coding RNAs: Classification, Biology and Functioning. Adv Exp Med Biol. 2016;937:3-17. doi: 10.1007/978-3-319-42059-2_1. PubMed PMID: 27573892.

102. Hong Y, Wang C, Fu Z, Liang H, Zhang S, Lu M, et al. Systematic characterization of seminal plasma piRNAs as molecular biomarkers for male infertility. Sci Rep. 2016;6:24229. Epub 20160412. doi: 10.1038/srep24229. PubMed PMID: 27068805; PubMed Central PMCID: PMCPMC4828650.

103. Hosseinalizadeh H, Mahmoodpour M, Ebrahimi A. Circulating non-coding RNAs as a diagnostic and management biomarker for breast cancer: current insights. Mol Biol Rep. 2022;49(1):705-15. Epub 20211022. doi: 10.1007/s11033-021-06847-3. PubMed PMID: 34677714.

104. Huang G, Hu H, Xue X, Shen S, Gao E, Guo G, et al. Altered expression of piRNAs and their relation with clinicopathologic features of breast cancer. Clin Transl Oncol. 2013;15(7):563-8. Epub 20121115. doi: 10.1007/s12094-012-0966-0. PubMed PMID: 23229900.

105. Huang X, Yuan T, Tschannen M, Sun Z, Jacob H, Du M, et al. Characterization of human plasma-derived exosomal RNAs by deep sequencing. BMC Genomics. 2013;14:319. Epub 20130510. doi: 10.1186/1471-2164-14-319. PubMed PMID: 23663360; PubMed Central PMCID: PMCPMC3653748.

106. Hutcheon K, McLaughlin EA, Stanger SJ, Bernstein IR, Dun MD, Eamens AL, et al. Analysis of the small non-protein-coding RNA profile of mouse spermatozoa reveals specific enrichment of piRNAs within mature spermatozoa. RNA Biol. 2017;14(12):1776-90. Epub 20170921. doi: 10.1080/15476286.2017.1356569. PubMed PMID: 28816603; PubMed Central PMCID: PMCPMC5731817.

107. Iliev R, Fedorko M, Machackova T, Mlcochova H, Svoboda M, Pacik D, et al. Expression Levels of PIWI-interacting RNA, piR-823, Are Deregulated in Tumor Tissue, Blood Serum and Urine of Patients with Renal Cell Carcinoma. Anticancer Res. 2016;36(12):6419-23. doi: 10.21873/anticanres.11239. PubMed PMID: 27919963.

108. Iliev R, Stanik M, Fedorko M, Poprach A, Vychytilova-Faltejskova P, Slaba K, et al. Decreased expression levels of PIWIL1, PIWIL2, and PIWIL4 are associated with worse survival in renal cell carcinoma patients. Onco Targets Ther. 2016;9:217-22. Epub 20160108. doi: 10.2147/ott.S91295. PubMed PMID: 26811690; PubMed Central PMCID: PMCPMC4712976.

109. Irimie AI, Braicu C, Sonea L, Zimta AA, Cojocneanu-Petric R, Tonchev K, et al. A Looking-Glass of Non-coding RNAs in oral cancer. Int J Mol Sci. 2017;18(12). Epub 20171205. doi: 10.3390/ijms18122620. PubMed PMID: 29206174; PubMed Central PMCID: PMCPMC5751223.

110. Ishizu H, Sumiyoshi T, Siomi MC. Use of the CRISPR-Cas9 system for genome editing in cultured Drosophila ovarian somatic cells. Methods. 2017;126:186-92. Epub 20170525. doi: 10.1016/j.ymeth.2017.05.021. PubMed PMID: 28552546.

111. Iyer DN, Wan TM, Man JH, Sin RW, Li X, Lo OS, et al. Small RNA Profiling of piRNAs in Colorectal Cancer Identifies Consistent Overexpression of piR-24000 That Correlates Clinically with an Aggressive Disease Phenotype. Cancers (Basel). 2020;12(1). Epub 20200112. doi: 10.3390/cancers12010188. PubMed PMID: 31940941; PubMed Central PMCID: PMCPMC7016796.

112. Jacobs DI, Qin Q, Lerro MC, Fu A, Dubrow R, Claus EB, et al. PIWI-Interacting RNAs in Gliomagenesis: Evidence from Post-GWAS and Functional Analyses. Cancer Epidemiol Biomarkers Prev. 2016;25(7):1073-80. Epub 20160513. doi: 10.1158/1055-9965.Epi-16-0047. PubMed PMID: 27197292.

113. Jia DD, Jiang H, Zhang YF, Zhang Y, Qian LL, Zhang YF. The regulatory function of piRNA/PIWI complex in cancer and other human diseases: The role of DNA methylation. Int J Biol Sci. 2022;18(8):3358-73. Epub 20220509. doi: 10.7150/ijbs.68221. PubMed PMID: 35637965; PubMed Central PMCID: PMCPMC9134905.

114. Jia J, Yang S, Huang J, Zheng H, He Y, Wang L. Distinct Extracellular RNA Profiles in Different Plasma Components. Front Genet. 2021;12:564780. Epub 20210621. doi: 10.3389/fgene.2021.564780. PubMed PMID: 34234804; PubMed Central PMCID: PMCPMC8256274.

115. Jia R, He X, Ma W, Lei Y, Cheng H, Sun H, et al. Aptamer-Functionalized Activatable DNA Tetrahedron Nanoprobe for PIWI-Interacting RNA Imaging and Regulating in Cancer Cells. Anal Chem. 2019;91(23):15107-13. Epub 20191115. doi: 10.1021/acs.analchem.9b03819. PubMed PMID: 31691558.

116. Jin Z, Flynt AS, Lai EC. Drosophila piwi mutants exhibit germline stem cell tumors that are sustained by elevated Dpp signaling. Curr Biol. 2013;23(15):1442-8. Epub 20130725. doi: 10.1016/j.cub.2013.06.021. PubMed PMID: 23891114; PubMed Central PMCID: PMCPMC3740069.

117. Joh RI, Palmieri CM, Hill IT, Motamedi M. Regulation of histone methylation by noncoding RNAs. Biochim Biophys Acta. 2014;1839(12):1385-94. Epub 20140617. doi: 10.1016/j.bbagrm.2014.06.006. PubMed PMID: 24954181; PubMed Central PMCID: PMCPMC4258512.

118. Juvvuna PK, Khandelia P, Lee LM, Makeyev EV. Argonaute identity defines the length of mature mammalian microRNAs. Nucleic Acids Res. 2012;40(14):6808-20. Epub 20120413. doi: 10.1093/nar/gks293. PubMed PMID: 22505576; PubMed Central PMCID: PMCPMC3413106.

119. Kamminga LM, van Wolfswinkel JC, Luteijn MJ, Kaaij LJ, Bagijn MP, Sapetschnig A, et al. Differential impact of the HEN1 homolog HENN-1 on 21U and 26G RNAs in the germline of Caenorhabditis elegans. PLoS Genet. 2012;8(7):e1002702. Epub 20120719. doi: 10.1371/journal.pgen.1002702. PubMed PMID: 22829772; PubMed Central PMCID: PMCPMC3400576.

120. Kar S, Geffers R, Samii A, Bertalanffy H. Library Preparation for Small RNA Transcriptome Sequencing in Patients Affected by Cerebral Cavernous Malformations. Methods Mol Biol. 2020;2152:467-78. doi: 10.1007/978-1-0716-0640-7_35. PubMed PMID: 32524574.

121. Karam JA, Parikh RY, Nayak D, Rosenkranz D, Gangaraju VK. Co-chaperone Hsp70/Hsp90-organizing protein (Hop) is required for transposon silencing and Piwi-interacting RNA (piRNA) biogenesis. J Biol Chem. 2017;292(15):6039-46. Epub 20170213. doi: 10.1074/jbc.C117.777730. PubMed PMID: 28193840; PubMed Central PMCID: PMCPMC5391737.

122. Kärkkäinen E, Heikkinen S, Tengström M, Kosma VM, Mannermaa A, Hartikainen JM. The debatable presence of PIWI-interacting RNAs in invasive breast cancer. Cancer Med. 2021;10(11):3593-603. Epub 20210507. doi: 10.1002/cam4.3915. PubMed PMID: 33960684; PubMed Central PMCID: PMCPMC8178507.

123. Kazimierczyk M, Jędroszkowiak A, Kowalczykiewicz D, Szymański M, Imiołczyk B, Ciesiołka J, et al. tRNA-derived fragments from the Sus scrofa tissues provide evidence of their conserved role in mammalian development. Biochem Biophys Res Commun. 2019;520(3):514-9. Epub 20191011. doi: 10.1016/j.bbrc.2019.10.062. PubMed PMID: 31610915.

124. Khalid M, Bojang P, Jr., Hassanin AAI, Bowers EC, Reyes-Reyes EM, Ramos IN, et al. Line-1: Implications in the etiology of cancer, clinical applications, and pharmacologic targets. Mutat

Res Rev Mutat Res. 2018;778:51-60. Epub 20180919. doi: 10.1016/j.mrrev.2018.09.003. PubMed PMID: 30454683.

125. Kharel P, Balaratnam S, Beals N, Basu S. The role of RNA G-quadruplexes in human diseases and therapeutic strategies. Wiley Interdiscip Rev RNA. 2020;11(1):e1568. Epub 20190912. doi: 10.1002/wrna.1568. PubMed PMID: 31514263.

126. Kim SH, Park ER, Cho E, Jung WH, Jeon JY, Joo HY, et al. Mael is essential for cancer cell survival and tumorigenesis through protection of genetic integrity. Oncotarget. 2017;8(3):5026-37. doi: 10.18632/oncotarget.13756. PubMed PMID: 27926513; PubMed Central PMCID: PMCPMC5354889.

127. Kishikawa T, Otsuka M, Ohno M, Yoshikawa T, Takata A, Koike K. Circulating RNAs as new biomarkers for detecting pancreatic cancer. World J Gastroenterol. 2015;21(28):8527-40. doi: 10.3748/wjg.v21.i28.8527. PubMed PMID: 26229396; PubMed Central PMCID: PMCPMC4515835.

128. Kishore C, Karunagaran D. Non-coding RNAs as emerging regulators and biomarkers in colorectal cancer. Mol Cell Biochem. 2022;477(6):1817-28. Epub 20220324. doi: 10.1007/s11010-022-04412-5. PubMed PMID: 35332394.

129. Kneuss E, Munafò M, Eastwood EL, Deumer US, Preall JB, Hannon GJ, et al. Specialization of the Drosophila nuclear export family protein Nxf3 for piRNA precursor export. Genes Dev. 2019;33(17-18):1208-20. Epub 20190815. doi: 10.1101/gad.328690.119. PubMed PMID: 31416967; PubMed Central PMCID: PMCPMC6719614.

130. Kocic G, Hadzi-Djokic J, Colic M, Veljkovic A, Tomovic K, Roumeliotis S, et al. The Role of Nucleases Cleaving TLR3, TLR7/8 and TLR9 Ligands, Dicer RNase and miRNA/piRNA Proteins in Functional Adaptation to the Immune Escape and Xenophagy of Prostate Cancer Tissue. Int J Mol Sci. 2022;24(1). Epub 20221228. doi: 10.3390/ijms24010509. PubMed PMID: 36613950; PubMed Central PMCID: PMCPMC9820234.

131. Koduru SV, Leberfinger AN, Kawasawa YI, Mahajan M, Gusani NJ, Sanyal AJ, et al. Non-coding RNAs in Various Stages of Liver Disease Leading to Hepatocellular Carcinoma: Differential Expression of miRNAs, piRNAs, lncRNAs, circRNAs, and sno/mt-RNAs. Sci Rep. 2018;8(1):7967. Epub 20180522. doi: 10.1038/s41598-018-26360-1. PubMed PMID: 29789629; PubMed Central PMCID: PMCPMC5964116.

132. Koduru SV, Leberfinger AN, Ravnic DJ. Small Non-coding RNA Abundance in Adrenocortical Carcinoma: A Footprint of a Rare Cancer. J Genomics. 2017;5:99-118. Epub 20170908. doi: 10.7150/jgen.22060. PubMed PMID: 28943972; PubMed Central PMCID: PMCPMC5607708.

133. Koduru SV, Tiwari AK, Hazard SW, Mahajan M, Ravnic DJ. Exploration of small RNA-seq data for small non-coding RNAs in Human Colorectal Cancer. J Genomics. 2017;5:16-31. Epub 20170228. doi: 10.7150/jgen.18856. PubMed PMID: 28348640; PubMed Central PMCID: PMCPMC5362762.

134. Koduru SV, Tiwari AK, Leberfinger A, Hazard SW, Kawasawa YI, Mahajan M, et al. A Comprehensive NGS Data Analysis of Differentially Regulated miRNAs, piRNAs, lncRNAs and sn/snoRNAs in Triple Negative Breast Cancer. J Cancer. 2017;8(4):578-96. Epub 20170211. doi: 10.7150/jca.17633. PubMed PMID: 28367238; PubMed Central PMCID: PMCPMC5370502.

135. Kolenda T, Guglas K, Baranowski D, Sobocińska J, Kopczyńska M, Teresiak A, et al. cfRNAs

as biomarkers in oncology - still experimental or applied tool for personalized medicine already? Rep Pract Oncol Radiother. 2020;25(5):783-92. Epub 20200811. doi: 10.1016/j.rpor.2020.07.007. PubMed PMID: 32904167; PubMed Central PMCID: PMCPMC7451588.

136. Kovalchuk A, Ilnytskyy Y, Rodriguez-Juarez R, Katz A, Sidransky D, Kolb B, et al. Growth of malignant extracranial tumors alters microRNAome in the prefrontal cortex of TumorGraft mice. Oncotarget. 2017;8(51):88276-93. Epub 20170803. doi: 10.18632/oncotarget.19835. PubMed PMID: 29179434; PubMed Central PMCID: PMCPMC5687604.

137. Kovalenko TF, Larionova TD, Antipova NV, Shakhparonov MI, Pavlyukov MS. The Role of Non-coding RNAs in the Pathogenesis of Glial Tumors. Acta Naturae. 2021;13(3):38-51. doi: 10.32607/actanaturae.11270. PubMed PMID: 34707896; PubMed Central PMCID: PMCPMC8526181.

138. Krishnan AR, Korrapati A, Zou AE, Qu Y, Wang XQ, Califano JA, et al. Smoking status regulates a novel panel of PIWI-interacting RNAs in head and neck squamous cell carcinoma. Oral Oncol. 2017;65:68-75. Epub 20161231. doi: 10.1016/j.oraloncology.2016.12.022. PubMed PMID: 28109471; PubMed Central PMCID: PMCPMC5291169.

139. Krishnan P, Damaraju S. The Challenges and Opportunities in the Clinical Application of Noncoding RNAs: The Road Map for miRNAs and piRNAs in Cancer Diagnostics and Prognostics. Int J Genomics. 2018;2018:5848046. Epub 20180430. doi: 10.1155/2018/5848046. PubMed PMID: 29854719; PubMed Central PMCID: PMCPMC5952559.

140. Krishnan P, Ghosh S, Graham K, Mackey JR, Kovalchuk O, Damaraju S. Piwi-interacting RNAs and PIWI genes as novel prognostic markers for breast cancer. Oncotarget. 2016;7(25):37944-56. doi: 10.18632/oncotarget.9272. PubMed PMID: 27177224; PubMed Central PMCID: PMCPMC5122362.

141. Kugelberg U, Nätt D, Skog S, Kutter C, Öst A. 5´XP sRNA-seq: efficient identification of transcripts with and without 5´ phosphorylation reveals evolutionary conserved small RNA. RNA Biol. 2021;18(11):1588-99. Epub 20201231. doi: 10.1080/15476286.2020.1861770. PubMed PMID: 33382953; PubMed Central PMCID: PMCPMC8594926.

142. Kumar SR, Kimchi ET, Manjunath Y, Gajagowni S, Stuckel AJ, Kaifi JT. RNA cargos in extracellular vesicles derived from blood serum in pancreas associated conditions. Sci Rep. 2020;10(1):2800. Epub 20200218. doi: 10.1038/s41598-020-59523-0. PubMed PMID: 32071328; PubMed Central PMCID: PMCPMC7028741.

143. Kumaran M, Krishnan P, Cass CE, Hubaux R, Lam W, Yasui Y, et al. Breast cancer associated germline structural variants harboring small noncoding RNAs impact post-transcriptional gene regulation. Sci Rep. 2018;8(1):7529. Epub 20180514. doi: 10.1038/s41598-018-25801-1. PubMed PMID: 29760470; PubMed Central PMCID: PMCPMC5951800.

144. Kunnummal M, Angelin M, Das AV. PIWI proteins and piRNAs in cervical cancer: a propitious dart in cancer stem cell-targeted therapy. Hum Cell. 2021;34(6):1629-41. Epub 20210809. doi: 10.1007/s13577-021-00590-4. PubMed PMID: 34374035.

145. Kuo MC, Liu SC, Hsu YF, Wu RM. The role of noncoding RNAs in Parkinson's disease: biomarkers and associations with pathogenic pathways. J Biomed Sci. 2021;28(1):78. Epub 20211118. doi: 10.1186/s12929-021-00775-x. PubMed PMID: 34794432; PubMed Central PMCID: PMCPMC8603508.

146. Kutter C, Svoboda P. miRNA, siRNA, piRNA: Knowns of the unknown. RNA Biol. 2008;5(4):181-8. Epub 20081016. doi: 10.4161/rna.7227. PubMed PMID: 19182524.

147. Kwon C, Tak H, Rho M, Chang HR, Kim YH, Kim KT, et al. Detection of PIWI and piRNAs in the mitochondria of mammalian cancer cells. Biochem Biophys Res Commun. 2014;446(1):218-23. Epub 20140303. doi: 10.1016/j.bbrc.2014.02.112. PubMed PMID: 24602614.

148. Kwon NH, Lee JY, Kim S. Role of tRNAs in Breast Cancer Regulation. Adv Exp Med Biol. 2021;1187:121-45. doi: 10.1007/978-981-32-9620-6_6. PubMed PMID: 33983576.

149. Lampropoulou DI, Pliakou E, Aravantinos G, Filippou D, Gazouli M. The Role of Exosomal Non-Coding RNAs in Colorectal Cancer Drug Resistance. Int J Mol Sci. 2022;23(3). Epub 20220127. doi: 10.3390/ijms23031473. PubMed PMID: 35163397; PubMed Central PMCID: PMCPMC8835818.

150. Laschos K, Lampropoulou DI, Aravantinos G, Piperis M, Filippou D, Theodoropoulos G, et al. Exosomal noncoding RNAs in cholangiocarcinoma: Laboratory noise or hope? World J Gastrointest Surg. 2020;12(10):407-24. doi: 10.4240/wjgs.v12.i10.407. PubMed PMID: 33194090; PubMed Central PMCID: PMCPMC7642347.

151. Laudadio I, Formichetti S, Gioiosa S, Klironomos F, Rajewsky N, Macino G, et al. Characterization of Transcription Termination-Associated RNAs: New Insights into their Biogenesis, Tailing, and Expression in Primary Tumors. Int J Genomics. 2018;2018:1243858. Epub 20180426. doi: 10.1155/2018/1243858. PubMed PMID: 29854718; PubMed Central PMCID: PMCPMC5944193.

152. Le Thomas A, Stuwe E, Li S, Du J, Marinov G, Rozhkov N, et al. Transgenerationally inherited piRNAs trigger piRNA biogenesis by changing the chromatin of piRNA clusters and inducing precursor processing. Genes Dev. 2014;28(15):1667-80. doi: 10.1101/gad.245514.114. PubMed PMID: 25085419; PubMed Central PMCID: PMCPMC4117942.

153. Lee KY, Seo Y, Im JH, Rhim J, Baek W, Kim S, et al. Molecular Signature of Extracellular Vesicular Small Non-Coding RNAs Derived from Cerebrospinal Fluid of Leptomeningeal Metastasis Patients: Functional Implication of miR-21 and Other Small RNAs in Cancer Malignancy. Cancers (Basel). 2021;13(2). Epub 20210108. doi: 10.3390/cancers13020209. PubMed PMID: 33430103; PubMed Central PMCID: PMCPMC7828086.

154. Lee SM, Winters MT, Martinez I, Slack FJ. Small regulatory RNAs: from bench to bedside - a keystone symposia meeting report. RNA Biol. 2023;20(1):136-9. doi: 10.1080/15476286.2023.2196046. PubMed PMID: 37016725; PubMed Central PMCID: PMCPMC10078130.

155. Lee YJ, Moon SU, Park MG, Jung WY, Park YK, Song SK, et al. Multiplex bioimaging of piRNA molecular pathway-regulated theragnostic effects in a single breast cancer cell using a piRNA molecular beacon. Biomaterials. 2016;101:143-55. Epub 20160601. doi: 10.1016/j.biomaterials.2016.05.052. PubMed PMID: 27289065.

156. Lewis A, Berkyurek AC, Greiner A, Sawh AN, Vashisht A, Merrett S, et al. A Family of Argonaute-Interacting Proteins Gates Nuclear RNAi. Mol Cell. 2020;78(5):862-75.e8. Epub 20200428. doi: 10.1016/j.molcel.2020.04.007. PubMed PMID: 32348780; PubMed Central PMCID: PMCPMC7613089.

157. Lewis SH, Quarles KA, Yang Y, Tanguy M, Frézal L, Smith SA, et al. Pan-arthropod analysis

reveals somatic piRNAs as an ancestral defence against transposable elements. Nat Ecol Evol. 2018;2(1):174-81. Epub 20171204. doi: 10.1038/s41559-017-0403-4. PubMed PMID: 29203920; PubMed Central PMCID: PMCPMC5732027.

158. Li F, Yuan P, Rao M, Jin CH, Tang W, Rong YF, et al. piRNA-independent function of PIWIL1 as a co-activator for anaphase promoting complex/cyclosome to drive pancreatic cancer metastasis. Nat Cell Biol. 2020;22(4):425-38. Epub 20200316. doi: 10.1038/s41556-020-0486-z. PubMed PMID: 32203416.

159. Li G, Wang X, Li C, Hu S, Niu Z, Sun Q, et al. Piwi-Interacting RNA1037 Enhances Chemoresistance and Motility in Human Oral Squamous Cell Carcinoma Cells. Onco Targets Ther. 2019;12:10615-27. Epub 20191204. doi: 10.2147/ott.S233322. PubMed PMID: 31824169; PubMed Central PMCID: PMCPMC6900317.

160. Li J, Tong Y, Sun Z, Chen Y, Wang Y, Zhou L, et al. A duplex-specific nuclease assisted photoelectrochemical biosensor based on MoS(2)@ReS(2)/Ti(3)C(2) hybrid for ultrasensitive detection of colorectal cancer-related piRNA-31,143. Acta Biomater. 2022;149:287-96. Epub 20220625. doi: 10.1016/j.actbio.2022.06.037. PubMed PMID: 35764241.

161. Li J, Wang N, Zhang F, Jin S, Dong Y, Dong X, et al. PIWI-interacting RNAs are aberrantly expressed and may serve as novel biomarkers for diagnosis of lung adenocarcinoma. Thorac Cancer. 2021;12(18):2468-77. Epub 20210803. doi: 10.1111/1759-7714.14094. PubMed PMID: 34346164; PubMed Central PMCID: PMCPMC8447905.

162. Li PF, Chen SC, Xia T, Jiang XM, Shao YF, Xiao BX, et al. Non-coding RNAs and gastric cancer. World J Gastroenterol. 2014;20(18):5411-9. doi: 10.3748/wjg.v20.i18.5411. PubMed PMID: 24833871; PubMed Central PMCID: PMCPMC4017056.

163. Li W, Gonzalez-Gonzalez M, Sanz-Criado L, Garcia-Carbonero N, Celdran A, Villarejo-Campos P, et al. A Novel PiRNA Enhances CA19-9 Sensitivity for Pancreatic Cancer Identification by Liquid Biopsy. J Clin Med. 2022;11(24). Epub 20221209. doi: 10.3390/jcm11247310. PubMed PMID: 36555927; PubMed Central PMCID: PMCPMC9784851.

164. Li Y, Dong Y, Zhao S, Gao J, Hao X, Wang Z, et al. Serum-derived piR-hsa-164586 of extracellular vesicles as a novel biomarker for early diagnosis of non-small cell lung cancer. Front Oncol. 2022;12:850363. Epub 20220928. doi: 10.3389/fonc.2022.850363. PubMed PMID: 36249068; PubMed Central PMCID: PMCPMC9559724.

165. Li Y, Wu X, Gao H, Jin JM, Li AX, Kim YS, et al. Piwi-Interacting RNAs (piRNAs) Are Dysregulated in Renal Cell Carcinoma and Associated with Tumor Metastasis and Cancer-Specific Survival. Mol Med. 2015;21(1):381-8. Epub 20150513. doi: 10.2119/molmed.2014.00203. PubMed PMID: 25998508; PubMed Central PMCID: PMCPMC4534471.

166. Lim SL, Ricciardelli C, Oehler MK, Tan IM, Russell D, Grützner F. Overexpression of piRNA pathway genes in epithelial ovarian cancer. PLoS One. 2014;9(6):e99687. Epub 20140616. doi: 10.1371/journal.pone.0099687. PubMed PMID: 24932571; PubMed Central PMCID: PMCPMC4059699.

167. Lin X, Lo HC, Wong DT, Xiao X. Noncoding RNAs in human saliva as potential disease biomarkers. Front Genet. 2015;6:175. Epub 20150507. doi: 10.3389/fgene.2015.00175. PubMed PMID: 25999984; PubMed Central PMCID: PMCPMC4423433.

168. Lin Y, Holden V, Dhilipkannah P, Deepak J, Todd NW, Jiang F. A Non-Coding RNA Landscape of Bronchial Epitheliums of Lung Cancer Patients. Biomedicines. 2020;8(4). Epub 20200413. doi: 10.3390/biomedicines8040088. PubMed PMID: 32294932; PubMed Central PMCID: PMCPMC7235744.

169. Lin Y, Zheng J, Lin D. PIWI-interacting RNAs in human cancer. Semin Cancer Biol. 2021;75:15-28. Epub 20200901. doi: 10.1016/j.semcancer.2020.08.012. PubMed PMID: 32877760.

170. Linsen SE, de Wit E, de Bruijn E, Cuppen E. Small RNA expression and strain specificity in the rat. BMC Genomics. 2010;11:249. Epub 20100419. doi: 10.1186/1471-2164-11-249. PubMed PMID: 20403161; PubMed Central PMCID: PMCPMC2864251.

171. Litwin M, Szczepańska-Buda A, Piotrowska A, Dzięgiel P, Witkiewicz W. The meaning of PIWI proteins in cancer development. Oncol Lett. 2017;13(5):3354-62. Epub 20170328. doi: 10.3892/ol.2017.5932. PubMed PMID: 28529570; PubMed Central PMCID: PMCPMC5431467.

172. Liu J, Zhang S, Cheng B. Epigenetic roles of PIWI-interacting RNAs (piRNAs) in cancer metastasis (Review). Oncol Rep. 2018;40(5):2423-34. Epub 20180906. doi: 10.3892/or.2018.6684. PubMed PMID: 30226604.

173. Liu Q, Chen Q, Zhou Z, Tian Z, Zheng X, Wang K. piRNA-18 Inhibition Cell Proliferation, Migration and Invasion in Colorectal Cancer. Biochem Genet. 2023. Epub 20230306. doi: 10.1007/s10528-023-10348-2. PubMed PMID: 36879083.

174. Liu Y, Dong Y, He X, Gong A, Gao J, Hao X, et al. piR-hsa-211106 Inhibits the Progression of Lung Adenocarcinoma Through Pyruvate Carboxylase and Enhances Chemotherapy Sensitivity. Front Oncol. 2021;11:651915. Epub 20210623. doi: 10.3389/fonc.2021.651915. PubMed PMID: 34249688; PubMed Central PMCID: PMCPMC8260943.

175. Liu Y, Dou M, Song X, Dong Y, Liu S, Liu H, et al. The emerging role of the piRNA/piwi complex in cancer. Mol Cancer. 2019;18(1):123. Epub 20190809. doi: 10.1186/s12943-019-1052-9. PubMed PMID: 31399034; PubMed Central PMCID: PMCPMC6688334.

176. Luo Z, Wang X, Jiang H, Wang R, Chen J, Chen Y, et al. Reorganized 3D Genome Structures Support Transcriptional Regulation in Mouse Spermatogenesis. iScience. 2020;23(4):101034. Epub 20200405. doi: 10.1016/j.isci.2020.101034. PubMed PMID: 32315832; PubMed Central PMCID: PMCPMC7170994.

177. Ma D, Zhou X, Wang Y, Dai L, Yuan J, Peng J, et al. Changes in the Small Noncoding RNAome During M1 and M2 Macrophage Polarization. Front Immunol. 2022;13:799733. Epub 20220510. doi: 10.3389/fimmu.2022.799733. PubMed PMID: 35619693; PubMed Central PMCID: PMCPMC9127141.

178. Ma H, Wang H, Tian F, Zhong Y, Liu Z, Liao A. PIWI-Interacting RNA-004800 Is Regulated by S1P Receptor Signaling Pathway to Keep Myeloma Cell Survival. Front Oncol. 2020;10:438. Epub 20200415. doi: 10.3389/fonc.2020.00438. PubMed PMID: 32351883; PubMed Central PMCID: PMCPMC7175921.

179. Maguire S, Lohman GJS, Guan S. A low-bias and sensitive small RNA library preparation method using randomized splint ligation. Nucleic Acids Res. 2020;48(14):e80. doi: 10.1093/nar/gkaa480. PubMed PMID: 32496547; PubMed Central PMCID: PMCPMC7641310.

180. Mahabady MK, Mirzaei S, Saebfar H, Gholami MH, Zabolian A, Hushmandi K, et al. Noncoding RNAs and their therapeutics in paclitaxel chemotherapy: Mechanisms of initiation, progression, and drug sensitivity. J Cell Physiol. 2022;237(5):2309-44. Epub 20220418. doi: 10.1002/jcp.30751. PubMed PMID: 35437787.

181. Mai D, Ding P, Tan L, Zhang J, Pan Z, Bai R, et al. PIWI-interacting RNA-54265 is oncogenic and a potential therapeutic target in colorectal adenocarcinoma. Theranostics. 2018;8(19):5213-30. Epub 20181006. doi: 10.7150/thno.28001. PubMed PMID: 30555542; PubMed Central PMCID: PMCPMC6276099.

182. Mai D, Zheng Y, Guo H, Ding P, Bai R, Li M, et al. Serum piRNA-54265 is a New Biomarker for early detection and clinical surveillance of Human Colorectal Cancer. Theranostics. 2020;10(19):8468-78. Epub 20200709. doi: 10.7150/thno.46241. PubMed PMID: 32754257; PubMed Central PMCID: PMCPMC7392023.

183. Maleki Dana P, Mansournia MA, Mirhashemi SM. PIWI-interacting RNAs: new biomarkers for diagnosis and treatment of breast cancer. Cell Biosci. 2020;10:44. Epub 20200323. doi: 10.1186/s13578-020-00403-5. PubMed PMID: 32211149; PubMed Central PMCID: PMCPMC7092456.

184. Manage KI, Rogers AK, Wallis DC, Uebel CJ, Anderson DC, Nguyen DAH, et al. A tudor domain protein, SIMR-1, promotes siRNA production at piRNA-targeted mRNAs in C. elegans. Elife. 2020;9. Epub 20200427. doi: 10.7554/eLife.56731. PubMed PMID: 32338603; PubMed Central PMCID: PMCPMC7255803.

185. Markert L, Holdmann J, Klinger C, Kaufmann M, Schork K, Turewicz M, et al. Small RNAs as biomarkers to differentiate benign and malign prostate diseases: An alternative for transrectal punch biopsy of the prostate? PLoS One. 2021;16(3):e0247930. Epub 20210324. doi: 10.1371/journal.pone.0247930. PubMed PMID: 33760831; PubMed Central PMCID: PMCPMC7990312.

186. Martinez VD, Firmino NS, Marshall EA, Ng KW, Wadsworth BJ, Anderson C, et al. Non-coding RNAs predict recurrence-free survival of patients with hypoxic tumours. Sci Rep. 2018;8(1):152. Epub 20180109. doi: 10.1038/s41598-017-18462-z. PubMed PMID: 29317756; PubMed Central PMCID: PMCPMC5760628.

187. Martinez VD, Vucic EA, Thu KL, Hubaux R, Enfield KS, Pikor LA, et al. Unique somatic and malignant expression patterns implicate PIWI-interacting RNAs in cancer-type specific biology. Sci Rep. 2015;5:10423. Epub 20150527. doi: 10.1038/srep10423. PubMed PMID: 26013764; PubMed Central PMCID: PMCPMC4444957.

188. Mei Y, Clark D, Mao L. Novel dimensions of piRNAs in cancer. Cancer Lett. 2013;336(1):46-52. Epub 20130416. doi: 10.1016/j.canlet.2013.04.008. PubMed PMID: 23603435; PubMed Central PMCID: PMCPMC3707114.

189. Meng X, Yang S, Zhang Y, Wang X, Goodfellow RX, Jia Y, et al. Genetic Deficiency of Mtdh Gene in Mice Causes Male Infertility via Impaired Spermatogenesis and Alterations in the Expression of Small Non-coding RNAs. J Biol Chem. 2015;290(19):11853-64. Epub 20150318. doi: 10.1074/jbc.M114.627653. PubMed PMID: 25787082; PubMed Central PMCID: PMCPMC4424326.

190. Mentis AA, Dardiotis E, Romas NA, Papavassiliou AG. PIWI family proteins as prognostic markers in cancer: a systematic review and meta-analysis. Cell Mol Life Sci. 2020;77(12):2289-314. Epub 20191209. doi: 10.1007/s00018-019-03403-y. PubMed PMID:

31814070.

191. Miller DF, Yan PS, Buechlein A, Rodriguez BA, Yilmaz AS, Goel S, et al. A new method for stranded whole transcriptome RNA-seq. Methods. 2013;63(2):126-34. Epub 20130401. doi: 10.1016/j.ymeth.2013.03.023. PubMed PMID: 23557989; PubMed Central PMCID: PMCPMC3739992.

192. Minakhina S, Changela N, Steward R. Zfrp8/PDCD2 is required in ovarian stem cells and interacts with the piRNA pathway machinery. Development. 2014;141(2):259-68. doi: 10.1242/dev.101410. PubMed PMID: 24381196; PubMed Central PMCID: PMCPMC3879809.

193. Mokarram P, Niknam M, Sadeghdoust M, Aligholighasemabadi F, Siri M, Dastghaib S, et al. PIWI interacting RNAs perspectives: a new avenues in future cancer investigations. Bioengineered. 2021;12(2):10401-19. doi: 10.1080/21655979.2021.1997078. PubMed PMID: 34723746; PubMed Central PMCID: PMCPMC8809986.

194. Moody SC, Wakitani S, Young JC, Western PS, Loveland KL. Evidence that activin A directly modulates early human male germline differentiation status. Reproduction. 2020;160(1):141-54. doi: 10.1530/rep-20-0095. PubMed PMID: 32484162.

195. Mørup N, Stakaitis R, Golubickaite I, Riera M, Dalgaard MD, Schierup MH, et al. Small RNAs in Seminal Plasma as Novel Biomarkers for Germ Cell Tumors. Cancers (Basel). 2021;13(10). Epub 20210513. doi: 10.3390/cancers13102346. PubMed PMID: 34067956; PubMed Central PMCID: PMCPMC8152278.

196. Mourenza Á, Lorente-Torres B, Durante E, Llano-Verdeja J, Aparicio JF, Fernández-López A, et al. Understanding microRNAs in the Context of Infection to Find New Treatments against Human Bacterial Pathogens. Antibiotics (Basel). 2022;11(3). Epub 20220308. doi: 10.3390/antibiotics11030356. PubMed PMID: 35326819; PubMed Central PMCID: PMCPMC8944844.

197. Munafò M, Lawless VR, Passera A, MacMillan S, Bornelöv S, Haussmann IU, et al. Channel nuclear pore complex subunits are required for transposon silencing in Drosophila. Elife. 2021;10. Epub 20210415. doi: 10.7554/eLife.66321. PubMed PMID: 33856346; PubMed Central PMCID: PMCPMC8133776.

198. Navarro A, Tejero R, Viñolas N, Cordeiro A, Marrades RM, Fuster D, et al. The significance of PIWI family expression in human lung embryogenesis and non-small cell lung cancer. Oncotarget. 2015;6(31):31544-56. doi: 10.18632/oncotarget.3003. PubMed PMID: 25742785; PubMed Central PMCID: PMCPMC4741623.

199. Nayak R, Chattopadhyay T, Gupta P, Mallick B. Integrative analysis of small non-coding RNAs predicts a piRNA/miRNA-CCND1/BRAF/HRH1/ATXN3 regulatory circuit that drives oncogenesis in glioblastoma. Mol Omics. 2023;19(3):252-61. Epub 20230327. doi: 10.1039/d2mo00245k. PubMed PMID: 36688618.

200. Nemoto T, Kakinuma Y. Involvement of Noncoding RNAs in Stress-Related Neuropsychiatric Diseases Caused by DOHaD Theory : ncRNAs and DOHaD-Induced Neuropsychiatric Diseases. Adv Exp Med Biol. 2018;1012:49-59. doi: 10.1007/978-981-10-5526-3_6. PubMed PMID: 29956194.

201. Newkirk SJ, Lee S, Grandi FC, Gaysinskaya V, Rosser JM, Vanden Berg N, et al. Intact piRNA pathway prevents L1 mobilization in male meiosis. Proc Natl Acad Sci U S A. 2017;114(28):E5635-e44. Epub 20170619. doi: 10.1073/pnas.1701069114. PubMed PMID:

28630288; PubMed Central PMCID: PMCPMC5514719.

202. Ng KW, Anderson C, Marshall EA, Minatel BC, Enfield KS, Saprunoff HL, et al. Piwi-interacting RNAs in cancer: emerging functions and clinical utility. Mol Cancer. 2016;15:5. Epub 20160115. doi: 10.1186/s12943-016-0491-9. PubMed PMID: 26768585; PubMed Central PMCID: PMCPMC4714483.

203. Ng L, Navarro A, Law WL. Editorial: Evolving roles of piRNAs in solid tumors. Front Oncol. 2023;13:1178634. Epub 20230411. doi: 10.3389/fonc.2023.1178634. PubMed PMID: 37124490; PubMed Central PMCID: PMCPMC10140357.

204. Nicolas FE. Role of ncRNAs in Development, Diagnosis and Treatment of Human Cancer. Recent Pat Anticancer Drug Discov. 2017;12(2):128-35. doi: 10.2174/1574892812666170105113415. PubMed PMID: 28056753.

205. Nicu AT, Medar C, Chifiriuc MC, Gradisteanu Pircalabioru G, Burlibasa L. Epigenetics and Testicular Cancer: Bridging the Gap Between Fundamental Biology and Patient Care. Front Cell Dev Biol. 2022;10:861995. Epub 20220408. doi: 10.3389/fcell.2022.861995. PubMed PMID: 35465311; PubMed Central PMCID: PMCPMC9023878.

206. Nie Y, Wilson AF, DeFalco T, Meetei AR, Namekawa SH, Pang Q. FANCD2 is required for the repression of germline transposable elements. Reproduction. 2020;159(6):659-68. doi: 10.1530/rep-19-0436. PubMed PMID: 32163912; PubMed Central PMCID: PMCPMC7193181.

207. Nogueira Jorge NA, Wajnberg G, Ferreira CG, de Sa Carvalho B, Passetti F. snoRNA and piRNA expression levels modified by tobacco use in women with lung adenocarcinoma. PLoS One. 2017;12(8):e0183410. Epub 20170817. doi: 10.1371/journal.pone.0183410. PubMed PMID: 28817650; PubMed Central PMCID: PMCPMC5560661.

208. Pall GS, Codony-Servat C, Byrne J, Ritchie L, Hamilton A. Carbodiimide-mediated cross-linking of RNA to nylon membranes improves the detection of siRNA, miRNA and piRNA by northern blot. Nucleic Acids Res. 2007;35(8):e60. Epub 20070402. doi: 10.1093/nar/gkm112. PubMed PMID: 17405769; PubMed Central PMCID: PMCPMC1885651.

209. Pall GS, Hamilton AJ. Improved northern blot method for enhanced detection of small RNA. Nat Protoc. 2008;3(6):1077-84. doi: 10.1038/nprot.2008.67. PubMed PMID: 18536652.

210. Pantano L, Jodar M, Bak M, Ballescà JL, Tommerup N, Oliva R, et al. The small RNA content of human sperm reveals pseudogene-derived piRNAs complementary to protein-coding genes. Rna. 2015;21(6):1085-95. Epub 20150422. doi: 10.1261/rna.046482.114. PubMed PMID: 25904136; PubMed Central PMCID: PMCPMC4436662.

211. Papanikos F, Daniel K, Goercharn-Ramlal A, Fei JF, Kurth T, Wojtasz L, et al. The enigmatic meiotic dense body and its newly discovered component, SCML1, are dispensable for fertility and gametogenesis in mice. Chromosoma. 2017;126(3):399-415. Epub 20160510. doi: 10.1007/s00412-016-0598-1. PubMed PMID: 27165042; PubMed Central PMCID: PMCPMC7326603.

212. Pardini B, Sabo AA, Birolo G, Calin GA. Noncoding RNAs in Extracellular Fluids as Cancer Biomarkers: The New Frontier of Liquid Biopsies. Cancers (Basel). 2019;11(8). Epub 20190814. doi: 10.3390/cancers11081170. PubMed PMID: 31416190; PubMed Central PMCID: PMCPMC6721601.

213. Parkinson J, Wasmuth JD, Salinas G, Bizarro CV, Sanford C, Berriman M, et al. A

transcriptomic analysis of Echinococcus granulosus larval stages: implications for parasite biology and host adaptation. PLoS Negl Trop Dis. 2012;6(11):e1897. Epub 20121129. doi: 10.1371/journal.pntd.0001897. PubMed PMID: 23209850; PubMed Central PMCID: PMCPMC3510090.

214. Parrish NF, Fujino K, Shiromoto Y, Iwasaki YW, Ha H, Xing J, et al. piRNAs derived from ancient viral processed pseudogenes as transgenerational sequence-specific immune memory in mammals. Rna. 2015;21(10):1691-703. Epub 20150817. doi: 10.1261/rna.052092.115. PubMed PMID: 26283688; PubMed Central PMCID: PMCPMC4574747.

215. Pek JW, Kai T. DEAD-box RNA helicase Belle/DDX3 and the RNA interference pathway promote mitotic chromosome segregation. Proc Natl Acad Sci U S A. 2011;108(29):12007-12. Epub 20110705. doi: 10.1073/pnas.1106245108. PubMed PMID: 21730191; PubMed Central PMCID: PMCPMC3141994.

216. Pek JW, Kai T. Non-coding RNAs enter mitosis: functions, conservation and implications. Cell Div. 2011;6:6. Epub 20110228. doi: 10.1186/1747-1028-6-6. PubMed PMID: 21356070; PubMed Central PMCID: PMCPMC3055801.

217. Pekarsky Y, Balatti V, Palamarchuk A, Rizzotto L, Veneziano D, Nigita G, et al. Dysregulation of a family of short noncoding RNAs, tsRNAs, in human cancer. Proc Natl Acad Sci U S A. 2016;113(18):5071-6. Epub 20160411. doi: 10.1073/pnas.1604266113. PubMed PMID: 27071132; PubMed Central PMCID: PMCPMC4983805.

218. Peng JF, Zhuang YY, Huang FT, Zhang SN. Noncoding RNAs and pancreatic cancer. World J Gastroenterol. 2016;22(2):801-14. doi: 10.3748/wjg.v22.i2.801. PubMed PMID: 26811626; PubMed Central PMCID: PMCPMC4716078.

219. Peng Q, Chiu PK, Wong CY, Cheng CK, Teoh JY, Ng CF. Identification of piRNA Targets in Urinary Extracellular Vesicles for the Diagnosis of Prostate Cancer. Diagnostics (Basel). 2021;11(10). Epub 20211003. doi: 10.3390/diagnostics11101828. PubMed PMID: 34679526; PubMed Central PMCID: PMCPMC8534571.

220. Penke TJ, McKay DJ, Strahl BD, Matera AG, Duronio RJ. Direct interrogation of the role of H3K9 in metazoan heterochromatin function. Genes Dev. 2016;30(16):1866-80. Epub 20160826. doi: 10.1101/gad.286278.116. PubMed PMID: 27566777; PubMed Central PMCID: PMCPMC5024684.

221. Perera BPU, Faulk C, Svoboda LK, Goodrich JM, Dolinoy DC. The role of environmental exposures and the epigenome in health and disease. Environ Mol Mutagen. 2020;61(1):176-92. Epub 20190620. doi: 10.1002/em.22311. PubMed PMID: 31177562; PubMed Central PMCID: PMCPMC7252203.

222. Perera BPU, Svoboda L, Dolinoy DC. Genomic Tools for Environmental Epigenetics and Implications for Public Health. Curr Opin Toxicol. 2019;18:27-33. Epub 20190308. doi: 10.1016/j.cotox.2019.02.008. PubMed PMID: 31763499; PubMed Central PMCID: PMCPMC6874218.

223. Pian C, Chen YY, Zhang J, Chen Z, Zhang GL, Li Q, et al. V-ELMpiRNAPred: Identification of human piRNAs by the voting-based extreme learning machine (V-ELM) with a new hybrid feature. J Bioinform Comput Biol. 2017;15(1):1650046. Epub 20170109. doi: 10.1142/s0219720016500463. PubMed PMID: 28178889.

224. Piao HL, Ma L. Non-coding RNAs as regulators of mammary development and breast cancer. J Mammary Gland Biol Neoplasia. 2012;17(1):33-42. Epub 20120217. doi: 10.1007/s10911-012-9245-5. PubMed PMID: 22350981; PubMed Central PMCID: PMCPMC3686545.

225. Pinho JD, Silva GEB, Teixeira-Júnior AAL, Rocha TMS, Batista LL, de Sousa AM, et al. Non-Coding RNA in Penile Cancer. Front Oncol. 2022;12:812008. Epub 20220513. doi: 10.3389/fonc.2022.812008. PubMed PMID: 35651809; PubMed Central PMCID: PMCPMC9150447.

226. Pinto P, da Silva MB, Moreira FC, Bouth RC, Gobbo AR, Sandoval TV, et al. Leprosy piRnome: exploring new possibilities for an old disease. Sci Rep. 2020;10(1):12648. Epub 20200728. doi: 10.1038/s41598-020-69355-7. PubMed PMID: 32724108; PubMed Central PMCID: PMCPMC7387468.

227. Pöyhönen M, de Vanssay A, Delmarre V, Hermant C, Todeschini AL, Teysset L, et al. Homology-dependent silencing by an exogenous sequence in the Drosophila germline. G3 (Bethesda). 2012;2(3):331-8. Epub 20120301. doi: 10.1534/g3.111.001925. PubMed PMID: 22413086; PubMed Central PMCID: PMCPMC3291502.

228. Qian L, Xie H, Zhang L, Zhao Q, Lü J, Yu Z. Piwi-Interacting RNAs: A New Class of Regulator in Human Breast Cancer. Front Oncol. 2021;11:695077. Epub 20210706. doi: 10.3389/fonc.2021.695077. PubMed PMID: 34295823; PubMed Central PMCID: PMCPMC8290475.

229. Qiu W, Guo X, Lin X, Yang Q, Zhang W, Zhang Y, et al. Transcriptome-wide piRNA profiling in human brains of Alzheimer's disease. Neurobiol Aging. 2017;57:170-7. Epub 20170603. doi: 10.1016/j.neurobiolaging.2017.05.020. PubMed PMID: 28654860; PubMed Central PMCID: PMCPMC5542056.

230. Qu A, Wang W, Yang Y, Zhang X, Dong Y, Zheng G, et al. A serum piRNA signature as promising non-invasive diagnostic and prognostic biomarkers for colorectal cancer. Cancer Manag Res. 2019;11:3703-20. Epub 20190429. doi: 10.2147/cmar.S193266. PubMed PMID: 31118791; PubMed Central PMCID: PMCPMC6500438.

231. Rahimy E, Kuo SZ, Ongkeko WM. Evaluation of non-coding RNAs as potential targets in head and neck squamous cell carcinoma cancer stem cells. Curr Drug Targets. 2014;15(13):1247-60. doi: 10.2174/1389450115666141024113446. PubMed PMID: 25341422.

232. Ramakrishna NB, Battistoni G, Surani MA, Hannon GJ, Miska EA. Mouse primordial germ-cell-like cells lack piRNAs. Dev Cell. 2022;57(23):2661-8.e5. doi: 10.1016/j.devcel.2022.11.004. PubMed PMID: 36473462.

233. Ravo M, Cordella A, Rinaldi A, Bruno G, Alexandrova E, Saggese P, et al. Small non-coding RNA deregulation in endometrial carcinogenesis. Oncotarget. 2015;6(7):4677-91. doi: 10.18632/oncotarget.2911. PubMed PMID: 25686835; PubMed Central PMCID: PMCPMC4467107.

234. Ray SK, Mukherjee S. Piwi-interacting RNAs (piRNAs) and Colorectal Carcinoma: Emerging Non-invasive diagnostic Biomarkers with Potential Therapeutic Target Based Clinical Implications. Curr Mol Med. 2023;23(4):300-11. doi: 10.2174/1566524022666220124102616. PubMed PMID: 35068393.

235. Rayford KJ, Cooley A, Rumph JT, Arun A, Rachakonda G, Villalta F, et al. piRNAs as Modulators of Disease Pathogenesis. Int J Mol Sci. 2021;22(5). Epub 20210227. doi:

10.3390/ijms22052373. PubMed PMID: 33673453; PubMed Central PMCID: PMCPMC7956838.

236. Raza A, Khan AQ, Inchakalody VP, Mestiri S, Yoosuf Z, Bedhiafi T, et al. Dynamic liquid biopsy components as predictive and prognostic biomarkers in colorectal cancer. J Exp Clin Cancer Res. 2022;41(1):99. Epub 20220315. doi: 10.1186/s13046-022-02318-0. PubMed PMID: 35292091; PubMed Central PMCID: PMCPMC8922757.

237. Razavi ZS, Tajiknia V, Majidi S, Ghandali M, Mirzaei HR, Rahimian N, et al. Gynecologic cancers and non-coding RNAs: Epigenetic regulators with emerging roles. Crit Rev Oncol Hematol. 2021;157:103192. Epub 20201205. doi: 10.1016/j.critrevonc.2020.103192. PubMed PMID: 33290823.

238. Reddy HM, Bhattacharya R, Tiwari S, Mishra K, Annapurna P, Jehan Z, et al. Y chromosomal noncoding RNAs regulate autosomal gene expression via piRNAs in mouse testis. BMC Biol. 2021;19(1):198. Epub 20210909. doi: 10.1186/s12915-021-01125-x. PubMed PMID: 34503492; PubMed Central PMCID: PMCPMC8428117.

239. Riedmann LT, Schwentner R. miRNA, siRNA, piRNA and argonautes: news in small matters. RNA Biol. 2010;7(2):133-9. Epub 20100325. doi: 10.4161/rna.7.2.11288. PubMed PMID: 20200493.

240. Riquelme I, Pérez-Moreno P, Letelier P, Brebi P, Roa JC. The Emerging Role of PIWI-Interacting RNAs (piRNAs) in Gastrointestinal Cancers: An Updated Perspective. Cancers (Basel). 2021;14(1). Epub 20211231. doi: 10.3390/cancers14010202. PubMed PMID: 35008366; PubMed Central PMCID: PMCPMC8750603.

241. Ritter A, Hirschfeld M, Berner K, Rücker G, Jäger M, Weiss D, et al. Circulating non-coding RNA-biomarker potential in neoadjuvant chemotherapy of triple negative breast cancer? Int J Oncol. 2020;56(1):47-68. Epub 20191125. doi: 10.3892/ijo.2019.4920. PubMed PMID: 31789396; PubMed Central PMCID: PMCPMC6910196.

242. Rizzo F, Rinaldi A, Marchese G, Coviello E, Sellitto A, Cordella A, et al. Specific patterns of PIWI-interacting small noncoding RNA expression in dysplastic liver nodules and hepatocellular carcinoma. Oncotarget. 2016;7(34):54650-61. doi: 10.18632/oncotarget.10567. PubMed PMID: 27429044; PubMed Central PMCID: PMCPMC5342370.

243. Romano G, Veneziano D, Acunzo M, Croce CM. Small non-coding RNA and cancer. Carcinogenesis. 2017;38(5):485-91. doi: 10.1093/carcin/bgx026. PubMed PMID: 28449079; PubMed Central PMCID: PMCPMC6248440.

244. Ross RJ, Weiner MM, Lin H. PIWI proteins and PIWI-interacting RNAs in the soma. Nature. 2014;505(7483):353-9. doi: 10.1038/nature12987. PubMed PMID: 24429634; PubMed Central PMCID: PMCPMC4265809.

245. Rounge TB, Furu K, Skotheim RI, Haugen TB, Grotmol T, Enerly E. Profiling of the small RNA populations in human testicular germ cell tumors shows global loss of piRNAs. Mol Cancer. 2015;14:153. Epub 20150812. doi: 10.1186/s12943-015-0411-4. PubMed PMID: 26265322; PubMed Central PMCID: PMCPMC4533958.

246. Rounge TB, Umu SU, Keller A, Meese E, Ursin G, Tretli S, et al. Circulating small non-coding RNAs associated with age, sex, smoking, body mass and physical activity. Sci Rep. 2018;8(1):17650. Epub 20181205. doi: 10.1038/s41598-018-35974-4. PubMed PMID: 30518766; PubMed Central PMCID: PMCPMC6281647.

247. Roy J, Das B, Jain N, Mallick B. PIWI-interacting RNA 39980 promotes tumor progression and reduces drug sensitivity in neuroblastoma cells. J Cell Physiol. 2020;235(3):2286-99. Epub 20190903. doi: 10.1002/jcp.29136. PubMed PMID: 31478570.

248. Roy R, Pattnaik S, Sivagurunathan S, Chidambaram S. Small ncRNA binding protein, PIWI: A potential molecular bridge between blood brain barrier and neuropathological conditions. Med Hypotheses. 2020;138:109609. Epub 20200131. doi: 10.1016/j.mehy.2020.109609. PubMed PMID: 32070788.

249. Royo H, Cavaillé J. Non-coding RNAs in imprinted gene clusters. Biol Cell. 2008;100(3):149-66. doi: 10.1042/bc20070126. PubMed PMID: 18271756.

250. Rui T, Wang K, Xiang A, Guo J, Tang N, Jin X, et al. Serum Exosome-Derived piRNAs Could Be Promising Biomarkers for HCC Diagnosis. Int J Nanomedicine. 2023;18:1989-2001. Epub 20230413. doi: 10.2147/ijn.S398462. PubMed PMID: 37077942; PubMed Central PMCID: PMCPMC10108868.

251. Russell S, Patel M, Gilchrist G, Stalker L, Gillis D, Rosenkranz D, et al. Bovine piRNA-like RNAs are associated with both transposable elements and mRNAs. Reproduction. 2017;153(3):305-18. Epub 20161213. doi: 10.1530/rep-16-0620. PubMed PMID: 27965401.

252. Rybecká S, Štítkovcová K, Vychytilová-Faltejsková P, Slabý O. [Involvement of PIWI-interacting RNAs in Cancerogenesis via the Regulation of Gene Expression]. Klin Onkol. 2016;29(6):428-38. doi: 10.14735/amko2016428. PubMed PMID: 27951720.

253. Sabbah NA, Abdalla WM, Mawla WA, AbdAlMonem N, Gharib AF, Abdul-Saboor A, et al. piRNA-823 Is a Unique Potential Diagnostic Non-Invasive Biomarker in Colorectal Cancer Patients. Genes (Basel). 2021;12(4). Epub 20210419. doi: 10.3390/genes12040598. PubMed PMID: 33921704; PubMed Central PMCID: PMCPMC8074037.

254. Sabeena S. Role of noncoding RNAs with emphasis on long noncoding RNAs as cervical cancer biomarkers. J Med Virol. 2023;95(2):e28525. doi: 10.1002/jmv.28525. PubMed PMID: 36702772.

255. Sabo AA, Birolo G, Naccarati A, Dragomir MP, Aneli S, Allione A, et al. Small Non-Coding RNA Profiling in Plasma Extracellular Vesicles of Bladder Cancer Patients by Next-Generation Sequencing: Expression Levels of miR-126-3p and piR-5936 Increase with Higher Histologic Grades. Cancers (Basel). 2020;12(6). Epub 20200609. doi: 10.3390/cancers12061507. PubMed PMID: 32527011; PubMed Central PMCID: PMCPMC7352804.

256. Sadovska L, Zayakin P, Eglītis K, Endzeliņš E, Radoviča-Spalviņa I, Avotiņa E, et al. Comprehensive characterization of RNA cargo of extracellular vesicles in breast cancer patients undergoing neoadjuvant chemotherapy. Front Oncol. 2022;12:1005812. Epub 20221026. doi: 10.3389/fonc.2022.1005812. PubMed PMID: 36387168; PubMed Central PMCID: PMCPMC9644097.

257. Saritas G, Main AM, Winge SB, Mørup N, Almstrup K. PIWI-interacting RNAs and human testicular function. WIREs Mech Dis. 2022;14(6):e1572. Epub 20220719. doi: 10.1002/wsbm.1572. PubMed PMID: 35852002; PubMed Central PMCID: PMCPMC9788060.

258. Sarraf JS, Puty TC, da Silva EM, Allen TSR, Sarraf YS, de Carvalho LEW, et al. Noncoding RNAs and Colorectal Cancer: A General Overview. Microrna. 2020;9(5):336-45. doi: 10.2174/2211536609666201221124608. PubMed PMID: 33349228.

259. Sato K, Nishida KM, Shibuya A, Siomi MC, Siomi H. Maelstrom coordinates microtubule

organization during Drosophila oogenesis through interaction with components of the MTOC. Genes Dev. 2011;25(22):2361-73. doi: 10.1101/gad.174110.111. PubMed PMID: 22085963; PubMed Central PMCID: PMCPMC3222902.

260. Sato K, Siomi MC. Piwi-interacting RNAs: biological functions and biogenesis. Essays Biochem. 2013;54:39-52. doi: 10.1042/bse0540039. PubMed PMID: 23829526.

261. Schulze M, Sommer A, Plötz S, Farrell M, Winner B, Grosch J, et al. Sporadic Parkinson's disease derived neuronal cells show disease-specific mRNA and small RNA signatures with abundant deregulation of piRNAs. Acta Neuropathol Commun. 2018;6(1):58. Epub 20180710. doi: 10.1186/s40478-018-0561-x. PubMed PMID: 29986767; PubMed Central PMCID: PMCPMC6038190.

262. Serafini MS, Lopez-Perez L, Fico G, Licitra L, De Cecco L, Resteghini C. Transcriptomics and Epigenomics in head and neck cancer: available repositories and molecular signatures. Cancers Head Neck. 2020;5:2. Epub 20200121. doi: 10.1186/s41199-020-0047-y. PubMed PMID: 31988797; PubMed Central PMCID: PMCPMC6971871.

263. Seyeddokht A, Aslaminejad AA, Masoudi-Nejad A, Nassiri M, Zahiri J, Sadeghi B. Computational Detection of piRNA in Human Using Support Vector Machine. Avicenna J Med Biotechnol. 2016;8(1):36-41. PubMed PMID: 26855734; PubMed Central PMCID: PMCPMC4717465.

264. Shah AA, Leidinger P, Blin N, Meese E. miRNA: small molecules as potential novel biomarkers in cancer. Curr Med Chem. 2010;17(36):4427-32. doi: 10.2174/092986710794182980. PubMed PMID: 21062260.

265. Shi S, Yang ZZ, Liu S, Yang F, Lin H. PIWIL1 promotes gastric cancer via a piRNA-independent mechanism. Proc Natl Acad Sci U S A. 2020;117(36):22390-401. Epub 20200826. doi: 10.1073/pnas.2008724117. PubMed PMID: 32848063; PubMed Central PMCID: PMCPMC7486755.

266. Shi X, Xiao Z, Zonta F, Wang W, Wan Y, Li Y, et al. Somatic MIWI2 Hinders Direct Lineage Reprogramming From Fibroblast to Hepatocyte. Stem Cells. 2019;37(6):803-12. Epub 20190228. doi: 10.1002/stem.2994. PubMed PMID: 30805989; PubMed Central PMCID: PMCPMC6850183.

267. Shirzad H. PiRNA Biogenesis and Their Role in Human Cancers and Other Diseases: A Narrative Review. Iran J Public Health. 2021;50(12):2486-94. doi: 10.18502/ijph.v50i12.7930. PubMed PMID: 36317021; PubMed Central PMCID: PMCPMC9577162.

268. Siddiqi S, Matushansky I. Piwis and piwi-interacting RNAs in the epigenetics of cancer. J Cell Biochem. 2012;113(2):373-80. doi: 10.1002/jcb.23363. PubMed PMID: 21928326.

269. Singh G, Roy J, Rout P, Mallick B. Genome-wide profiling of the PIWI-interacting RNA-mRNA regulatory networks in epithelial ovarian cancers. PLoS One. 2018;13(1):e0190485. Epub 20180110. doi: 10.1371/journal.pone.0190485. PubMed PMID: 29320577; PubMed Central PMCID: PMCPMC5761873.

270. Singh RS, Arna AB, Dong H, Yadav M, Aggarwal A, Wu Y. Structure-function analysis of DEAD-box helicase DDX43. Methods. 2022;204:286-99. Epub 20220304. doi: 10.1016/j.ymeth.2022.03.002. PubMed PMID: 35257897.

271. Sioud M. RNA Interference: Story and Mechanisms. Methods Mol Biol. 2021;2282:1-15. doi: 10.1007/978-1-0716-1298-9_1. PubMed PMID: 33928566.

272. Slaby O. Non-coding RNAs as Biomarkers for Colorectal Cancer Screening and Early Detection. Adv Exp Med Biol. 2016;937:153-70. doi: 10.1007/978-3-319-42059-2_8. PubMed PMID: 27573899.

273. Sohn EJ, Oh SO. P-Element-Induced Wimpy Testis Proteins and P-Element-Induced Wimpy Testis-Interacting RNAs Expression in Ovarian Cancer Stem Cells. Genet Test Mol Biomarkers. 2023;27(2):56-64. doi: 10.1089/gtmb.2022.0113. PubMed PMID: 36853842.

274. Sokolova MI, Zelentsova ES, Shostak NG, Rozhkov NV, Evgen'ev MB. Ontogenetic consequences of dysgenic crosses in Drosophila virilis. Int J Dev Biol. 2013;57(9-10):731-9. doi: 10.1387/ijdb.120189me. PubMed PMID: 24395561.

275. Sokolova OA, Mikhaleva EA, Kharitonov SL, Abramov YA, Gvozdev VA, Klenov MS. Special vulnerability of somatic niche cells to transposable element activation in Drosophila larval ovaries. Sci Rep. 2020;10(1):1076. Epub 20200123. doi: 10.1038/s41598-020-57901-2. PubMed PMID: 31974416; PubMed Central PMCID: PMCPMC6978372.

276. Sole C, Arnaiz E, Manterola L, Otaegui D, Lawrie CH. The circulating transcriptome as a source of cancer liquid biopsy biomarkers. Semin Cancer Biol. 2019;58:100-8. Epub 20190123. doi: 10.1016/j.semcancer.2019.01.003. PubMed PMID: 30684535.

277. Sonea L, Buse M, Gulei D, Onaciu A, Simon I, Braicu C, et al. Decoding the Emerging Patterns Exhibited in Non-coding RNAs Characteristic of Lung Cancer with Regard to their Clinical Significance. Curr Genomics. 2018;19(4):258-78. doi: 10.2174/1389202918666171005100124. PubMed PMID: 29755289; PubMed Central PMCID: PMCPMC5930448.

278. Su JF, Zhao F, Gao ZW, Hou YJ, Li YY, Duan LJ, et al. piR-823 demonstrates tumor oncogenic activity in esophageal squamous cell carcinoma through DNA methylation induction via DNA methyltransferase 3B. Pathol Res Pract. 2020;216(4):152848. Epub 20200205. doi: 10.1016/j.prp.2020.152848. PubMed PMID: 32051106.

279. Subhramanyam CS, Cao Q, Wang C, Heng ZSL, Zhou Z, Hu Q. Role of PIWI-like 4 in modulating neuronal differentiation from human embryonal carcinoma cells. RNA Biol. 2020;17(11):1613-24. Epub 20200506. doi: 10.1080/15476286.2020.1757896. PubMed PMID: 32372724; PubMed Central PMCID: PMCPMC7567516.

280. Suen KM, Braukmann F, Butler R, Bensaddek D, Akay A, Lin CC, et al. DEPS-1 is required for piRNA-dependent silencing and PIWI condensate organisation in Caenorhabditis elegans. Nat Commun. 2020;11(1):4242. Epub 20200825. doi: 10.1038/s41467-020-18089-1. PubMed PMID: 32843637; PubMed Central PMCID: PMCPMC7447803.

281. Sumiyoshi T, Sato K, Yamamoto H, Iwasaki YW, Siomi H, Siomi MC. Loss of l(3)mbt leads to acquisition of the ping-pong cycle in Drosophila ovarian somatic cells. Genes Dev. 2016;30(14):1617-22. doi: 10.1101/gad.283929.116. PubMed PMID: 27474440; PubMed Central PMCID: PMCPMC4973291.

282. Sun CY, Chen GD, He BC, Fu WE, Lee CH, Leu YW, et al. Dysregulated HIC1 and RassF1A expression in vitro alters the cell cytoskeleton and exosomal Piwi-interacting RNA. Biochem Biophys Res Commun. 2022;594:109-16. Epub 20220119. doi: 10.1016/j.bbrc.2022.01.065. PubMed PMID: 35081499.

283. Szilágyi M, Pös O, Márton É, Buglyó G, Soltész B, Keserű J, et al. Circulating Cell-Free Nucleic Acids: Main Characteristics and Clinical Application. Int J Mol Sci. 2020;21(18). Epub

20200917. doi: 10.3390/ijms21186827. PubMed PMID: 32957662; PubMed Central PMCID: PMCPMC7555669.

284. Taft RJ, Pang KC, Mercer TR, Dinger M, Mattick JS. Non-coding RNAs: regulators of disease. J Pathol. 2010;220(2):126-39. doi: 10.1002/path.2638. PubMed PMID: 19882673.

285. Tamtaji OR, Behnam M, Pourattar MA, Hamblin MR, Mahjoubin-Tehran M, Mirzaei H, et al. PIWI-interacting RNAs and PIWI proteins in glioma: molecular pathogenesis and role as biomarkers. Cell Commun Signal. 2020;18(1):168. Epub 20201027. doi: 10.1186/s12964-020-00657-z. PubMed PMID: 33109195; PubMed Central PMCID: PMCPMC7590611.

286. Tan C, Cao J, Chen L, Xi X, Wang S, Zhu Y, et al. Noncoding RNAs Serve as Diagnosis and Prognosis Biomarkers for Hepatocellular Carcinoma. Clin Chem. 2019;65(7):905-15. Epub 20190417. doi: 10.1373/clinchem.2018.301150. PubMed PMID: 30996051.

287. Tan L, Mai D, Zhang B, Jiang X, Zhang J, Bai R, et al. PIWI-interacting RNA-36712 restrains breast cancer progression and chemoresistance by interaction with SEPW1 pseudogene SEPW1P RNA. Mol Cancer. 2019;18(1):9. Epub 20190112. doi: 10.1186/s12943-019-0940-3. PubMed PMID: 30636640; PubMed Central PMCID: PMCPMC6330501.

288. Tang F, Hayashi K, Kaneda M, Lao K, Surani MA. A sensitive multiplex assay for piRNA expression. Biochem Biophys Res Commun. 2008;369(4):1190-4. Epub 20080317. doi: 10.1016/j.bbrc.2008.03.035. PubMed PMID: 18348866; PubMed Central PMCID: PMCPMC3855189.

289. Telkar N, Stewart GL, Pewarchuk ME, Cohn DE, Robinson WP, Lam WL. Small Non-Coding RNAs in the Human Placenta: Regulatory Roles and Clinical Utility. Front Genet. 2022;13:868598. Epub 20220330. doi: 10.3389/fgene.2022.868598. PubMed PMID: 35432451; PubMed Central PMCID: PMCPMC9006164.

290. Teplyakov E, Wu Q, Liu J, Pugacheva EM, Loukinov D, Boukaba A, et al. The downregulation of putative anticancer target BORIS/CTCFL in an addicted myeloid cancer cell line modulates the expression of multiple protein coding and ncRNA genes. Oncotarget. 2017;8(43):73448-68. Epub 20170902. doi: 10.18632/oncotarget.20627. PubMed PMID: 29088719; PubMed Central PMCID: PMCPMC5650274.

291. Tian Y, Simanshu DK, Ma JB, Patel DJ. Structural basis for piRNA 2'-O-methylated 3'-end recognition by Piwi PAZ (Piwi/Argonaute/Zwille) domains. Proc Natl Acad Sci U S A. 2011;108(3):903-10. Epub 20101230. doi: 10.1073/pnas.1017762108. PubMed PMID: 21193640; PubMed Central PMCID: PMCPMC3024652.

292. Toden S, Zumwalt TJ, Goel A. Non-coding RNAs and potential therapeutic targeting in cancer. Biochim Biophys Acta Rev Cancer. 2021;1875(1):188491. Epub 20201213. doi: 10.1016/j.bbcan.2020.188491. PubMed PMID: 33316377; PubMed Central PMCID: PMCPMC7856203.

293. Tong Y, Guan B, Sun Z, Dong X, Chen Y, Li Y, et al. Ratiometric fluorescent detection of exosomal piRNA-823 based on Au NCs/UiO-66-NH(2) and target-triggered rolling circle amplification. Talanta. 2023;257:124307. Epub 20230204. doi: 10.1016/j.talanta.2023.124307. PubMed PMID: 36764170.

294. Tosar JP, García-Silva MR, Cayota A. Circulating SNORD57 rather than piR-54265 is a promising biomarker for colorectal cancer: common pitfalls in the study of somatic piRNAs in cancer. Rna. 2021;27(4):403-10. Epub 20201229. doi: 10.1261/rna.078444.120. PubMed

PMID: 33376191; PubMed Central PMCID: PMCPMC7962485.

295. Trzybulska D, Vergadi E, Tsatsanis C. miRNA and Other Non-Coding RNAs as Promising Diagnostic Markers. Ejifcc. 2018;29(3):221-6. Epub 20181107. PubMed PMID: 30479608; PubMed Central PMCID: PMCPMC6247131.

296. Umu SU, Langseth H, Bucher-Johannessen C, Fromm B, Keller A, Meese E, et al. A comprehensive profile of circulating RNAs in human serum. RNA Biol. 2018;15(2):242-50. Epub 20171208. doi: 10.1080/15476286.2017.1403003. PubMed PMID: 29219730; PubMed Central PMCID: PMCPMC5798962.

297. Vafaei S, Fattahi F, Sahlolbei M, Kiani J, Yazdanpanah A, Madjd Z. Dynamic Signature of tRNA-Derived Small RNAs in Cancer Pathogenesis as a Promising Valuable Approach. Crit Rev Eukaryot Gene Expr. 2020;30(5):391-410. doi: 10.1615/CritRevEukaryotGeneExpr.2020035372. PubMed PMID: 33389877.

298. Vallone C, Rigon G, Gulia C, Baffa A, Votino R, Morosetti G, et al. Non-Coding RNAs and Endometrial Cancer. Genes (Basel). 2018;9(4). Epub 20180329. doi: 10.3390/genes9040187. PubMed PMID: 29596364; PubMed Central PMCID: PMCPMC5924529.

299. Venkatesh T, Suresh PS, Tsutsumi R. Non-coding RNAs: Functions and applications in endocrine-related cancer. Mol Cell Endocrinol. 2015;416:88-96. Epub 20150907. doi: 10.1016/j.mce.2015.08.026. PubMed PMID: 26360585.

300. Vinasco-Sandoval T, Moreira FC, A FV, Pinto P, Ribeiro-Dos-Santos AM, Cruz RLS, et al. Global Analyses of Expressed Piwi-Interacting RNAs in Gastric Cancer. Int J Mol Sci. 2020;21(20). Epub 20201016. doi: 10.3390/ijms21207656. PubMed PMID: 33081152; PubMed Central PMCID: PMCPMC7593925.

301. Vychytilova-Faltejskova P, Stitkovcova K, Radova L, Sachlova M, Kosarova Z, Slaba K, et al. Circulating PIWI-Interacting RNAs piR-5937 and piR-28876 Are Promising Diagnostic Biomarkers of Colon Cancer. Cancer Epidemiol Biomarkers Prev. 2018;27(9):1019-28. Epub 20180705. doi: 10.1158/1055-9965.Epi-18-0318. PubMed PMID: 29976566.

302. Wajahat M, Bracken CP, Orang A. Emerging Functions for snoRNAs and snoRNA-Derived Fragments. Int J Mol Sci. 2021;22(19). Epub 20210922. doi: 10.3390/ijms221910193. PubMed PMID: 34638533; PubMed Central PMCID: PMCPMC8508363.

303. Wang H, Shi B, Zhang X, Shen P, He Q, Yin M, et al. Exosomal hsa-piR1089 promotes proliferation and migration in neuroblastoma via targeting KEAP1. Pathol Res Pract. 2023;241:154240. Epub 20221123. doi: 10.1016/j.prp.2022.154240. PubMed PMID: 36436316.

304. Wang J, Song YX, Ma B, Wang JJ, Sun JX, Chen XW, et al. Regulatory Roles of Non-Coding RNAs in Colorectal Cancer. Int J Mol Sci. 2015;16(8):19886-919. Epub 20150821. doi: 10.3390/ijms160819886. PubMed PMID: 26307974; PubMed Central PMCID: PMCPMC4581331.

305. Wang K, Wang T, Gao XQ, Chen XZ, Wang F, Zhou LY. Emerging functions of piwi-interacting RNAs in diseases. J Cell Mol Med. 2021;25(11):4893-901. Epub 20210504. doi: 10.1111/jcmm.16466. PubMed PMID: 33942984; PubMed Central PMCID: PMCPMC8178273.

306. Wang X, Gou LT, Liu MF. Noncanonical functions of PIWIL1/piRNAs in animal male germ cells and human diseases†. Biol Reprod. 2022;107(1):101-8. doi: 10.1093/biolre/ioac073.

PubMed PMID: 35403682.

307. Wang Y, Gable T, Ma MZ, Clark D, Zhao J, Zhang Y, et al. A piRNA-like Small RNA Induces Chemoresistance to Cisplatin-Based Therapy by Inhibiting Apoptosis in Lung Squamous Cell Carcinoma. Mol Ther Nucleic Acids. 2017;6:269-78. Epub 20170124. doi: 10.1016/j.omtn.2017.01.003. PubMed PMID: 28325293; PubMed Central PMCID: PMCPMC5363509.

308. Wang Z, Yang H, Ma D, Mu Y, Tan X, Hao Q, et al. Serum PIWI-Interacting RNAs piR-020619 and piR-020450 Are Promising Novel Biomarkers for Early Detection of Colorectal Cancer. Cancer Epidemiol Biomarkers Prev. 2020;29(5):990-8. Epub 20200217. doi: 10.1158/1055-9965.Epi-19-1148. PubMed PMID: 32066615.

309. Wang ZL, Zhang XQ, Zhou H, Yang JH, Qu LH. oncoNcRNA: A Web Portal for Exploring the Non-Coding RNAs with Oncogenic Potentials in Human Cancers. Noncoding RNA. 2017;3(1). Epub 20170207. doi: 10.3390/ncrna3010007. PubMed PMID: 29657279; PubMed Central PMCID: PMCPMC5832004.

310. Wang ZY, Wen ZJ, Xu HM, Zhang Y, Zhang YF. Exosomal noncoding RNAs in central nervous system diseases: biological functions and potential clinical applications. Front Mol Neurosci. 2022;15:1004221. Epub 20221109. doi: 10.3389/fnmol.2022.1004221. PubMed PMID: 36438184; PubMed Central PMCID: PMCPMC9681831.

311. Weick EM, Sarkies P, Silva N, Chen RA, Moss SM, Cording AC, et al. PRDE-1 is a nuclear factor essential for the biogenesis of Ruby motif-dependent piRNAs in C. elegans. Genes Dev. 2014;28(7):783-96. doi: 10.1101/gad.238105.114. PubMed PMID: 24696457; PubMed Central PMCID: PMCPMC4015492.

312. Weng W, Li H, Goel A. Piwi-interacting RNAs (piRNAs) and cancer: Emerging biological concepts and potential clinical implications. Biochim Biophys Acta Rev Cancer. 2019;1871(1):160-9. Epub 20181230. doi: 10.1016/j.bbcan.2018.12.005. PubMed PMID: 30599187; PubMed Central PMCID: PMCPMC6392428.

313. Weng W, Liu N, Toiyama Y, Kusunoki M, Nagasaka T, Fujiwara T, et al. Novel evidence for a PIWI-interacting RNA (piRNA) as an oncogenic mediator of disease progression, and a potential prognostic biomarker in colorectal cancer. Mol Cancer. 2018;17(1):16. Epub 20180130. doi: 10.1186/s12943-018-0767-3. PubMed PMID: 29382334; PubMed Central PMCID: PMCPMC5791351.

314. Wichman L, Somasundaram S, Breindel C, Valerio DM, McCarrey JR, Hodges CA, et al. Dynamic expression of long noncoding RNAs reveals their potential roles in spermatogenesis and fertility. Biol Reprod. 2017;97(2):313-23. doi: 10.1093/biolre/iox084. PubMed PMID: 29044429.

315. Wu D, Fu H, Zhou H, Su J, Zhang F, Shen J. Effects of Novel ncRNA Molecules, p15-piRNAs, on the Methylation of DNA and Histone H3 of the CDKN2B Promoter Region in U937 Cells. J Cell Biochem. 2015;116(12):2744-54. doi: 10.1002/jcb.25199. PubMed PMID: 26205624.

316. Wu L, Jiang Y, Zheng Z, Li H, Cai M, Pathak JL, et al. mRNA and P-element-induced wimpy testis-interacting RNA profile in chemical-induced oral squamous cell carcinoma mice model. Exp Anim. 2020;69(2):168-77. Epub 20191119. doi: 10.1538/expanim.19-0042. PubMed PMID: 31748426; PubMed Central PMCID: PMCPMC7220707.

317. Wu Q, Ma Q, Shehadeh LA, Wilson A, Xia L, Yu H, et al. Expression of the Argonaute protein

PiwiL2 and piRNAs in adult mouse mesenchymal stem cells. Biochem Biophys Res Commun. 2010;396(4):915-20. Epub 20100510. doi: 10.1016/j.bbrc.2010.05.022. PubMed PMID: 20460113; PubMed Central PMCID: PMCPMC3151571.

318. Wu X, Pan Y, Fang Y, Zhang J, Xie M, Yang F, et al. The Biogenesis and Functions of piRNAs in Human Diseases. Mol Ther Nucleic Acids. 2020;21:108-20. Epub 20200523. doi: 10.1016/j.omtn.2020.05.023. PubMed PMID: 32516734; PubMed Central PMCID: PMCPMC7283962.

319. Xiao L, Wang J, Ju S, Cui M, Jing R. Disorders and roles of tsRNA, snoRNA, snRNA and piRNA in cancer. J Med Genet. 2022;59(7):623-31. Epub 20220210. doi: 10.1136/jmedgenet-2021-108327. PubMed PMID: 35145038.

320. Xie S, Chang Y, Jin H, Yang F, Xu Y, Yan X, et al. Non-coding RNAs in gastric cancer. Cancer Lett. 2020;493:55-70. Epub 20200723. doi: 10.1016/j.canlet.2020.06.022. PubMed PMID: 32712234.

321. Xie W, Sowemimo I, Hayashi R, Wang J, Burkard TR, Brennecke J, et al. Structure-function analysis of microRNA 3'-end trimming by Nibbler. Proc Natl Acad Sci U S A. 2020;117(48):30370-9. Epub 20201116. doi: 10.1073/pnas.2018156117. PubMed PMID: 33199607; PubMed Central PMCID: PMCPMC7720153.

322. Xin J, Du M, Jiang X, Wu Y, Ben S, Zheng R, et al. Systematic evaluation of the effects of genetic variants on PIWI-interacting RNA expression across 33 cancer types. Nucleic Acids Res. 2021;49(1):90-7. doi: 10.1093/nar/gkaa1190. PubMed PMID: 33330918; PubMed Central PMCID: PMCPMC7797066.

323. Xin XL, Wang GD, Han R, Jiang Y, Liu C, Liu LS, et al. Mechanism underlying the effect of Liujunzi decoction on advanced-stage non-small cell lung cancer in patients after first-line chemotherapy. J Tradit Chin Med. 2022;42(1):108-15. PubMed PMID: 35294130.

324. Xiong Q, Zhang Y, Li J, Zhu Q. Small Non-Coding RNAs in Human Cancer. Genes (Basel). 2022;13(11). Epub 20221109. doi: 10.3390/genes13112072. PubMed PMID: 36360311; PubMed Central PMCID: PMCPMC9690286.

325. Xu J, Yang X, Zhou Q, Zhuang J, Han S. Biological significance of piRNA in liver cancer: a review. Biomarkers. 2020;25(6):436-40. Epub 20200720. doi: 10.1080/1354750x.2020.1794041. PubMed PMID: 32662667.

326. Xu Z, Ji G, Cui Y, Cui X. The Impacts of Non-coding RNAs and N(6)-Methyladenosine on Cancer: Past, Present and Future. Curr Cancer Drug Targets. 2021;21(5):375-85. doi: 10.2174/1568009621999210120193636. PubMed PMID: 33475064.

327. Yamamoto-Matsuda H, Miyoshi K, Moritoh M, Yoshitane H, Fukada Y, Saito K, et al. Lint-O cooperates with L(3)mbt in target gene suppression to maintain homeostasis in fly ovary and brain. EMBO Rep. 2022;23(10):e53813. Epub 20220822. doi: 10.15252/embr.202153813. PubMed PMID: 35993198; PubMed Central PMCID: PMCPMC9535798.

328. Yang L, Ge Y, Cheng D, Nie Z, Lv Z. Detection of piRNAs in whitespotted bamboo shark liver. Gene. 2016;590(1):51-6. Epub 20160604. doi: 10.1016/j.gene.2016.06.008. PubMed PMID: 27267405.

329. Yang X, Cheng Y, Lu Q, Wei J, Yang H, Gu M. Detection of stably expressed piRNAs in human blood. Int J Clin Exp Med. 2015;8(8):13353-8. Epub 20150815. PubMed PMID: 26550265; PubMed Central PMCID: PMCPMC4612950.

330. Yao J, Xie M, Ma X, Song J, Wang Y, Xue X. PIWI-interacting RNAs in cancer: Biogenesis, function, and clinical significance. Front Oncol. 2022;12:965684. Epub 20220923. doi: 10.3389/fonc.2022.965684. PubMed PMID: 36212439; PubMed Central PMCID: PMCPMC9539321.

331. Yao Y, Li Y, Zhu X, Zhao C, Yang L, Huang X, et al. The emerging role of the piRNA/PIWI complex in respiratory tract diseases. Respir Res. 2023;24(1):76. Epub 20230313. doi: 10.1186/s12931-023-02367-9. PubMed PMID: 36915129; PubMed Central PMCID: PMCPMC10010017.

332. Yin J, Qi W, Ji CG, Zhang DX, Xie XL, Ding Q, et al. Small RNA sequencing revealed aberrant piRNA expression profiles in colorectal cancer. Oncol Rep. 2019;42(1):263-72. Epub 20190513. doi: 10.3892/or.2019.7158. PubMed PMID: 31115555.

333. Yousefi B, Sadoughi F, Asemi Z, Mansournia MA, Hallajzadeh J. Novel Perspectives for the Diagnosis and Treatment of Gynecological Cancers using Dysregulation of PIWI Protein and PiRNAs as Biomarkers. Curr Med Chem. 2023. Epub 20230214. doi: 10.2174/0929867330666230214101837. PubMed PMID: 36786140.

334. Yu Y, Xiao J, Hann SS. The emerging roles of PIWI-interacting RNA in human cancers. Cancer Manag Res. 2019;11:5895-909. Epub 20190628. doi: 10.2147/cmar.S209300. PubMed PMID: 31303794; PubMed Central PMCID: PMCPMC6612017.

335. Zeuschner P, Linxweiler J, Junker K. Non-coding RNAs as biomarkers in liquid biopsies with a special emphasis on extracellular vesicles in urological malignancies. Expert Rev Mol Diagn. 2020;20(2):151-67. Epub 20190918. doi: 10.1080/14737159.2019.1665998. PubMed PMID: 31499007.

336. Zhang C. Novel functions for small RNA molecules. Curr Opin Mol Ther. 2009;11(6):641-51. PubMed PMID: 20072941; PubMed Central PMCID: PMCPMC3593927.

337. Zhang H, Ren Y, Xu H, Pang D, Duan C, Liu C. The expression of stem cell protein Piwil2 and piR-932 in breast cancer. Surg Oncol. 2013;22(4):217-23. Epub 20130827. doi: 10.1016/j.suronc.2013.07.001. PubMed PMID: 23992744.

338. Zhang L, Meng X, Li D, Han X. piR-001773 and piR-017184 promote prostate cancer progression by interacting with PCDH9. Cell Signal. 2020;76:109780. Epub 20200917. doi: 10.1016/j.cellsig.2020.109780. PubMed PMID: 32949716.

339. Zhang L, Wei P, Shen X, Zhang Y, Xu B, Zhou J, et al. MicroRNA Expression Profile in Penile Cancer Revealed by Next-Generation Small RNA Sequencing. PLoS One. 2015;10(7):e0131336. Epub 20150709. doi: 10.1371/journal.pone.0131336. PubMed PMID: 26158897; PubMed Central PMCID: PMCPMC4497725.

340. Zhang LM, Gao QX, Chen J, Li B, Li MM, Zheng L, et al. A universal catalytic hairpin assembly system for direct plasma biopsy of exosomal PIWI-interacting RNAs and microRNAs. Anal Chim Acta. 2022;1192:339382. Epub 20211218. doi: 10.1016/j.aca.2021.339382. PubMed PMID: 35057921.

341. Zhang T, Chen L, Li R, Liu N, Huang X, Wong G. PIWI-interacting RNAs in human diseases: databases and computational models. Brief Bioinform. 2022;23(4). doi: 10.1093/bib/bbac217. PubMed PMID: 35667080.

342. Zhang W, Liu H, Yin J, Wu W, Zhu D, Amos CI, et al. Genetic variants in the PIWI-piRNA pathway gene DCP1A predict melanoma disease-specific survival. Int J Cancer.

2016;139(12):2730-7. Epub 20160914. doi: 10.1002/ijc.30409. PubMed PMID: 27578485; PubMed Central PMCID: PMCPMC5282969.

343. Zhang W, Yao G, Wang J, Yang M, Wang J, Zhang H, et al. ncRPheno: a comprehensive database platform for identification and validation of disease related noncoding RNAs. RNA Biol. 2020;17(7):943-55. Epub 20200326. doi: 10.1080/15476286.2020.1737441. PubMed PMID: 32122231; PubMed Central PMCID: PMCPMC7549653.

344. Zhang Z, Zhang J, Diao L, Han L. Small non-coding RNAs in human cancer: function, clinical utility, and characterization. Oncogene. 2021;40(9):1570-7. Epub 20210115. doi: 10.1038/s41388-020-01630-3. PubMed PMID: 33452456.

345. Zhao C, Tolkach Y, Schmidt D, Toma M, Muders MH, Kristiansen G, et al. Mitochondrial PIWI-interacting RNAs are novel biomarkers for clear cell renal cell carcinoma. World J Urol. 2019;37(8):1639-47. Epub 20181128. doi: 10.1007/s00345-018-2575-1. PubMed PMID: 30488095.

346. Zhao L, Ning Q, Zheng G, Luo J, Dong D. exRNAdisease: An extracellular RNA transcriptome atlas in human diseases. Gene. 2022;836:146662. Epub 20220608. doi: 10.1016/j.gene.2022.146662. PubMed PMID: 35690280.

347. Zhao N, Deng Q, Zhu C, Zhang B. Mucus piRNAs profiles of Vibrio harveyi-infected Cynoglossus semilaevis: A hint for fish disease monitoring. J Fish Dis. 2022;45(1):165-75. Epub 20211106. doi: 10.1111/jfd.13546. PubMed PMID: 34741552.

348. Zhao PP, Yao MJ, Chang SY, Gou LT, Liu MF, Qiu ZL, et al. Novel function of PIWIL1 in neuronal polarization and migration via regulation of microtubule-associated proteins. Mol Brain. 2015;8:39. Epub 20150624. doi: 10.1186/s13041-015-0131-0. PubMed PMID: 26104391; PubMed Central PMCID: PMCPMC4477296.

349. Zheng K, Zhang XL, Wang L, You ZH, Ji BY, Liang X, et al. SPRDA: a link prediction approach based on the structural perturbation to infer disease-associated Piwi-interacting RNAs. Brief Bioinform. 2023;24(1). doi: 10.1093/bib/bbac498. PubMed PMID: 36445194.

350. Zhou J, Xie H, Liu J, Huang R, Xiang Y, Tian D, et al. PIWI-interacting RNAs: Critical roles and therapeutic targets in cancer. Cancer Lett. 2023;562:216189. Epub 20230417. doi: 10.1016/j.canlet.2023.216189. PubMed PMID: 37076042.

351. Zhou X, Liu J, Meng A, Zhang L, Wang M, Fan H, et al. Gastric juice piR-1245: A promising prognostic biomarker for gastric cancer. J Clin Lab Anal. 2020;34(4):e23131. Epub 20191128. doi: 10.1002/jcla.23131. PubMed PMID: 31777102; PubMed Central PMCID: PMCPMC7171314.

352. Zhu Y, Fan C, Zhao B. Differential expression of piRNAs in reprogrammed pluripotent stem cells from mouse embryonic fibroblasts. IUBMB Life. 2019;71(12):1906-15. Epub 20190718. doi: 10.1002/iub.2128. PubMed PMID: 31317647.

353. Zimta AA, Sigurjonsson OE, Gulei D, Tomuleasa C. The Malignant Role of Exosomes as Nanocarriers of Rare RNA Species. Int J Mol Sci. 2020;21(16). Epub 20200815. doi: 10.3390/ijms21165866. PubMed PMID: 32824183; PubMed Central PMCID: PMCPMC7461500.

354. Zoch A, Auchynnikava T, Berrens RV, Kabayama Y, Schöpp T, Heep M, et al. SPOCD1 is an essential executor of piRNA-directed de novo DNA methylation. Nature. 2020;584(7822):635-9. Epub 20200716. doi: 10.1038/s41586-020-2557-5. PubMed PMID: 32674113; PubMed

Central PMCID: PMCPMC7612247.

355. Zuo Y, Liang Y, Zhang J, Hao Y, Li M, Wen Z, et al. Transcriptome Analysis Identifies Piwi-Interacting RNAs as Prognostic Markers for Recurrence of Prostate Cancer. Front Genet. 2019;10:1018. Epub 20191022. doi: 10.3389/fgene.2019.01018. PubMed PMID: 31695724; PubMed Central PMCID: PMCPMC6817565.

356. Zuo Z, Hu H, Xu Q, Luo X, Peng D, Zhu K, et al. BBCancer: an expression atlas of blood-based biomarkers in the early diagnosis of cancers. Nucleic Acids Res. 2020;48(D1):D789-d96. doi: 10.1093/nar/gkz942. PubMed PMID: 31665503; PubMed Central PMCID: PMCPMC7145713.

357. Maguire S, Lohman GJS, Guan S. A low-bias and sensitive small RNA library preparation method using randomized splint ligation. Nucleic acids research. 2020;48(14):e80. doi: 10.1093/nar/gkaa480. PubMed PMID: CN-02212803.

358. Abstracts of the 4th International Conference on Advances in Hematology and Oncology, ICAHO 2016. Anticancer Research. 2016;36(12).

359. Andrology centres accredited by the European Academy of Andrology. Andrology. 2022;10(S2):10-96. doi: 10.1111/andr.13245.

360. Ahamad N, Gupta S, Parashar D. Using Omics to Study Leprosy, Tuberculosis, and Other Mycobacterial Diseases. Frontiers in Cellular and Infection Microbiology. 2022;12. doi: 10.3389/fcimb.2022.792617.

361. Allegra A, Cicero N, Tonacci A, Musolino C, Gangemi S. Circular RNA as a Novel Biomarker for Diagnosis and Prognosis and Potential Therapeutic Targets in Multiple Myeloma. Cancers. 2022;14(7). doi: 10.3390/cancers14071700.

362. Allegra A, Di Gioacchino M, Tonacci A, Petrarca C, Musolino C, Gangemi S. Multiple myeloma cell-derived exosomes: Implications on tumorigenesis, diagnosis, prognosis and therapeutic strategies. Cells. 2021;10(11). doi: 10.3390/cells10112865.

363. Amaar YG, Reeves ME. The impact of the RASSF1C-PIWIL1-piRNA pathway on DNA methylation. Cancer Research. 2018;78(13). doi: 10.1158/1538-7445.AM2018-365.

364. Amaar YG, Reeves ME. RASSF1C and tumor microenvironment. Cancer Research. 2022;82(12). doi: 10.1158/1538-7445.AM2022-3839.

365. Ameli Mojarad M, Ameli Mojarad M, Shojaee B, Nazemalhosseini-Mojarad E. piRNA: A promising biomarker in early detection of gastrointestinal cancer. Pathology Research and Practice. 2022;230. doi: 10.1016/j.prp.2021.153757.

366. Ameli-Mojarad M, Ameli-Mojarad M, Hadizadeh M, Young C, Babini H, Nazemalhosseini-Mojarad E, et al. The effective function of circular RNA in colorectal cancer. Cancer Cell International. 2021;21(1). doi: 10.1186/s12935-021-02196-0.

367. AmeliMojarad M, Amelimojarad M. piRNAs and PIWI proteins as potential biomarkers in Breast cancer. Molecular Biology Reports. 2022;49(10):9855-62. doi: 10.1007/s11033-022-07506-x.

368. AmeliMojarad M, AmeliMojarad M, Wang J. The function of novel small non-coding RNAs (piRNAs, tRFs) and PIWI protein in colorectal cancer. Cancer Treatment and Research Communications. 2022;31. doi: 10.1016/j.ctarc.2022.100542.

369. Assumpção CB, Calcagno DQ, Araújo TMT, Batista Dos Santos SE, Ribeiro Dos Santos ÂKC, Riggins GJ, et al. The role of piRNA and its potential clinical implications in cancer. Epigenomics. 2015;7(6):975-84. doi: 10.2217/epi.15.37.

370. Bahn JH, Zhang Q, Li F, Chan TM, Lin X, Kim Y, et al. The landscape of MicroRNA, piwi-interacting RNA, and circular RNA in human saliva. Clinical Chemistry. 2015;61(1):221-30. doi: 10.1373/clinchem.2014.230433.

371. Bajpai J, Kümar P, Arora B, Kirkire P, Nair R, Medekar A, et al. Comparison of isolates and antibiotic sensitivity pattern in paediatric and adult cancer patients; Is it different? Pediatric Blood and Cancer. 2010;55(5):955. doi: 10.1002/pbc.22779.

372. Balaratnam S, West N, Basu S. A piRNA utilizes HILI and HIWI2 mediated pathway to down-regulate ferritin heavy chain 1 mRNA in human somatic cells. Nucleic Acids Research. 2018;46(20):10635-48. doi: 10.1093/nar/gky728.

373. Balatti V, Pekarsky Y, Croce CM. Role of the tRNA-Derived Small RNAs in Cancer: New Potential Biomarkers and Target for Therapy. 2017. p. 173-87.

374. Bartos M, Siegl F, Kopkova A, Radova L, Oppelt J, Vecera M, et al. Small RNA Sequencing Identifies PIWI-Interacting RNAs Deregulated in Glioblastoma—piR-9491 and piR-12488 Reduce Tumor Cell Colonies In Vitro. Frontiers in Oncology. 2021;11. doi: 10.3389/fonc.2021.707017.

375. Beg A, Parveen R, Fouad H, Yahia ME, Hassanein AS. Role of different non-coding RNAs as ovarian cancer biomarkers. Journal of Ovarian Research. 2022;15(1). doi: 10.1186/s13048-022-01002-3.

376. Bhatti GK, Khullar N, Sidhu IS, Navik US, Reddy AP, Reddy PH, et al. Emerging role of non-coding RNA in health and disease. Metabolic Brain Disease. 2021;36(6):1119-34. doi: 10.1007/s11011-021-00739-y.

377. Bian H, Zhou Y, Zhou D, Zhang Y, Shang D, Qi J. The latest progress on miR-374 and its functional implications in physiological and pathological processes. Journal of Cellular and Molecular Medicine. 2019;23(5):3063-76. doi: 10.1111/jcmm.14219.

378. Bijnsdorp IV, van Royen ME, Verhaegh GW, Martens-Uzunova ES. The Non-Coding Transcriptome of Prostate Cancer: Implications for Clinical Practice. Molecular Diagnosis and Therapy. 2017;21(4):385-400. doi: 10.1007/s40291-017-0271-2.

379. Bolton EM, Tuzova AV, Walsh AL, Lynch T, Perry AS. Noncoding RNAs in prostate cancer: The long and the short of it CME. Clinical Cancer Research. 2014;20(1):35-43. doi: 10.1158/1078-0432.CCR-13-1989.

380. Bomken N, Mysina S, Bomken C, Heidenreich O, Vormoor H. The germline stem cell self-renewal gene PIWIL2 is expressed in both malignant and non-malignant lymphoid cells. Haematologica. 2010;95:6.

381. Bonifer C. Why detailed model gene studies in higher eukaryotes are still necessary. Immunology. 2013;139(2):158-60. doi: 10.1111/imm.12066.

382. Borga C, Meeran SM, Fassan M. Non-coding RNAs, a real Next-Gen Class of Biomarkers? Non-coding RNA Research. 2019;4(3):80-1. doi: 10.1016/j.ncrna.2019.10.001.

383. Bornelöv S, Czech B, Hannon GJ. An evolutionarily conserved stop codon enrichment at the 5' ends of mammalian piRNAs. 2021.

384. Bornelöv S, Czech B, Hannon GJ. An evolutionarily conserved stop codon enrichment at the 5′ ends of mammalian piRNAs. Nature Communications. 2022;13(1). doi: 10.1038/s41467-022-29787-3.

385. Burton J, Hammer H, Umu S, Langseth H, Grotmol T, Grimsrud T, et al. Small non-coding RNA in serum from testicular germ cell tumour patientsidentified by machine learning. ESMO Open. 2018;3:A255-A6. doi: 10.1136/esmoopen-2018-EACR25.603.

386. Cammarata G, de Miguel-Perez D, Russo A, Peleg A, Dolo V, Rolfo C, et al. Emerging noncoding RNAs contained in extracellular vesicles: rising stars as biomarkers in lung cancer liquid biopsy. Therapeutic Advances in Medical Oncology. 2022;14. doi: 10.1177/17588359221131229.

387. Cao F, Li X, Hiew S, Brady H, Liu Y, Dou Y. Dicer independent small RNAs associate with telomeric heterochromatin. RNA. 2009;15(7):1274-81. doi: 10.1261/rna.1423309.

388. Cao J, Xu G, Lan J, Huang Q, Tang Z, Tian L. High expression of piwi-like RNA-mediated gene silencing 1 is associated with poor prognosis via regulating transforming growth factor-β receptors and cyclin-dependent kinases in breast cancer. Molecular Medicine Reports. 2016;13(3):2829-35. doi: 10.3892/mmr.2016.4842.

389. Cao P, Jin Q, Feng L, Li H, Qin G, Zhou G. Emerging roles and potential clinical applications of noncoding RNAs in hepatocellular carcinoma. Seminars in Cancer Biology. 2021;75:136-52. doi: 10.1016/j.semcancer.2020.09.003.

390. Cass AA, Bahn JH, Lee JH, Greer C, Lin X, Kim Y, et al. Global analyses of endonucleolytic cleavage in mammals reveal expanded repertoires of cleavage-inducing small RNAs and their targets. Nucleic Acids Research. 2016;44(7):3253-63. doi: 10.1093/nar/gkw164.

391. Chalbatani GM, Dana H, Memari F, Gharagozlou E, Ashjaei S, Kheirandish P, et al. Biological function and molecular mechanism of piRNA in cancer. Practical Laboratory Medicine. 2019;13. doi: 10.1016/j.plabm.2018.e00113.

392. Chang Z, Ji G, Huang R, Chen H, Gao Y, Wang W, et al. PIWI-interacting RNAs piR-13643 and piR-21238 are promising diagnostic biomarkers of papillary thyroid carcinoma. Aging. 2020;12(10):9292-310. doi: 10.18632/aging.103206.

393. Chavda V, Madhwani K, Chaurasia B. PiWi RNA in Neurodevelopment and Neurodegenerative Disorders. Current Molecular Pharmacology. 2022;15(3):517-31. doi: 10.2174/1874467214666210629164535.

394. Chen H, Zhuang Z, Chen Y, Qiu C, Qin Y, Tan C, et al. A universal platform for one-pot detection of circulating non-coding RNA combining CRISPR-Cas12a and branched rolling circle amplification. Analytica Chimica Acta. 2023;1246. doi: 10.1016/j.aca.2023.340896.

395. Chen L, Chen Z, Simões A, Wu X, Dai Y, Dipietro LA, et al. Site-specific expression pattern of PIWI-interacting RNA in skin and oral mucosal wound healing. International Journal of Molecular Sciences. 2020;21(2). doi: 10.3390/ijms21020521.

396. Chen S, Ben S, Xin J, Li S, Zheng R, Wang H, et al. The biogenesis and biological function of PIWI-interacting RNA in cancer. Journal of Hematology and Oncology. 2021;14(1). doi: 10.1186/s13045-021-01104-3.

397. Chen S, Deng Y. PiRADL: An advanced deep learning tool for piRNA detection and its application in cancer biomarker identification. Cancer Research. 2020;80(16 SUPPL). doi:

10.1158/1538-7445.AM2020-LB-249.

398. Chen X, Guo DY, Yin TL, Yang J. Non-Coding RNAs Regulate Placental Trophoblast Function and Participate in Recurrent Abortion. Frontiers in Pharmacology. 2021;12. doi: 10.3389/fphar.2021.646521.

399. Chen YCA, Stuwe E, Luo Y, Ninova M, Le Thomas A, Rozhavskaya E, et al. Cutoff Suppresses RNA Polymerase II Termination to Ensure Expression of piRNA Precursors. Molecular Cell. 2016;63(1):97-109. doi: 10.1016/j.molcel.2016.05.010.

400. Cheng J, Guo JM, Xiao BX, Miao Y, Jiang Z, Zhou H, et al. PiRNA, the new non-coding RNA, is aberrantly expressed in human cancer cells. Clinica Chimica Acta. 2011;412(17-18):1621-5. doi: 10.1016/j.cca.2011.05.015.

401. Chi T, Lin J, Wang M, Zhao Y, Liao Z, Wei P. Non-Coding RNA as Biomarkers for Type 2 Diabetes Development and Clinical Management. Frontiers in Endocrinology. 2021;12. doi: 10.3389/fendo.2021.630032.

402. Cho O, Kim DW, Cheong JY. Screening plasma exosomal RNAs as diagnostic markers for cervical cancer: An analysis of patients who underwent primary chemoradiotherapy. Biomolecules. 2021;11(11). doi: 10.3390/biom11111691.

403. Chu YL, Li H, Ng PLA, Kong ST, Zhang H, Lin Y, et al. The potential of circulating exosomal RNA biomarkers in cancer. Expert Review of Molecular Diagnostics. 2020;20(7):665-78. doi: 10.1080/14737159.2020.1745064.

404. Conrad K, Röber N, Achtleitner M, Aringer M, Rudolph S, Unger L, et al. Anti-DFS70 antibody- a biomarker that aid in the exclusion of ANA associated rheumatic diseases. Annals of the Rheumatic Diseases. 2017;76:461. doi: 10.1136/annrheumdis-2017-eular.5915.

405. Cui L, Lou Y, Zhang X, Zhou H, Deng H, Song H, et al. Detection of circulating tumor cells in peripheral blood from patients with gastric cancer using piRNAs as markers. Clinical Biochemistry. 2011;44(13):1050-7. doi: 10.1016/j.clinbiochem.2011.06.004.

406. Dabi Y, Favier A, Razakamanantsoa L, Delbos L, Poilblanc M, Descamps P, et al. Insight on Non-Coding RNAs from Biofluids in Ovarian Tumors. Cancers. 2023;15(5). doi: 10.3390/cancers15051539.

407. Das B, Jain N, Mallick B. piR-39980 mediates doxorubicin resistance in fibrosarcoma by regulating drug accumulation and DNA repair. Communications biology. 2021;4(1):1312. doi: 10.1038/s42003-021-02844-1.

408. Das T, Deb A, Parida S, Mondal S, Khatua S, Ghosh Z. LncRBase V.2: an updated resource for multispecies lncRNAs and ClinicLSNP hosting genetic variants in lncRNAs for cancer patients. RNA Biology. 2021;18(8):1136-51. doi: 10.1080/15476286.2020.1833529.

409. Dates CR, Tollefsbol TO. Transforming cancer epigenetics using nutritive approaches and noncoding RNAs. Current Cancer Drug Targets. 2018;18(1):32-8. doi: 10.2174/1568009617666170203165326.

410. Daugaard I, Venø MT, Yan Y, Kjeldsen TE, Lamy P, Hager H, et al. Small RNA sequencing reveals metastasis-related microRNAs in lung adenocarcinoma. Oncotarget. 2017;8(16):27047-61. doi: 10.18632/oncotarget.15968.

411. Davis R, Wongworawat YC, Perez MC, Santiago K, Roy S, Wang C, et al. Upregulated and downregulatedmicroRNA-signatures in thyroid cancer health disparities. Cancer

Epidemiology Biomarkers and Prevention. 2020;29(6 SUPPL 2). doi: 10.1158/1538-7755.DISP19-B093.

412. Diamantopoulos MA, Tsiakanikas P, Scorilas A. Non-coding RNAs: The riddle of the transcriptome and their perspectives in cancer. Annals of Translational Medicine. 2018;6(12). doi: 10.21037/atm.2018.06.10.

413. Dietrich D, Meller S, Uhl B, Ralla B, Stephan C, Jung K, et al. Nucleic acid-based tissue biomarkers of urologic malignancies. Critical Reviews in Clinical Laboratory Sciences. 2014;51(4):173-99. doi: 10.3109/10408363.2014.906130.

414. Ding L, Jiang M, Wang R, Shen D, Wang H, Lu Z, et al. The emerging role of small non-coding RNA in renal cell carcinoma. Translational Oncology. 2021;14(1). doi: 10.1016/j.tranon.2020.100974.

415. Dong P, Xiong Y, Konno Y, Ihira K, Xu D, Kobayashi N, et al. Critical Roles of PIWIL1 in Human Tumors: Expression, Functions, Mechanisms, and Potential Clinical Implications. Frontiers in Cell and Developmental Biology. 2021;9. doi: 10.3389/fcell.2021.656993.

416. Dostalova Merkerova M, Hrustincova A, Krejcik Z, Kundrat D, Szikszai K, Cermak J, et al. Circulating Small Noncoding RNAs As Novel Semi-Invasive Markers of Patient Survival in Myelodysplastic Syndromes. Blood. 2019;134:3004. doi: 10.1182/blood-2019-126520.

417. Dvorská D, Braný D, Ňachajová M, Halašová E, Danková Z. Breast cancer and the other non-coding RNAs. International Journal of Molecular Sciences. 2021;22(6). doi: 10.3390/ijms22063280.

418. Edwards SL, Beesley J, French JD, Dunning M. Beyond GWASs: Illuminating the dark road from association to function. American Journal of Human Genetics. 2013;93(5):779-97. doi: 10.1016/j.ajhg.2013.10.012.

419. Ellinger J, Gevensleben H, Müller SC, Dietrich D. The emerging role of non-coding circulating RNA as a biomarker in renal cell carcinoma. Expert Review of Molecular Diagnostics. 2016;16(10):1059-65. doi: 10.1080/14737159.2016.1239531.

420. Eslava-Avilés E, Arenas-Huertero F. Pirnas: Nature, biogenesis, regulation, and their potential clinical utility. Boletin Medico del Hospital Infantil de Mexico. 2021;78(5):432-42. doi: 10.24875/BMHIM.20000185.

421. Fadrus P, Vybihal V, Siegl F, Smrcka M, Slaby O, Sana J. STUDY OF PIWI-INTERACTING RNAS IN GLIOBLASTOMA PATHOLOGY: A NEW LEVEL OF REGULATION OF GLIOBLASTOMA STEM CELLS? Neuro-Oncology. 2022;24:vii38. doi: 10.1093/neuonc/noac209.148.

422. Fan L, Zhong Z, Lin Y, Li J. Non-coding RNAs as potential biomarkers in osteosarcoma. Frontiers in Genetics. 2022;13. doi: 10.3389/fgene.2022.1028477.

423. Feng J, Yang M, Wei Q, Song F, Zhang Y, Wang X, et al. Novel evidence for oncogenic piRNA-823 as a promising prognostic biomarker and a potential therapeutic target in colorectal cancer. Journal of Cellular and Molecular Medicine. 2020;24(16):9028-40. doi: 10.1111/jcmm.15537.

424. Ferguson LB, Mayfield RD, Messing RO. RNA biomarkers for alcohol use disorder. Frontiers in Molecular Neuroscience. 2022;15. doi: 10.3389/fnmol.2022.1032362.

425. Ferrari A, Neefs I, Hoeck S, Peeters M, Van Hal G. Towards novel non-invasive colorectal

cancer screening methods: A comprehensive review. Cancers. 2021;13(8). doi: 10.3390/cancers13081820.

426. Fitz NF, Wang J, Kamboh MI, Koldamova R, Lefterov I. Small nucleolar RNAs in plasma extracellular vesicles and their discriminatory power as diagnostic biomarkers of Alzheimer's disease. Neurobiology of Disease. 2021;159. doi: 10.1016/j.nbd.2021.105481.

427. Flores-Bellver M, Zhou J, Zhong X, Benito-Martín A, Mighty J, Qian J, et al. Extracellular vesicles released from human iPSC-derived 3D retinas contain small RNAs with roles in development and differentiation. Journal of Extracellular Vesicles. 2019;8:90. doi: 10.1080/20013078.2019.1593587.

428. Garcia LC, Peterson T, Parney I. miRNA signature derived from GBM plasma exosomes as a diagnostic biomarker. Neuro-Oncology. 2018;20:vi141. doi: 10.1093/neuonc/noy148.

429. Garcia LMC, Chanana P, Parney I. MiRNA signature derived from GBM plasma exosomes as a diagnostic biomarker. Journal of Extracellular Vesicles. 2019;8:131. doi: 10.1080/20013078.2019.1593587.

430. Ge L, Zhang N, Li D, Wu Y, Wang H, Wang J. Circulating exosomal small RNAs are promising non-invasive diagnostic biomarkers for gastric cancer. Journal of Cellular and Molecular Medicine. 2020;24(24):14502-13. doi: 10.1111/jcmm.16077.

431. Ge X, Tang L, Wang Y, Wang N, Zhou J, Deng X, et al. The diagnostic value of exosomal miRNAs in human bile of malignant biliary obstructions. Digestive and Liver Disease. 2021;53(6):760-5. doi: 10.1016/j.dld.2020.11.010.

432. Georgia S, Arda HE, Martinez-Sanchez A, Dhawan S. Editorial: Epigenetics of Glucose Homeostasis. Frontiers in Endocrinology. 2022;13. doi: 10.3389/fendo.2022.889189.

433. Ghaseminezhad Z, Sharifi M, Bahreini A, Mehrzad V. Investigation of the expression of P-element-induced wimpy testis-interacting RNAs in human acute myeloid leukemia. Meta Gene. 2022;31. doi: 10.1016/j.mgene.2021.100998.

434. Ghazimoradi MH, Karimpour-Fard N, Babashah S. The Promising Role of Non-Coding RNAs as Biomarkers and Therapeutic Targets for Leukemia. Genes. 2023;14(1). doi: 10.3390/genes14010131.

435. Giurato G, De Filippo MR, Rinaldi A, Hashim A, Nassa G, Ravo M, et al. iMir: an integrated pipeline for high-throughput analysis of small non-coding RNA data obtained by smallRNA-Seq. BMC bioinformatics. 2013;14:362. doi: 10.1186/1471-2105-14-362.

436. Goukassian D, Truongcao M, Arakelyan A, Mills P, Walsh K, Addya S, et al. Exosomal rnas as potential biomarkers of cardiovascular risk in nasa astronauts. Circulation. 2019;140. doi: 10.1161/circ.140.suppl_1.12916.

437. Goyal R, Wassie M, Winter J, Lathlean T, Young G, Symonds E. Accuracy of blood-based biomarkers for detection of advanced colorectal adenomas: A systematic review. Journal of Gastroenterology and Hepatology. 2022;37:30-1. doi: 10.1111/jgh.15949.

438. Gu X, Zhang Y, Qin X, Ma S, Huang Y, Ju S. Transfer RNA-derived small RNA: an emerging small non-coding RNA with key roles in cancer. Experimental Hematology and Oncology. 2022;11(1). doi: 10.1186/s40164-022-00290-1.

439. Halajzadeh J, Dana PM, Asemi Z, Mansournia MA, Yousefi B. An insight into the roles of piRNAs and PIWI proteins in the diagnosis and pathogenesis of oral, esophageal, and gastric

cancer. Pathology Research and Practice. 2020;216(10). doi: 10.1016/j.prp.2020.153112.

440. Hallal S, Khani SE, Wei H, Lee MYT, Sim HW, Sy J, et al. Deep sequencing of small RNAs from neurosurgical extracellular vesicles substantiates miR-486-3p as a circulating biomarker that distinguishes glioblastoma from lower-grade astrocytoma patients. International Journal of Molecular Sciences. 2020;21(14):1-22. doi: 10.3390/ijms21144954.

441. Han YN, Li Y, Xia SQ, Zhang YY, Zheng JH, Li W. PIWI Proteins and PIWI-Interacting RNA: Emerging Roles in Cancer. Cellular Physiology and Biochemistry. 2017;44(1):1-20. doi: 10.1159/000484541.

442. Hanusek K, Poletajew S, Kryst P, Piekiełko-Witkowska A, Bogusławska J. piRNAs and PIWI Proteins as Diagnostic and Prognostic Markers of Genitourinary Cancers. Biomolecules. 2022;12(2). doi: 10.3390/biom12020186.

443. Hartikainen JM, Heikkinen S, Tengström M, Kosma VM, Mannermaa A. Subtype-specific expression of small non-coding RNAs in breast cancer. Cancer Research. 2017;77(13). doi: 10.1158/1538-7445.AM2017-3492.

444. Hauptman N, Glavac D. MicroRNAs and long non-coding RNAs: Prospects in diagnostics and therapy of cancer. Radiology and Oncology. 2013;47(4):311-8. doi: 10.2478/raon-2013-0062.

445. Hayashi K, Chuva de Sousa Lopes SM, Kaneda M, Tang F, Hajkova P, Lao K, et al. MicroRNA biogenesis is required for mouse primordial germ cell development and spermatogenesis. PLoS ONE. 2008;3(3). doi: 10.1371/journal.pone.0001738.

446. Hirschfeld M, Ritter A, Berner K, Rucker G, Jager M, Weis D, et al. Circulating non-coding RNA-biomarker potential in neoadjuvant chemotherapy of triple negative breast cancer? Oncology Research and Treatment. 2020;43:15. doi: 10.1159/000506491.

447. Hong Y, Wang C, Fu Z, Liang H, Zhang S, Lu M, et al. Systematic characterization of seminal plasma piRNAs as molecular biomarkers for male infertility. Scientific reports. 2016;6:24229. doi: 10.1038/srep24229.

448. Hosseinalizadeh H, Mahmoodpour M, Ebrahimi A. Circulating non-coding RNAs as a diagnostic and management biomarker for breast cancer: current insights. Molecular Biology Reports. 2022;49(1):705-15. doi: 10.1007/s11033-021-06847-3.

449. Hua J, Lu J, Isaev K, Soares F, Guo H, Ahmed M, et al. Noncoding RNA for personalized prostate cancer treatment: Utilizing the 'dark matters' of the genome. Personalized Medicine. 2017;14(2):159-69. doi: 10.2217/pme-2016-0090.

450. Huang G, Hu H, Xue X, Shen S, Gao E, Guo G, et al. Altered expression of piRNAs and their relation with clinicopathologic features of breast cancer. Clinical and Translational Oncology. 2013;15(7):563-8. doi: 10.1007/s12094-012-0966-0.

451. Huang HY, Houwing S, Kaaij LJT, Meppelink A, Redl S, Gauci S, et al. Tdrd1 acts as a molecular scaffold for Piwi proteins and piRNA targets in zebrafish. EMBO Journal. 2011;30(16):3298-308. doi: 10.1038/emboj.2011.228.

452. Huang X, Yuan T, Tschannen M, Sun Z, Jacob H, Du M, et al. Characterization of human plasma-derived exosomal RNAs by deep sequencing. BMC Genomics. 2013;14(1). doi: 10.1186/1471-2164-14-319.

453. Huang Y, Ji L, Huang Q, Vassylyev DG, Chen X, Ma JB. Structural insights into mechanisms of the small RNA methyltransferase HEN1. Nature. 2009;461(7265):823-7. doi:

10.1038/nature08433.

454. Iliev R, Vychytilova-Faltejskova P, Juracek J, Mlcochova H, Stanik M, Dolezel J, et al. Piwi genes and tissue/serum piR-651 are related to clinicopathologic features of renal cell carcinoma. Cancer Research. 2015;75(15). doi: 10.1158/1538-7445.AM2015-237.

455. Iyer DN, Wan TMH, Man JHW, Sin RWY, Li X, Lo OSH, et al. Small RNA profiling of piRNAs in colorectal cancer identifies consistent overexpression of piR-24000 that correlates clinically with an aggressive disease phenotype. Cancers. 2020;12(1). doi: 10.3390/cancers12010188.

456. Jacobs DI, Qin Q, Fu A, Chen Z, Zhou J, Zhu Y. A translational investigation of piRNAs in glioblastoma multiforme. Cancer Research. 2016;76(14). doi: 10.1158/1538-7445.AM2016-960.

457. Jaeger H, Larsen K, Thorsen K, Umu S, Rounge T, Bache K. Circulating non-coding rna as early diagnostic biomarkers in acute stroke-a pilot study. International Journal of Stroke. 2020;15(1 SUPPL):166. doi: 10.1177/1747493020963387.

458. Janšáková K, Kyselicová K, Ostatníková D, Repiská G. Potential of salivary biomarkers in autism research: A systematic review. International Journal of Molecular Sciences. 2021;22(19). doi: 10.3390/ijms221910873.

459. Jia J, Yang S, Huang J, Zheng H, He Y, Wang L. Distinct Extracellular RNA Profiles in Different Plasma Components. Frontiers in Genetics. 2021;12. doi: 10.3389/fgene.2021.564780.

460. Jia R, He X, Ma W, Lei Y, Cheng H, Sun H, et al. Aptamer-Functionalized Activatable DNA Tetrahedron Nanoprobe for PIWI-Interacting RNA Imaging and Regulating in Cancer Cells. Analytical chemistry. 2019;91(23):15107-13. doi: 10.1021/acs.analchem.9b03819.

461. Jima DD, Zhang J, Jacobs C, Richards KL, Dunphy CH, Choi WWL, et al. Deep sequencing of the small RNA transcriptome of normal and malignant human B cells identifies hundreds of novel microRNAs. Blood. 2010;116(23):e118-e27. doi: 10.1182/blood-2010-05-285403.

462. Kamminga LM, van Wolfswinkel JC, Luteijn MJ, Kaaij LJT, Bagijn MP, Sapetschnig A, et al. Differential impact of the HEN1 homolog HENN-1 on 21U and 26G RNAs in the germline of Caenorhabditis elegans. PLoS Genetics. 2012;8(7). doi: 10.1371/journal.pgen.1002702.

463. Kan CFK, Unis GD, Li LZ, Gunn S, Li L, Soyer HP, et al. Circulating Biomarkers for Early Stage Non-Small Cell Lung Carcinoma Detection: Supplementation to Low-Dose Computed Tomography. Frontiers in Oncology. 2021;11. doi: 10.3389/fonc.2021.555331.

464. Karimpour M, Ravanbakhsh R, Maydanchi M, Rajabi A, Azizi F, Saber A. Cancer driver gene and non-coding RNA alterations as biomarkers of brain metastasis in lung cancer: A review of the literature. Biomedicine and Pharmacotherapy. 2021;143. doi: 10.1016/j.biopha.2021.112190.

465. Kazimierczyk M, Jędroszkowiak A, Kowalczykiewicz D, Szymański M, Imiołczyk B, Ciesiołka J, et al. tRNA-derived fragments from the Sus scrofa tissues provide evidence of their conserved role in mammalian development. Biochemical and Biophysical Research Communications. 2019;520(3):514-9. doi: 10.1016/j.bbrc.2019.10.062.

466. Kellis M, Wold B, Snyder MP, Bernstein BE, Kundaje A, Marinov GK, et al. Defining functional DNA elements in the human genome. Proceedings of the National Academy of Sciences of the United States of America. 2014;111(17):6131-8. doi: 10.1073/pnas.1318948111.

467. Kim DH, Rossi JJ. Overview of gene silencing by RNA interference. Current Protocols in

Nucleic Acid Chemistry. 2009;(SUPPL. 36):16.1.1-.1-0. doi: 10.1002/0471142700.nc1601s36.

468. Kishikawa T, Otsuka M, Ohno M, Yoshikawa T, Takata A, Koike K. Circulating RNAs as new biomarkers for detecting pancreatic cancer. World Journal of Gastroenterology. 2015;21(28):8527-40. doi: 10.3748/wjg.v21.i28.8527.

469. Kishore C, Karunagaran D. Non-coding RNAs as emerging regulators and biomarkers in colorectal cancer. Molecular and Cellular Biochemistry. 2022;477(6):1817-28. doi: 10.1007/s11010-022-04412-5.

470. Kocic G, Hadzi-Djokic J, Colic M, Veljkovic A, Tomovic K, Roumeliotis S, et al. The Role of Nucleases Cleaving TLR3, TLR7/8 and TLR9 Ligands, Dicer RNase and miRNA/piRNA Proteins in Functional Adaptation to the Immune Escape and Xenophagy of Prostate Cancer Tissue. International Journal of Molecular Sciences. 2023;24(1). doi: 10.3390/ijms24010509.

471. Koduru SV, Leberfinger AN, Ravnic DJ. Small non-coding RNA abundance in adrenocortical carcinoma: A footprint of a rare cancer. Journal of Genomics. 2017;5:99-114. doi: 10.7150/jgen.22060.

472. Koduru SV, Nyinawabera A, Ravnic DJ, Tiwari AK. Interrogation of small RNA-seq data for small noncoding RNA in human colon cancer. Cancer Research. 2017;77(13). doi: 10.1158/1538-7445.AM2017-3490.

473. Koduru SV, Ravnic DJ. Noncoding RNA distribution in clear cell renal cell cancer: Small RNAseq data. Cancer Research. 2017;77(13). doi: 10.1158/1538-7445.AM2017-3489.

474. Koduru SV, Tiwari AK, Hazard SW, Mahajan MK, Ravnic DJ. Analysis of small RNA-seq data for differential expression of small noncoding RNAs in human colorectal cancer. Cancer Research. 2017;77(13). doi: 10.1158/1538-7445.AM2017-4447.

475. Koduru SV, Tiwari AK, Leberfinger A, Hazard SW, Kawasawa YI, Mahajan M, et al. Differentially expressed small noncoding RNAs in triple-negative breast cancer. Cancer Research. 2017;77(13). doi: 10.1158/1538-7445.AM2017-3499.

476. Koduru SV, Tiwari AK, Leberfinger A, Hazard SW, Kawasawa YI, Mahajan M, et al. A comprehensive NGS data analysis of differentially regulated miRNAs, piRNAs, lncRNas and sn/snoRNAs in triple negative breast cancer. Journal of Cancer. 2017;8(4):578-96. doi: 10.7150/jca.17633.

477. Kolenda T, Guglas K, Baranowski D, Sobocińska J, Kopczyńska M, Teresiak A, et al. cfRNAs as biomarkers in oncology – still experimental or applied tool for personalized medicine already? Reports of Practical Oncology and Radiotherapy. 2020;25(5):783-92. doi: 10.1016/j.rpor.2020.07.007.

478. Krishnan P, Damaraju S. The Challenges and Opportunities in the Clinical Application of Noncoding RNAs: The Road Map for miRNAs and piRNAs in Cancer Diagnostics and Prognostics. International Journal of Genomics. 2018;2018. doi: 10.1155/2018/5848046.

479. Ku J, Zou AE, Honda TK, Zheng H, Saad MA, Yu V, et al. Identification of key survival-correlating microRNAs and Piwi-interacting RNAs dysregulated in head and neck squamous cell carcinoma. Cancer Research. 2015;75(15). doi: 10.1158/1538-7445.AM2015-3836.

480. Kubiliute R, Jarmalaite S. Epigenetic biomarkers of renal cell carcinoma for liquid biopsy tests. International Journal of Molecular Sciences. 2021;22(16). doi: 10.3390/ijms22168846.

481. Kugelberg U, Nätt D, Skog S, Kutter C, Öst A. 5´XP sRNA-seq: efficient identification of

transcripts with and without 5´ phosphorylation reveals evolutionary conserved small RNA. RNA Biology. 2021;18(11):1588-99. doi: 10.1080/15476286.2020.1861770.

482. Kumar SR, Kimchi ET, Manjunath Y, Gajagowni S, Stuckel AJ, Kaifi JT. RNA cargos in extracellular vesicles derived from blood serum in pancreas associated conditions. Scientific reports. 2020;10(1):2800. doi: 10.1038/s41598-020-59523-0.

483. Kunnummal M, Angelin M, Das AV. PIWI proteins and piRNAs in cervical cancer: a propitious dart in cancer stem cell-targeted therapy. Human Cell. 2021;34(6):1629-41. doi: 10.1007/s13577-021-00590-4.

484. Kuo MC, Liu SCH, Hsu YF, Wu RM. The role of noncoding RNAs in Parkinson's disease: biomarkers and associations with pathogenic pathways. Journal of Biomedical Science. 2021;28(1). doi: 10.1186/s12929-021-00775-x.

485. Law PTY, Qin H, Ching AKK, Lai KP, Co NN, He M, et al. Deep sequencing of small RNA transcriptome reveals novel non-coding RNAs in hepatocellular carcinoma. Journal of Hepatology. 2013;58(6):1165-73. doi: 10.1016/j.jhep.2013.01.032. PubMed Central PMCID: PMCExiqon(Denmark).

486. Le P, Romano G, Nana-Sinkam P, Acunzo M. Non-coding rnas in cancer diagnosis and therapy: Focus on lung cancer. Cancers. 2021;13(6):1-25. doi: 10.3390/cancers13061372.

487. Lee KY, Seo Y, Im JH, Rhim J, Baek W, Kim S, et al. Molecular signature of extracellular vesicular small non-coding rnas derived from cerebrospinal fluid of leptomeningeal metastasis patients: Functional implication of mir-21 and other small rnas in cancer malignancy. Cancers. 2021;13(2):1-22. doi: 10.3390/cancers13020209.

488. Lewis A, Berkyurek AC, Greiner A, Sawh AN, Vashisht A, Merrett S, et al. A Family of Argonaute-Interacting Proteins Gates Nuclear RNAi. Molecular Cell. 2020;78(5):862-75.e8. doi: 10.1016/j.molcel.2020.04.007.

489. Li C, Qin F, Hu F, Xu H, Sun G, Han G, et al. Characterization and selective incorporation of small non-coding RNAs in non-small cell lung cancer extracellular vesicles. Cell and Bioscience. 2018;8(1). doi: 10.1186/s13578-018-0202-x.

490. Li F, Kaczor-Urbanowicz KE, Sun J, Majem B, Lo HC, Kim Y, et al. Characterization of human salivary extracellular RNA by next-generation sequencing. Clinical Chemistry. 2018;64(7):1085-95. doi: 10.1373/clinchem.2017.285072.

491. Li F, Koyano K, Marty V, Lee SH, Wong D, Xiao X, et al. Effectsofmaternal alcohol consumption on exosomal mirnas in amniotic fluids. Alcoholism: Clinical and Experimental Research. 2018;42:43A. doi: 10.1111/acer.13747.

492. Li G, Wang X, Li C, Hu S, Niu Z, Sun Q, et al. Piwi-interacting RNA1037 enhances chemoresistance and motility in human oral squamous cell carcinoma cells. OncoTargets and Therapy. 2019;12:10615-27. doi: 10.2147/OTT.S233322. PubMed Central PMCID: PMCDalian Meilun Biology Technology(China)

493. Li J, Tong Y, Sun Z, Chen Y, Wang Y, Zhou L, et al. A duplex-specific nuclease assisted photoelectrochemical biosensor based on MoS2@ReS2/Ti3C2 hybrid for ultrasensitive detection of colorectal cancer-related piRNA-31,143. Acta Biomaterialia. 2022;149:287-96. doi: 10.1016/j.actbio.2022.06.037.

494. Li J, Wang N, Zhang F, Jin S, Dong Y, Dong X, et al. PIWI-interacting RNAs are aberrantly

expressed and may serve as novel biomarkers for diagnosis of lung adenocarcinoma. Thoracic Cancer. 2021;12(18):2468-77. doi: 10.1111/1759-7714.14094.

495. Li PF, Chen SC, Xia T, Jiang XM, Shao YF, Xiao BX, et al. Non-coding RNAs and gastric cancer. World Journal of Gastroenterology. 2014;20(18):5411-9. doi: 10.3748/wjg.v20.i18.5411.

496. Li W, Gonzalez-Gonzalez M, Sanz-Criado L, Garcia-Carbonero N, Celdran A, Villarejo-Campos P, et al. A Novel PiRNA Enhances CA19-9 Sensitivity for Pancreatic Cancer Identification by Liquid Biopsy. Journal of Clinical Medicine. 2022;11(24). doi: 10.3390/jcm11247310.

497. Li XF, Ren P, Shen WZ, Jin X, Zhang J. The expression, modulation and use of cancer-testis antigens as potential biomarkers for cancer immunotherapy. American Journal of Translational Research. 2020;12(11):7002-19.

498. Li Y, Al Hallak MN, Philip PA, Azmi AS, Mohammad RM. Non-coding rnas in pancreatic cancer diagnostics and therapy: Focus on lncrnas, circrnas, and pirnas. Cancers. 2021;13(16). doi: 10.3390/cancers13164161.

499. Li Y, Dong Y, Zhao S, Gao J, Hao X, Wang Z, et al. Serum-derived piR-hsa-164586 of extracellular vesicles as a novel biomarker for early diagnosis of non-small cell lung cancer. Frontiers in Oncology. 2022;12. doi: 10.3389/fonc.2022.850363.

500. Li Y, Shan G, Teng ZQ, Wingo TS. Editorial: Non-Coding RNAs and Human Diseases. Frontiers in Genetics. 2020;11. doi: 10.3389/fgene.2020.00523.

501. Liao Y, Xu K. Epigenetic regulation of prostate cancer: The theories and the clinical implications. Asian Journal of Andrology. 2019;21(3):279-90. doi: 10.4103/aja.aja_53_18.

502. Lim MCJ, Baird AM, Aird J, Greene J, Kapoor D, Gray SG, et al. RNAs as candidate diagnostic and prognostic markers of prostate cancer - From cell line models to liquid biopsies. Diagnostics. 2018;8(3). doi: 10.3390/diagnostics8030060.

503. Lin C, Zheng L, Huang R, Yang G, Chen J, Li H. tRFs as potential exosome tRNA-derived fragment biomarkers for gastric carcinoma. Clinical Laboratory. 2020;66(6):961-9. doi: 10.7754/Clin.Lab.2019.190811.

504. Lin SC, Chiu CC, Chen ST, Chen YL, Lin PY, Hsiao SH, et al. Loss of PIWIL4 and piRNAs attenuate somatic methylome and genome Stability. Cancer Research. 2017;77(13). doi: 10.1158/1538-7445.AM2017-1398.

505. Lin TL, Berry A, Wise AL, Chavan H, Kasturi P, Fontes J, et al. Effect of hyperbaric oxygen treatment on chemotherapy sensitivity in acute myeloid leukemia. Journal of Clinical Oncology. 2016;34.

506. Lin TL, Wise A, Berry A, Fontes J, Kasturi P, Lipe BC, et al. Hyperbaric oxygen increases sensitivity to chemotherapy in acute leukemia. Blood. 2015;126(23):1399.

507. Lin X, Lo HC, Wong DTW, Xiao X. Noncoding RNAs in human saliva as potential disease biomarkers. Frontiers in Genetics. 2015;6(MAY). doi: 10.3389/fgene.2015.00175.

508. Lin Y, Zheng J, Lin D. PIWI-interacting RNAs in human cancer. Seminars in Cancer Biology. 2021;75:15-28. doi: 10.1016/j.semcancer.2020.08.012.

509. Linsen SEV, de Wit E, de Bruijn E, Cuppen E. Small RNA expression and strain specificity in

the rat. BMC Genomics. 2010;11(1). doi: 10.1186/1471-2164-11-249.

510. Liu CJ, Xie GY, Miao YR, Xia M, Wang Y, Lei Q, et al. EVAtlas: A comprehensive database for ncRNA expression in human extracellular vesicles. Nucleic Acids Research. 2022;50(D1):D111-D7. doi: 10.1093/nar/gkab668.

511. Liu Y, Dong Y, He X, Gong A, Gao J, Hao X, et al. piR-hsa-211106 Inhibits the Progression of Lung Adenocarcinoma Through Pyruvate Carboxylase and Enhances Chemotherapy Sensitivity. Frontiers in Oncology. 2021;11. doi: 10.3389/fonc.2021.651915. PubMed Central PMCID: PMCGenePharma(China)

512. Liu Y, Dou M, Song X, Dong Y, Liu S, Liu H, et al. The emerging role of the piRNA/piwi complex in cancer. Molecular Cancer. 2019;18(1). doi: 10.1186/s12943-019-1052-9.

513. Loganathan T, Doss C GP. Non-coding RNAs in human health and disease: potential function as biomarkers and therapeutic targets. Functional and Integrative Genomics. 2023;23(1). doi: 10.1007/s10142-022-00947-4. PubMed Central PMCID: PMCAchieve Life Sciences

514. Maguire S, Lohman GJS, Guan S. A low-bias and sensitive small RNA library preparation method using randomized splint ligation. Nucleic Acids Research. 2020;48(14). doi: 10.1093/nar/gkaa480.

515. Mahabady MK, Mirzaei S, Saebfar H, Gholami MH, Zabolian A, Hushmandi K, et al. Noncoding RNAs and their therapeutics in paclitaxel chemotherapy: Mechanisms of initiation, progression, and drug sensitivity. Journal of Cellular Physiology. 2022;237(5):2309-44. doi: 10.1002/jcp.30751.

516. Mai D, Ding P, Tan L, Zhang J, Pan Z, Bai R, et al. PIWI-interacting RNA-54265 is oncogenic and a potential therapeutic target in colorectal adenocarcinoma. Theranostics. 2018;8(19):5213-30. doi: 10.7150/thno.28001.

517. Mai D, Zheng Y, Guo H, Ding P, Bai R, Li M, et al. Serum piRNA-54265 is a new biomarker for early detection and clinical surveillance of Human Colorectal Cancer. Theranostics. 2020;10(19):8468-78. doi: 10.7150/thno.46241.

518. Maleki Dana P, Mansournia MA, Mirhashemi SM. PIWI-interacting RNAs: New biomarkers for diagnosis and treatment of breast cancer. Cell and Bioscience. 2020;10(1). doi: 10.1186/s13578-020-00403-5.

519. Markert L, Holdmann J, Klinger C, Kaufmann M, Schork K, Turewicz M, et al. Small RNAs as biomarkers to differentiate benign and malign prostate diseases: An alternative for transrectal punch biopsy of the prostate? PLoS ONE. 2021;16(3 March). doi: 10.1371/journal.pone.0247930.

520. Martinez D, Casadevall M, Ramirez J, Castellano J, Molins L, Marrades R, et al. Role of pir-796 in early stage non-small cell lung carcinoma. Modern Pathology. 2020;33(3):1800-1.

521. Martinez V, Marshall E, Firmino N, Minatel B, Bennewith K, Lam W. Hypoxia-induced modifications of the small non-coding RNA transcriptome delineates risk of recurrence in early-stage lung adenocarcinoma. Journal of Thoracic Oncology. 2017;12(11):S2270.

522. Martinez V, Ng K, Marshall E, Sage A, Minatel B, Jurisica I, et al. Arsenic promotes persistent alterations in the lung piRNA transcriptome to target epigenetic pathways. Journal of Thoracic Oncology. 2017;12(11):S1926-S7.

523. Martinez VD, Firmino NS, Marshall EA, Ng KW, Wadsworth BJ, Anderson C, et al. Non-coding

RNAs predict recurrence-free survival of patients with hypoxic tumours. Scientific reports. 2018;8(1):152. doi: 10.1038/s41598-017-18462-z.

524. Merkerova MD, Hrustincova A, Krejcik Z, Kundrat D, Szikszai K, Cermak J, et al. Circulating small noncoding rnas as novel semi-invasive markers of patient survival in myelodysplastic syndromes. Blood. 2019;134. doi: 10.1182/blood-2019-126520.

525. Meseure D, Lae M, Nicolas A, Vacher S, Chemlali W, Allory Y, et al. PIWIL proteins are potential biomarkers and promising therapeutic targets in invasive breast carcinomas. Modern Pathology. 2019;32(3).

526. Minatel B, Martinez V, Becker-Santos D, Marshall E, Ng K, Sage A, et al. Identification of oncofetal piRNAs in lung. Journal of Thoracic Oncology. 2017;12(11):S2271-S2.

527. Minatel B, Martinez V, Sage A, Marshall E, Tokar T, Becker-Santos D, et al. Large-Scale Discovery of Novel Human Oncofetal Transcripts in Lung. Journal of Thoracic Oncology. 2018;13(10):S432. doi: 10.1016/j.jtho.2018.08.499.

528. Mokarram P, Niknam M, Sadeghdoust M, Aligolighasemabadi F, Siri M, Dastghaib S, et al. PIWI interacting RNAs perspectives: a new avenues in future cancer investigations. Bioengineered. 2021;12(2):10401-19. doi: 10.1080/21655979.2021.1997078.

529. Movahedpour A, Khatami SH, Karami N, Vakili O, Naeli P, Jamali Z, et al. Exosomal noncoding RNAs in prostate cancer. Clinica Chimica Acta. 2022;537:127-32. doi: 10.1016/j.cca.2022.10.018.

530. Nayak R, Chattopadhyay T, Gupta P, Mallick B. Integrative analysis of small non-coding RNAs predicts a piRNA/miRNA-CCND1/BRAF/HRH1/ATXN3 regulatory circuit that drives oncogenesis in glioblastoma. Molecular Omics. 2023;19(3):252-61. doi: 10.1039/d2mo00245k.

531. Nellore J, Narayan AS, Sunkar S, Valli Nachiyar C, Jayakrishna T, Peela S. Circulating biomarkers for the early diagnosis of gastrointestinal cancers. Critical Reviews in Oncogenesis. 2020;25(4):335-54. doi: 10.1615/CritRevOncog.2020036205.

532. Ng KW, Anderson C, Marshall EA, Minatel BC, Enfield KSS, Saprunoff HL, et al. Piwi-interacting RNAs in cancer: Emerging functions and clinical utility. Molecular Cancer. 2016;15(1). doi: 10.1186/s12943-016-0491-9.

533. Nicu AT, Medar C, Chifiriuc MC, Gradisteanu Pircalabioru G, Burlibasa L. Epigenetics and Testicular Cancer: Bridging the Gap Between Fundamental Biology and Patient Care. Frontiers in Cell and Developmental Biology. 2022;10. doi: 10.3389/fcell.2022.861995.

534. Nie Y, F Wilson A, DeFalco T, Meetei AR, Namekawa SH, Pang Q. FANCD2 is required for the repression of germline transposable elements. Reproduction. 2020;159(6):659-68. doi: 10.1530/REP-19-0436.

535. Outeiro-Pinho G, Barros-Silva D, Correia MP, Henrique R, Jerónimo C. Renal cell tumors: Uncovering the biomarker potential of ncRNAs. Cancers. 2020;12(8):1-30. doi: 10.3390/cancers12082214.

536. Ozawa T, Toiyama Y, Takahashi N, Nagasaka T, Fujiwara T, Yamada Y, et al. Plasma levels of pirnas as biomarkers for prognosis and predicting tumor recurrence in colorectal cancer patients. Gastroenterology. 2017;152(5):S152.

537. Pall GS, Codony-Servat C, Byrne J, Ritchie L, Hamilton A. Carbodiimide-mediated cross-linking of RNA to nylon membranes improves the detection of siRNA, miRNA and piRNA by

northern blot. Nucleic Acids Research. 2007;35(8). doi: 10.1093/nar/gkm112.

538. Pall GS, Hamilton AJ. Improved northern blot method for enhanced detection of small RNA. Nature Protocols. 2008;3(6):1077-84. doi: 10.1038/nprot.2008.67.

539. Papait R, Kunderfranco P, Stirparo GG, Latronico MVG, Condorelli G. Long noncoding RNA: A new player of heart failure? Journal of Cardiovascular Translational Research. 2013;6(6):876-83. doi: 10.1007/s12265-013-9488-6.

540. Parker JS, Roe SM, Barford D. Molecular mechanism of target RNA transcript recognition by argonaute-guide complexes. 2006. p. 45-50.

541. Pathania AS, Prathipati P, Pandey MK, Byrareddy SN, Coulter DW, Gupta SC, et al. The emerging role of non-coding RNAs in the epigenetic regulation of pediatric cancers. Seminars in Cancer Biology. 2022;83:227-41. doi: 10.1016/j.semcancer.2021.04.015.

542. Peng W, Zhu J, Liu D, Qian P, Zhang Q, Li H, et al. Clinical value of piR-36026 and piR-651 in esophageal squamous cell carcinoma. Journal of Clinical Oncology. 2020;38(15). doi: 10.1200/JCO.2020.38.15_suppl.e15265.

543. Peng X, Frohman MA. Mammalian phospholipase D physiological and pathological roles. Acta Physiologica. 2012;204(2):219-26. doi: 10.1111/j.1748-1716.2011.02298.x.

544. Perera BPU, Faulk C, Svoboda LK, Goodrich JM, Dolinoy DC. The role of environmental exposures and the epigenome in health and disease. Environmental and Molecular Mutagenesis. 2020;61(1):176-92. doi: 10.1002/em.22311.

545. Perera BPU, Svoboda LK, Dolinoy DC. Genomic tools for environmental epigenetics and implications for public health. Current Opinion in Toxicology. 2019;18:27-33. doi: 10.1016/j.cotox.2019.02.008.

546. Pian C, Chen YY, Zhang J, Chen Z, Zhang GL, Li Q, et al. V-ELMpiRNAPred: Identification of human piRNAs by the voting-based extreme learning machine (V-ELM) with a new hybrid feature. Journal of bioinformatics and computational biology. 2017;15(1):1650046. doi: 10.1142/S0219720016500463.

547. Plissonnier ML, Herzog K, Levrero M, Zeisel MB. Non-coding RNAs and hepatitis C virus-induced hepatocellular carcinoma. Viruses. 2018;10(11). doi: 10.3390/v10110591.

548. Pokorna JP, Trachtova K, Kazda T, Slaby O. Small RNA-seq analysis of PIWI-interacting RNAs in glioblastoma stem cells: Identification of new therapeutic targets in glioblastoma patients. Cancer Research. 2022;82(12). doi: 10.1158/1538-7445.AM2022-908.

549. Qu A, Wang W, Yang Y, Zhang X, Dong Y, Zheng G, et al. A serum piRNA signature as promising non-invasive diagnostic and prognostic biomarkers for colorectal cancer. Cancer Management and Research. 2019;11:3703-20. doi: 10.2147/CMAR.S193266.

550. Rahimy E, Kuo SZ, Ongkeko WM. Evaluation of non-coding RNAs as potential targets in head and neck squamous cell carcinoma cancer stem cells. Current Drug Targets. 2014;15(13):1247-60. doi: 10.2174/1389450115666141024113446.

551. Rajasethupathy P, Antonov I, Sheridan R, Frey S, Sander C, Tuschl T, et al. A role for neuronal piRNAs in the epigenetic control of memory-related synaptic plasticity. Cell. 2012;149(3):693-707. doi: 10.1016/j.cell.2012.02.057.

552. Ramalho-Carvalho J, Fromm B, Henrique R, Jerónimo C. Deciphering the function of non-

coding RNAs in prostate cancer. Cancer and Metastasis Reviews. 2016;35(2):235-62. doi: 10.1007/s10555-016-9628-y.

553. Ran Z, Wu S, Ma Z, Chen X, Liu J, Yang J. Advances in exosome biomarkers for cervical cancer. Cancer Medicine. 2022;11(24):4966-78. doi: 10.1002/cam4.4828.

554. Ran Z, Yang J, Liu Y, Chen X, Ma Z, Wu S, et al. GlioMarker: An integrated database for knowledge exploration of diagnostic biomarkers in gliomas. Frontiers in Oncology. 2022;12. doi: 10.3389/fonc.2022.792055.

555. Rapisuwon S, Vietsch EE, Wellstein A. Circulating biomarkers to monitor cancer progression and treatment. Computational and Structural Biotechnology Journal. 2016;14:211-22. doi: 10.1016/j.csbj.2016.05.004.

556. Ray SK, Mukherjee S. Piwi-interacting RNAs (piRNAs) and Colorectal Carcinoma: Emerging Non-invasive diagnostic Biomarkers with Potential Therapeutic Target Based Clinical Implications. Current Molecular Medicine. 2023;23(4):300-11. doi: 10.2174/1566524022666220124102616.

557. Raza A, Khan AQ, Inchakalody VP, Mestiri S, Yoosuf ZSKM, Bedhiafi T, et al. Dynamic liquid biopsy components as predictive and prognostic biomarkers in colorectal cancer. Journal of Experimental and Clinical Cancer Research. 2022;41(1). doi: 10.1186/s13046-022-02318-0.

558. Razavi ZS, Tajiknia V, Majidi S, Ghandali M, Mirzaei HR, Rahimian N, et al. Gynecologic cancers and non-coding RNAs: Epigenetic regulators with emerging roles. Critical Reviews in Oncology/Hematology. 2021;157. doi: 10.1016/j.critrevonc.2020.103192.

559. Reis AHO, Vargas FR, Lemos B. Biomarkers of genome instability and cancer epigenetics. Tumor Biology. 2016;37(10):13029-38. doi: 10.1007/s13277-016-5278-5.

560. Rhim J, Baek W, Seo Y, Kim JH. From Molecular Mechanisms to Therapeutics: Understanding MicroRNA-21 in Cancer. Cells. 2022;11(18). doi: 10.3390/cells11182791.

561. Rinaldi A, Giurato G, Hashim A, Rizzo F, Marchese G, Ravo M, et al. An innovative and complete workflow for smallRNA-Seq data analysis. American Journal of Pathology. 2014;184(9):S2.

562. Ritter A, Hirschfeld M, Berner K, Rücker G, Jäger M, Weiss D, et al. Circulating non-coding RNA-biomarker potential in neoadjuvant chemotherapy of triple negative breast cancer? International Journal of Oncology. 2020;56(1):47-68. doi: 10.3892/ijo.2019.4920.

563. Rivero-Segura NA, Bello-Chavolla OY, Barrera-Vázquez OS, Gutierrez-Robledo LM, Gomez-Verjan JC. Promising biomarkers of human aging: In search of a multi-omics panel to understand the aging process from a multidimensional perspective. Ageing Research Reviews. 2020;64. doi: 10.1016/j.arr.2020.101164.

564. Rizzo F, Rinaldi A, Coviello E, Sellitto A, Cracas DG, Ricciardi L, et al. Identification of specific PIWI-interacting small noncoding RNA (piRNA) expression patterns during hepatocarcinogenesis. American Journal of Pathology. 2016;186(10):S18.

565. Robinson H, Roberts MJ, Gardiner RA, Hill MM. Extracellular vesicles for precision medicine in prostate cancer – Is it ready for clinical translation? Seminars in Cancer Biology. 2023;89:18-29. doi: 10.1016/j.semcancer.2023.01.003.

566. Rounge TB, Furu K, Skotheim R, Haugen TB, Enerly E, Grotmol T. Small RNA profiling of human testicular germ cell tumor tissue samples shows abundant tRNA fragments and global

loss of piRNA. Cancer Research. 2015;75(15). doi: 10.1158/1538-7445.AM2015-LB-296.

567. Rounge TB, Furu K, Skotheim RI, Enerly E, Haugen TB, Grotmol T. Genome-wide changes in expression of small RNAs in human testis cancer tissue. Cancer Prevention Research. 2015;8(10). doi: 10.1158/1940-6215.PREV-14-A01.

568. Rounge TB, Umu SU, Keller A, Meese E, Ursin G, Tretli S, et al. Impact of age, sex, smoking, body mass and physical activity on circulating small non-coding RNA expression profiles. Cancer Research. 2018;78(13). doi: 10.1158/1538-7445.AM2018-524.

569. Roy J, Das B, Jain N, Mallick B. PIWI-interacting RNA 39980 promotes tumor progression and reduces drug sensitivity in neuroblastoma cells. Journal of Cellular Physiology. 2020;235(3):2286-99. doi: 10.1002/jcp.29136.

570. Rui T, Wang K, Xiang A, Guo J, Tang N, Jin X, et al. Serum Exosome-Derived piRNAs Could Be Promising Biomarkers for HCC Diagnosis. International journal of nanomedicine. 2023;18:1989-2001. doi: 10.2147/IJN.S398462.

571. Sabbah NA, Abdalla WM, Mawla WA, Abdalmonem N, Gharib AF, Abdul-Saboor A, et al. PiRNA-823 is a unique potential diagnostic non-invasive biomarker in colorectal cancer patients. Genes. 2021;12(4). doi: 10.3390/genes12040598.

572. Sadik N, Cruz L, Gurtner A, Rodosthenous RS, Dusoswa SA, Ziegler O, et al. Extracellular RNAs: A new awareness of old perspectives. 2018. p. 1-15.

573. Sadovska L, Zayakin P, Eglītis K, Endzeliņš E, Radoviča-Spalviņa I, Avotiņa E, et al. Comprehensive characterization of RNA cargo of extracellular vesicles in breast cancer patients undergoing neoadjuvant chemotherapy. Frontiers in Oncology. 2022;12. doi: 10.3389/fonc.2022.1005812.

574. Saggese P, Ravo M, Cordella A, Rinaldi A, Chetta M, Alexandrova E, et al. Deregulation of small non coding RNAs in endometrial carcinogenesis. American Journal of Pathology. 2014;184(9):S7.

575. Saleem S, Sarfraz U, Saeed S, Maryam A, Iqbal MJ. Role of Molecular Biomarkers in Colorectal Cancer. Iranian Journal of Blood and Cancer. 2022;14(3):41-56.

576. Salimimoghadam S, Taefehshokr S, Loveless R, Teng Y, Bertoli G, Taefehshokr N, et al. The role of tumor suppressor short non-coding RNAs on breast cancer. Critical Reviews in Oncology/Hematology. 2021;158. doi: 10.1016/j.critrevonc.2020.103210.

577. Sana J, Faltejskova P, Svoboda M, Slaby O. Novel classes of non-coding RNAs and cancer. Journal of Translational Medicine. 2012;10(1). doi: 10.1186/1479-5876-10-103.

578. Sandoval-Bórquez A, Saavedra K, Carrasco-Avino G, Garcia-Bloj B, Fry J, Wichmann I, et al. Noncoding Genomics in Gastric Cancer and the Gastric Precancerous Cascade: Pathogenesis and Biomarkers. Disease Markers. 2015;2015. doi: 10.1155/2015/503762.

579. Sarraf JS, Puty TC, da Silva EM, Allen TSR, Sarraf YS, de Carvalho LEW, et al. Noncoding RNAs and colorectal cancer: A general overview. MicroRNA. 2020;9(5):336-45. doi: 10.2174/2211536609666201221124608.

580. Sato K, Baiocchi L, Kennedy L, Zhang W, Ekser B, Glaser S, et al. Current advances in basic and translational research of cholangiocarcinoma. Cancers. 2021;13(13). doi: 10.3390/cancers13133307.

581. Seyeddokht A, Aslaminejad AA, Masoudi-Nejad A, Nassiri M, Zahiri J, Sadeghi B. Computational detection of piRNA in human using support vector machine. Avicenna Journal of Medical Biotechnology. 2016;8(1):36-41.

582. Seyhan AA. RNAi: A potential new class of therapeutic for human genetic disease. Human Genetics. 2011;130(5):583-605. doi: 10.1007/s00439-011-0995-8.

583. Shen S. Editorial: Integrative Approaches to Analyze Cancer Based on Multi-Omics. Frontiers in Genetics. 2022;13. doi: 10.3389/fgene.2022.1057408.

584. Shi X, Ren S, Sun Y. How can plasma RNA be used to diagnose prostate cancer? Expert Review of Anticancer Therapy. 2017;17(1):5-7. doi: 10.1080/14737140.2017.1266262.

585. Singh RS, Arna AB, Dong H, Yadav M, Aggarwal A, Wu Y. Structure-function analysis of DEAD-box helicase DDX43. Methods. 2022;204:286-99. doi: 10.1016/j.ymeth.2022.03.002.

586. Slaby O. Non-coding RNAs as biomarkers for colorectal cancer screening and early detection. 2016. p. 153-70.

587. Sohn EJ, Oh SO. P-Element-Induced Wimpy Testis Proteins and P-Element-Induced Wimpy Testis-Interacting RNAs Expression in Ovarian Cancer Stem Cells. Genetic Testing and Molecular Biomarkers. 2023;27(2):56-64. doi: 10.1089/gtmb.2022.0113.

588. Sonea L, Buse M, Gulei D, Onaciu A, Simon I, Braicu C, et al. Decoding the emerging patterns exhibited in non-coding rnas characteristic of lung cancer with regard to their clinical significance. Current Genomics. 2018;19(4):258-78. doi: 10.2174/1389202918666171005100124.

589. Su JF, Zhao F, Gao ZW, Hou YJ, Li YY, Duan LJ, et al. piR-823 demonstrates tumor oncogenic activity in esophageal squamous cell carcinoma through DNA methylation induction via DNA methyltransferase 3B. Pathology Research and Practice. 2020;216(4). doi: 10.1016/j.prp.2020.152848.

590. Suárez B, Solé C, Márquez M, Nanetti F, Lawrie CH. Circulating MicroRNAs as Cancer Biomarkers in Liquid Biopsies. 2022. p. 23-73.

591. Sun C, Fu Z, Wang S, Li J, Li Y, Zhang Y, et al. Roles of tRNA-derived fragments in human cancers. Cancer Letters. 2018;414:16-25. doi: 10.1016/j.canlet.2017.10.031.

592. Sun G, Yang Y, Liu J, Gao Z, Xu T, Chai J, et al. Cancer stem cells in esophageal squamous cell carcinoma. Pathology Research and Practice. 2022;237. doi: 10.1016/j.prp.2022.154043.

593. Sun J, Lu H, Liang W, Zhao G, Ren L, Hu D, et al. Endothelial TFEB (Transcription Factor EB) Improves Glucose Tolerance via Upregulation of IRS (Insulin Receptor Substrate) 1 and IRS2. Arteriosclerosis, Thrombosis, and Vascular Biology. 2021;41(2):783-95. doi: 10.1161/ATVBAHA.120.315310.

594. Sundar I, Li D, Rahman I. Rna-sequencing analysis of human plasma-derived extracellular vesicles as potential circulating biomarkers in chronic obstructive pulmonary disease. American Journal of Respiratory and Critical Care Medicine. 2019;199(9).

595. Suri K, Bubier JA, Wiles MV, Shultz LD, Amiji MM, Hosur V. Role of microRNA in inflammatory bowel disease: Clinical evidence and the development of preclinical animal models. Cells. 2021;10(9). doi: 10.3390/cells10092204.

596. Szilágyi M, Pös O, Márton É, Buglyó G, Soltész B, Keserű J, et al. Circulating cell-free nucleic

acids: Main characteristics and clinical application. International Journal of Molecular Sciences. 2020;21(18):1-20. doi: 10.3390/ijms21186827.

597. Tamkovich SN, Tutanov OS, Laktionov PP. Exosomes: Generation, structure, transport, biological activity, and diagnostic application. Biochemistry (Moscow) Supplement Series A: Membrane and Cell Biology. 2016;10(3):163-73. doi: 10.1134/S1990747816020112.

598. Tan L, Mai D, Zhang B, Jiang X, Zhang J, Bai R, et al. PIWI-interacting RNA-36712 restrains breast cancer progression and chemoresistance by interaction with SEPW1 pseudogene SEPW1P RNA. Molecular Cancer. 2019;18(1). doi: 10.1186/s12943-019-0940-3.

599. Tan Z, Li W, Cheng X, Zhu Q, Zhang X. Non-Coding RNAs in the Regulation of Hippocampal Neurogenesis and Potential Treatment Targets for Related Disorders. Biomolecules. 2023;13(1). doi: 10.3390/biom13010018.

600. Taylor DH, Chu ETJ, Spektor R, Soloway PD. Long non-coding RNA regulation of reproduction and development. Molecular Reproduction and Development. 2015;82(12):932-56. doi: 10.1002/mrd.22581.

601. Tong Y, Guan B, Sun Z, Dong X, Chen Y, Li Y, et al. Ratiometric fluorescent detection of exosomal piRNA-823 based on Au NCs/UiO-66-NH2 and target-triggered rolling circle amplification. Talanta. 2023;257:124307. doi: 10.1016/j.talanta.2023.124307.

602. Torkashvand S, Damavandi Z, Mirzaei B, Tavallaei M, Vasei M, Mowla SJ. Decreased expression of bioinformatically predicted piwil2-targetting microRNAs, miR-1267 and miR-2276 in breast cancer. Archives of Iranian Medicine. 2016;19(6):420-5.

603. Tosar JP, García-Silva MR, Cayota A. Circulating SNORD57 rather than piR-54265 is a promising biomarker for colorectal cancer: Common pitfalls in the study of somatic piRNAs in cancer. RNA. 2021;27(4):403-10. doi: 10.1261/rna.078444.120.

604. Umu SU, Langseth H, Keller A, Meese E, Helland Å, Lyle R, et al. A 10-year prediagnostic follow-up study shows that serum RNA signals are highly dynamic in lung carcinogenesis. Molecular Oncology. 2020;14(2):235-47. doi: 10.1002/1878-0261.12620.

605. Umu SU, Lyle R, Langseth H, Rounge TB. Natural variation in serum small non-coding RNAs-potential biomarkers of cancer. ESMO Open. 2018;3:A263-A4. doi: 10.1136/esmoopen-2018-EACR25.623.

606. Vafaei S, Fattahi F, Sahlolbei M, Kiani J, Yazdanpanah A, Madjd Z. Dynamic signature of trna-derived small rnas in cancer pathogenesis as a promising valuable approach. Critical Reviews in Eukaryotic Gene Expression. 2020;30(5):391-410. doi: 10.1615/critreveukaryotgeneexpr.2020035372.

607. Vychytilova-Faltejskova P, Stitkovcova K, Radova L, Sachlova M, Kosarova Z, Slaba K, et al. Circulating PIWI-interacting RNAs piR-5937 and piR-28876 are promising diagnostic biomarkers of colon cancer. Cancer Epidemiology Biomarkers and Prevention. 2018;27(9):1019-28. doi: 10.1158/1055-9965.EPI-18-0318.

608. Wang B, Yang C, Zhou C, Xiao S, Li H. Knowledge atlas and emerging trends on ncRNAs of osteosarcoma: A bibliometric analysis. Frontiers in Endocrinology. 2022;13. doi: 10.3389/fendo.2022.1028031.

609. Wang H, Shi B, Zhang X, Shen P, He Q, Yin M, et al. Exosomal hsa-piR1089 promotes proliferation and migration in neuroblastoma via targeting KEAP1. Pathology Research and

Practice. 2023;241. doi: 10.1016/j.prp.2022.154240.

610. Wang J, Song YX, Ma B, Wang JJ, Sun JX, Chen XW, et al. Regulatory roles of non-coding RNAs in colorectal cancer. International Journal of Molecular Sciences. 2015;16(8):19886-919. doi: 10.3390/ijms160819886.

611. Wang K, Wang T, Gao XQ, Chen XZ, Wang F, Zhou LY. Emerging functions of piwi-interacting RNAs in diseases. Journal of Cellular and Molecular Medicine. 2021;25(11):4893-901. doi: 10.1111/jcmm.16466.

612. Wang Y, Gable T, Ma MZ, Clark D, Zhao J, Zhang Y, et al. A piRNA-like Small RNA Induces Chemoresistance to Cisplatin-Based Therapy by Inhibiting Apoptosis in Lung Squamous Cell Carcinoma. Molecular Therapy - Nucleic Acids. 2017;6:269-78. doi: 10.1016/j.omtn.2017.01.003.

613. Wang Z, Yang H, Ma D, Mu Y, Tan X, Hao Q, et al. Serum PIWI-interacting RNAs piR-020619 and piR-020450 are promising novel biomarkers for early detection of colorectal cancer. Cancer Epidemiology Biomarkers and Prevention. 2020;29(5):990-8. doi: 10.1158/1055-9965.EPI-19-1148.

614. Wang ZY, Wen ZJ, Xu HM, Zhang Y, Zhang YF. Exosomal noncoding RNAs in central nervous system diseases: biological functions and potential clinical applications. Frontiers in Molecular Neuroscience. 2022;15. doi: 10.3389/fnmol.2022.1004221.

615. Weng W, Liu N, Toiyama Y, Kusunoki M, Nagasaka T, Fujiwara T, et al. Novel evidence for a PIWI-interacting RNA (piRNA) as an oncogenic mediator of disease progression, and a potential prognostic biomarker in colorectal cancer. Molecular Cancer. 2018;17(1). doi: 10.1186/s12943-018-0767-3.

616. Witasp A, Van Craenenbroeck AH, Shiels PG, Ekström TJ, Stenvinkel P, Nordfors L. Current epigenetic aspects the clinical kidney researcher should embrace. Clinical Science. 2017;131(14):1649-67. doi: 10.1042/CS20160596.

617. Xiao L, Wang J, Ju S, Cui M, Jing R. Disorders and roles of tsRNA, snoRNA, snRNA and piRNA in cancer. Journal of Medical Genetics. 2022. doi: 10.1136/jmedgenet-2021-108327.

618. Xu J, Yang X, Zhou Q, Zhuang J, Han S. Biological significance of piRNA in liver cancer: a review. Biomarkers. 2020:1-5. doi: 10.1080/1354750X.2020.1794041.

619. Xue M, Shi M, Xie J, Zhang J, Jiang L, Deng X, et al. Serum tRNA-derived small RNAs as potential novel diagnostic biomarkers for pancreatic ductal adenocarcinoma. American Journal of Cancer Research. 2021;11(3):837-48.

620. Yan IK, David W, Mohankumar S, Nix S, Haga H, Asmann YW, et al. Identification of novel biomarkers of cholangiocarcinoma using RNA sequencing of extracellular RNA (exRNA) in bile. Hepatology. 2015;62:391A-2A. doi: 10.1002/hep.28212.

621. Yang L, Ge Y, Cheng D, Nie Z, Lv Z. Detection of piRNAs in whitespotted bamboo shark liver. Gene. 2016;590(1):51-6. doi: 10.1016/j.gene.2016.06.008.

622. Yang L, Zhang X, Hu G. Circulating non-coding RNAs as new biomarkers and novel therapeutic targets in colorectal cancer. Clinical and Translational Oncology. 2021;23(11):2220-36. doi: 10.1007/s12094-021-02639-0.

623. Yasui Y, Mihe K, Oyama T, Tanaka T. Colorectal carcinogensis and suppression of tumor development by inhibition of enzymes and molecular targets. Current Enzyme Inhibition.

2009;5(1):1-26. doi: 10.2174/157340809787314247.

624. Yin J, Jiang HQ. PiRNA expression profiling in colorectal cancer using deep sequencing. Journal of Digestive Diseases. 2018;19:28-9. doi: 10.1111/1751-2980.12664.

625. Yin P, Wang Z, Chen Y, Wen ZQ, Hong HH, Mao YL. Plasma concentration of piRNAs in breast cancer and its association with metastasis. European Journal of Gynaecological Oncology. 2021;42(2):307-10. doi: 10.31083/j.ejgo.2021.02.2179.

626. Yousefi B, Sadoughi F, Asemi Z, Mansournia MA, Hallajzadeh J. Novel Perspectives for the Diagnosis and Treatment of Gynecological Cancers using Dysregulation of PIWI Protein and PiRNAs as Biomarkers. Current medicinal chemistry. 2023. doi: 10.2174/0929867330666230214101837.

627. Zaporozhchenko IA, Ponomaryova AA, Rykova EY, Laktionov PP. The potential of circulating cell-free RNA as a cancer biomarker: challenges and opportunities. Expert Review of Molecular Diagnostics. 2018;18(2):133-45. doi: 10.1080/14737159.2018.1425143.

628. Zeng MS. Noncoding RNAs in cancer diagnosis. 2016. p. 391-427.

629. Zeuschner P, Linxweiler J, Junker K. Non-coding RNAs as biomarkers in liquid biopsies with a special emphasis on extracellular vesicles in urological malignancies. Expert Review of Molecular Diagnostics. 2020;20(2):151-67. doi: 10.1080/14737159.2019.1665998.

630. Zhang C. Novel functions for small RNA molecules. Current Opinion in Molecular Therapeutics. 2009;11(6):641-51.

631. Zhang LM, Gao QX, Chen J, Li B, Li MM, Zheng L, et al. A universal catalytic hairpin assembly system for direct plasma biopsy of exosomal PIWI-interacting RNAs and microRNAs. Analytica Chimica Acta. 2022;1192. doi: 10.1016/j.aca.2021.339382.

632. Zhang W, Kataoka N, Guan X. Editorial: Non-Coding RNAs in Breast Cancer. Frontiers in Oncology. 2021;11. doi: 10.3389/fonc.2021.789798.

633. Zhang Y, Kim JS, Wang TZ, Newton RU, Galvão DA, Gardiner RA, et al. Potential Role of Exercise Induced Extracellular Vesicles in Prostate Cancer Suppression. Frontiers in Oncology. 2021;11. doi: 10.3389/fonc.2021.746040.

634. Zhao C, Tolkach Y, Schmidt D, Toma M, Muders MH, Kristiansen G, et al. Mitochondrial PIWI-interacting RNAs are novel biomarkers for clear cell renal cell carcinoma. World journal of urology. 2019;37(8):1639-47. doi: 10.1007/s00345-018-2575-1.

635. Zhao N, Deng Q, Zhu C, Zhang B. Mucus piRNAs profiles of Vibrio harveyi-infected Cynoglossus semilaevis: A hint for fish disease monitoring. Journal of Fish Diseases. 2022;45(1):165-75. doi: 10.1111/jfd.13546.

636. Zhou J, Xie H, Liu J, Huang R, Xiang Y, Tian D, et al. PIWI-interacting RNAs: Critical roles and therapeutic targets in cancer. Cancer Letters. 2023;562. doi: 10.1016/j.canlet.2023.216189.

637. Zhou X, Ao X, Jia Z, Li Y, Kuang S, Du C, et al. Non-coding RNA in cancer drug resistance: Underlying mechanisms and clinical applications. Frontiers in Oncology. 2022;12. doi: 10.3389/fonc.2022.951864.

638. Zhou X, Liu J, Meng A, Zhang L, Wang M, Fan H, et al. Gastric juice piR-1245: A promising prognostic biomarker for gastric cancer. Journal of Clinical Laboratory Analysis. 2020;34(4).

doi: 10.1002/jcla.23131.

639. Ziogas D. The emerging role of miRNAs in translational cancer medicine. Gastric and Breast Cancer. 2012;11(3):180-95. doi: 10.2122/gbc.2012.0227.

640. Zivarpour P, Asemi Z, Jamilian H, Hallajzadeh J. PiRNAs and PIWI proteins as new biomarkers for diagnosis and treatment of liver cancer. Gene Reports. 2021;23. doi: 10.1016/j.genrep.2021.101103.

641. Zuo Y, Liang Y, Zhang J, Hao Y, Li M, Wen Z, et al. Transcriptome Analysis Identifies Piwi-Interacting RNAs as Prognostic Markers for Recurrence of Prostate Cancer. Frontiers in Genetics. 2019;10. doi: 10.3389/fgene.2019.01018.
